# Very high frequency (263 GHz) pulse EPR spectroscopy of high spin transition metal centers

**DOI:** 10.1038/s41467-026-78076-w

**Published:** 2026-10-01

**Authors:** Zikri Hasanbasri, Shasha Qiu, Guodong Rao, Amanda Caceres, Erica Debley, Yuan Zheng, Elizabeth M. Nolan, Marie Heffern, Paul Stucky, Neville C. Luhmann Jr, R. David Britt

**Affiliations:** 1 Department of Chemistry, University of California Davis, 1 Shields Ave, Davis, CA USA; 2 Department of Electrical and Computer Engineering, University of California Davis, 1 Shields Ave, Davis, CA USA; 3 Department of Chemistry, Massachusetts Institute of Technology, Cambridge, MA USA

**Keywords:** Characterization and analytical techniques, Inorganic chemistry, Physical chemistry

## Abstract

Pulse Electron Paramagnetic Resonance (EPR) spectroscopy provides powerful tools for examining species with unpaired electrons, such as organic radicals and metal ions, in a wide variety of interesting systems. However, the lack of high power pulse amplifiers above approximately 100 GHz has limited its applicability at very high frequencies and magnetic fields. Here we describe the use of a new 10 W high frequency traveling wave vacuum tube amplifier employed in a pulse EPR spectrometer operating over a wide frequency bandwidth centered at 263 GHz. Very high frequency pulse EPR is of particular utility for studying spin S > 1/2 species, given that large broadening effects of “zero-field splitting” interactions often dominate EPR spectra obtained at low frequency. Improvements obtained at 263 GHz are illustrated for a series of high spin (S=5/2) Mn(II) complexes starting with Mn(II) impurities in a diamagnetic host up though Mn(II) bound in metal sequestering proteins. The instrument has sufficient mm-wave power to obtain high signal-to-noise electron spin echoes without a resonator, which offers a number of advantages as described herein.

## Abstract (web summary)

Pulse electron paramagnetic resonance spectroscopy is performed with a 263 GHz pulse spectrometer powered by a 10 W traveling wave tube amplifier, operating without a resonator to allow larger sample volumes and flexible geometries and high sensitivity, as shown in a series of high spin Mn(II) samples.

## Introduction

Electron Paramagnetic Resonance (EPR) spectroscopy is an important magnetic resonance technique for studying paramagnetic species in chemistry, physics, materials, and biology. On a spin to spin basis, EPR is far more sensitive than nuclear magnetic resonance (NMR) because of the much larger magnetic moments of unpaired electron spin systems compared to those of nuclear spins. Much early EPR spectroscopy was performed at X-band frequencies ( ≈ 9.4 GHz) due to the ready availability of klystron vacuum tube microwave sources at that frequency band, originally developed for radar systems. Electromagnets for use at the corresponding  ≈ 0.35 T resonant magnetic fields are also rugged and reliable. Although there has been great advancement towards higher microwave frequencies over the years, the majority of EPR systems worldwide still operate in the X-band frequency range, offering simple and reliable performance.

**Fig. 1:**
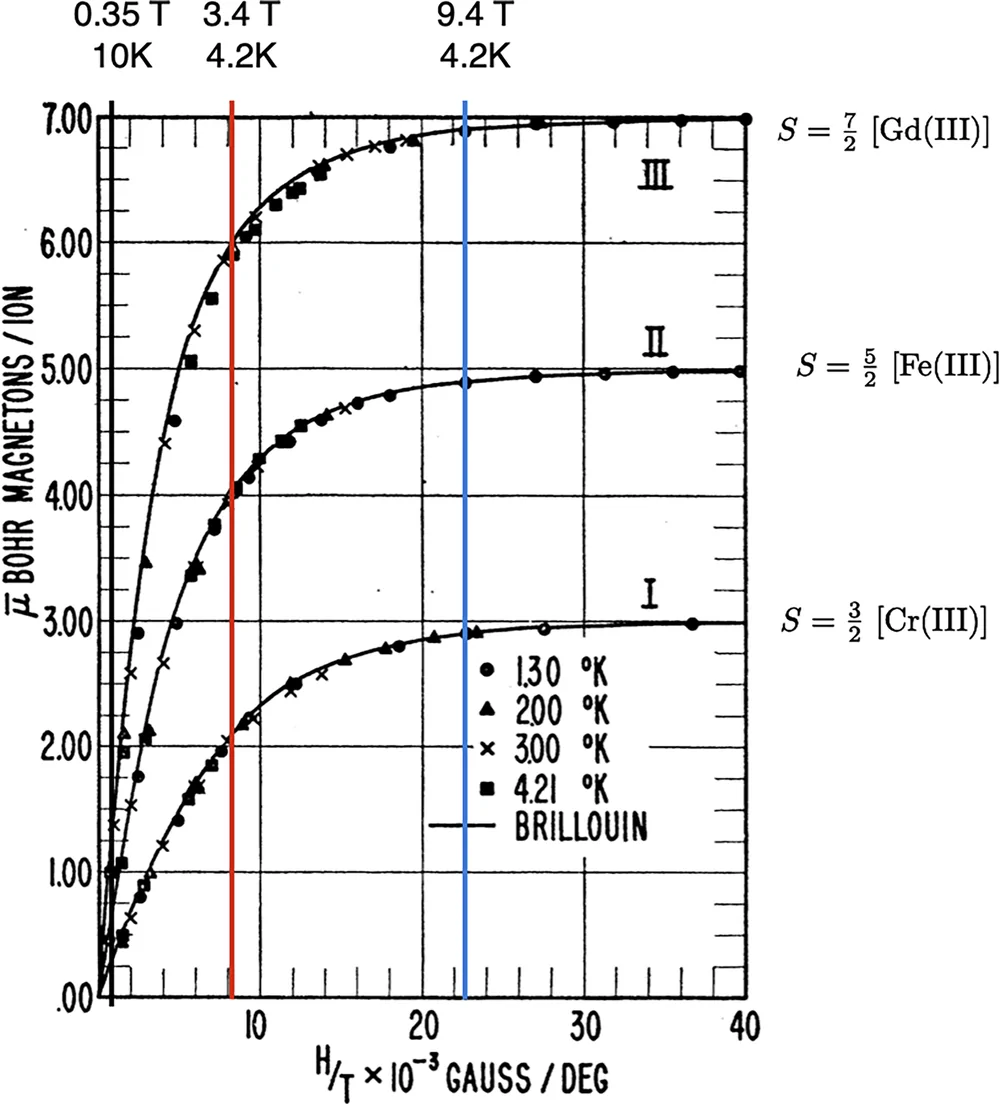
Experimental magnetic moments for a selection of High Spin (H.S.) paramagnetic ions compared with the theoretical prediction (the Brillouin function). The left (black) bar indicates typical low temperature (10 K) X-band EPR conditions for g = 2 (*H *= 0.35 T). The center (red) bar corresponds to W-band (94 GHz) for *T*=4.2 K, and right blue bar indicates the magnetization for 263 GHz (*H *= 9.4 T and *T *= 4.2 K). Figure adapted from^63^.

Conventional magnetic resonance begins with equilibrium magnetization *M*_0_ that is determined by spin type and the magnetic field *H* and temperature *T*. The application of oscillating magnetic fields drives the resonance, with resulting signals that scale with the initial magnetization. Figure 1 shows the equilibrium magnetization for a set of high spin (H.S.) ions (total spin *S*) along with the match of the experimental data to the Brillouin function^1^ that describes this field and temperature dependence, where *β* is the Bohr magneton for electrons, *S* is the total spin quantum number, and *g* is a dimensionless constant for a given electronic spin system:

1 $${M}_{0}\propto (S+1/2)\coth (S+1/2)\left(\dfrac{g\beta H}{kT}\right)-1/2\coth \left(\dfrac{g\beta H}{2kT}\right)$$

The leftmost (black) vertical line in Fig. 1 indicates typical X-band EPR conditions for a g = 2 resonance at a 0.35 T field and a 10 K cryogenic temperature, clearly in the linear (*H*/*T*) “Curie Law” regime far from saturation of the electron spin magnetization. Thus EPR performed at higher frequency/field will offer increased electron spin magnetization relative to the relatively low value obtained at X-band. The most common higher EPR frequency that is still accessible with electromagnets is Q-band, around 34 GHz microwave frequency. With minimal exceptions, beyond Q-band, higher frequency EPR has utilized superconducting magnets, starting with W-band (around 94 GHz frequency). The initial modern-era W-band instruments were low power continuous-wave only^2,3^. Pulse EPR techniques have been implemented in subsequent instruments. Initially these employed only modest power in the hundreds of milliwatt range, but these instruments take advantage of high Q (quality factor) resonant cavities, including “homebuilt” instruments^4–7^ as well as an improved Bruker W-band instrument^8^ that followed their first lower power W-band that debuted in 1996. More recently, much higher power W-band pulse EPR instruments employing 1 kW level extended interaction klystrons were developed and employed for diverse applications^9,10^.

Reviews such as^11,12^ cover this interesting history of high field EPR development in greater detail. Fig. 1 shows the magnetization (vertical red line) obtained for this popular W-band frequency, for 3.4 T and a temperature of 4.2 K. The intrinsic magnetization is appreciably higher than for X-band but still falls far short of the maximum magnetization that could be achieved at yet higher frequency/field. In contrast, the rightmost (blue) vertical line illustrates the magnetization at the 9.4 T field for a g = 2 signal at 263 GHz and at a temperature of 4.2 K. Under these conditions, the paramagnetic system is near magnetic saturation, corresponding to most of the electron spins residing in the ground state provided by the electronic Zeeman interaction.

There have been many demonstrations of higher frequency ( > 94 GHz) EPR spectroscopy using continuous wave (CW) excitation or low power pulse applications, including^13^ (a very early 150 GHz work) and many others^14–21^. Indeed, we have made good use of our own low-power 130 GHz pulse EPR^16,22,23^. It is also worth noting that Bitter electromagnets have typically been employed at the very highest EPR fields and frequencies^20,24^.

Here we show the design, construction, and performance of a versatile high-power 263 GHz pulse EPR spectrometer incorporating a new 263 GHz “folded waveguide” TWT amplifier with over 10 W output power over a bandwidth of 5.6 GHz^25^. As noted above, an alternative approach to the use of high-power pulse amplifiers is to employ very small high Q resonators along with the modest powers ( ≈ 100 mW) available from solid state devices at such frequencies. The inevitable issue in scaling to frequencies well above W-band is that the resonant cavity dimensions scale inversely with frequency, resulting in a 1/*f*^3^ dependence of cavity volume with frequency, therefore resulting in very small, low volume EPR sample capillary tubes at these higher frequencies. For example, there is a commercial 263 GHz system available (Bruker Elexsys E780) with a reported *π*/2 pulse length of 30 ns with 100 mW power, employing a small resonant cavity with a sample volume of ~30 nL^26^. In contrast, at X-band and Q-band frequencies, pulse EPR spectrometers are often driven with high power (typically 1 kW for X-band) high bandwidth traveling wave tube (TWT) amplifiers. These are “slow-wave” vacuum electronics devices in which high voltage electron beam kinetic energy is transferred to a propagating microwave signal, resulting in RF amplification to high powers with the added benefit of intrinsically high spectral bandwidth. TWT fabrication at 263 GHz is challenging due to the very small and precise internal dimensions required since the microwave wavelength is a factor of  ≈ 30 smaller than for an X-band amplifier. Our new TWT now powers this new lab-built 263 GHz spectrometer (Supplementary Information Section 1.1). As shown herein, this TWT amplifier provides sufficient power to enable sensitive pulse EPR spectroscopy without the need for a resonator, providing relatively large sample volume, flexible sample geometries, and a high overall system bandwidth that accommodates the intrinsic high bandwidth of the TWT amplifier. This instrument offers large improvements in both frequency and bandwidth relative to the high power 95 GHz pulse EPR instruments previously noted^9,10,27^. Paramagnetic centers with spin *S*  > 1/2 present zero-field splitting (ZFS) interactions in addition to the electron spin Zeeman interaction, and at low EPR resonant frequencies, these interactions between multiple electrons can greatly broaden EPR signals. For these types of samples, an additional advantage of high frequency EPR is to lessen the relative effect of the ZFS by increasing the electronic Zeeman interaction vs. that in lower frequency spectrometers^28^. To characterize the performance of the new 263 GHz pulse EPR spectrometer, we begin with a study of a robust test sample, a commercial MgO solid sample which contains trace amounts of transition metals, including EPR-detectable high spin (*S* = 5/2) Mn(II)^29^. This allows us to compare signal intensity and resultant S/N vs. a widely-available commercial high power X-band pulse EPR spectrometer and to test the resolution enhancement this instrument may offer for multiple high spin paramagnetic metal centers found in this test sample. This work in turn serves as a basis for characterizing 263 GHz pulse EPR spectra of H.S. Mn(II) ions in frozen aqueous solution, including Mn(II) bound by two interesting metal sequestering proteins.

## Results

### 10 W 263 GHz pulse EPR performance

**Fig. 2:**
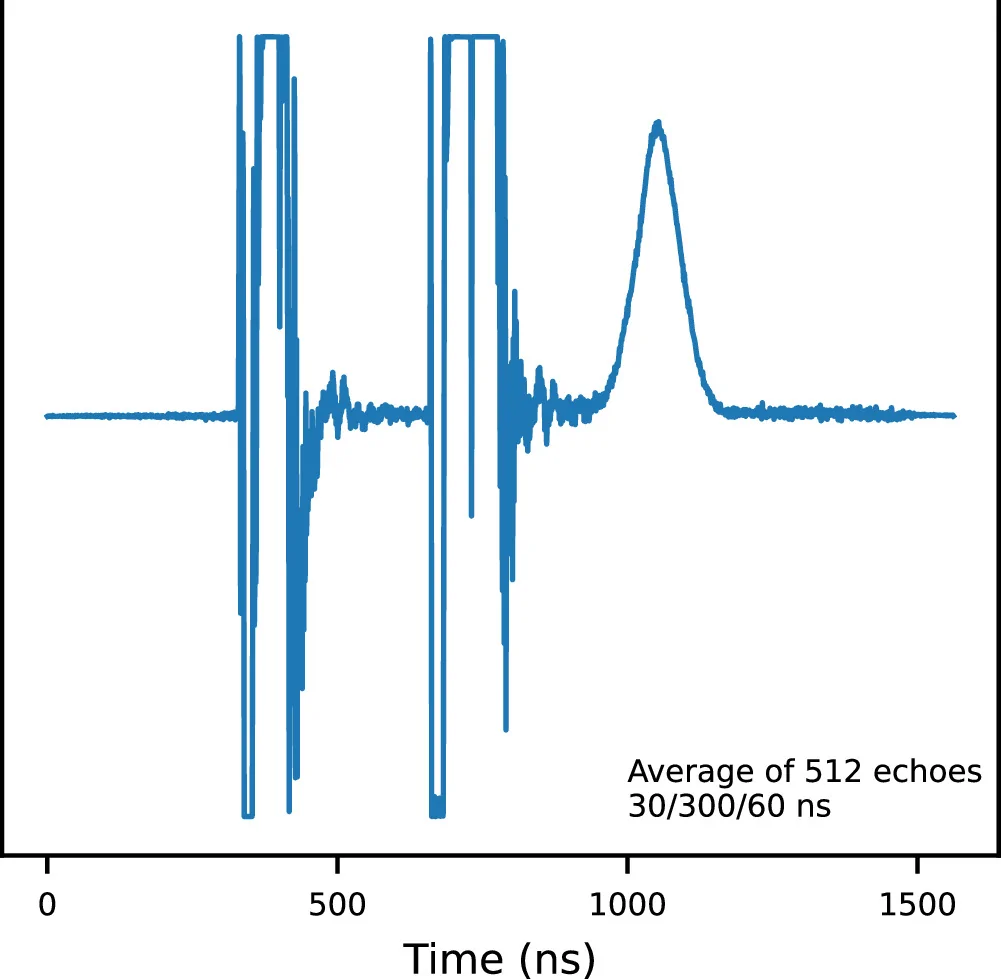
2-pulse Hahn echo of trace Mn(II) in the MgO sample. *π*/2 − *τ* − *π* times are 30 ns, 300 ns, 60 ns. Displayed echo is the average of 512 echos obtained with 1 kHz repetition rate. Temperature 100 K.

Figure 2 shows the 2-pulse Hahn echo at a field resonant with one of the six Mn(II) peaks (*vide infra*) observed in the nominal MgO sample, using the 10 W TWT without a resonator at a sample temperature of 100 K. The 10 W TWT was employed at full power, and a very strong echo was observed with a *π*/2 pulse duration of 30 ns. (Supplementary Information Section 1.2) The echo signal was quite easy to detect and optimize in this high bandwidth instrument, since there is no need to fine-tune the spectrometer frequency to match the frequency profile of a resonator. A Rabi oscillation analysis for a narrow line *S* = 1/2 “trityl” radical sample informed the choice of excitation pulse widths (Supplementary Information Section 3.1). We also observed some excitation pulse bleedthrough despite significant ( ≈ 35 dB) receiver isolation by the inductive coupling operating mode of the 263 GHz quasioptical bridge.

### 263 GHz Mn(II) spectrum and the sensitivity of the high-power resonator-free approach to mm-wave pulse EPR spectroscopy

**Fig. 3:**
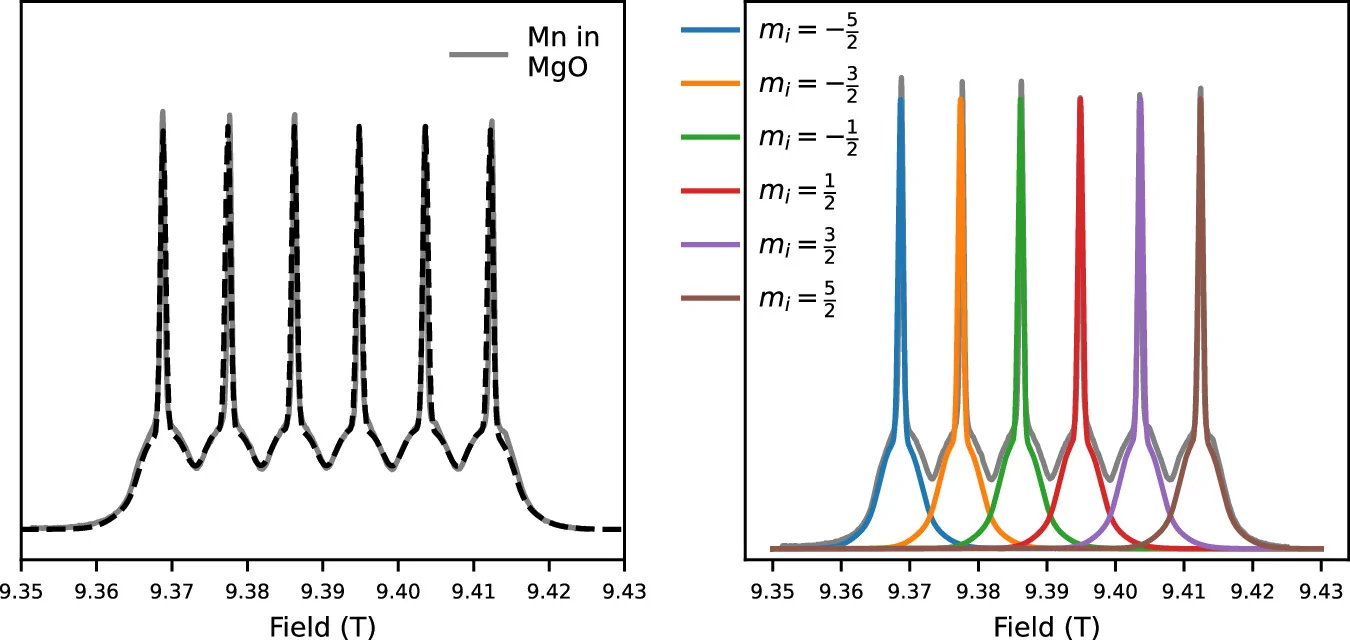
Left Panel: 263 GHz 2-pulse Hahn echo detected field swept spectrum of H.S. Mn(II) in the MgO sample. *π*/2 − *τ* − *π* times are 30ns, 300ns, 60ns. Each data point is the average of 512 echos obtained with 1 kHz repetition rate. Temperature 100 K. The dashed line is the simulation. Right Panel: the simulation broken down to the six individual m_*i*_ states for the I = 5/2 ^55^Mn nucleus, as split by the hyperfine interaction. Simulation details: *g *= 2.001, *A *= 243.6 MHz, *D *= −50 MHz, *E *= 0.

Figure 3 shows the field swept 263 GHz H.S. Mn(II) pulse EPR spectrum in the MgO test sample, as well as a spectral simulation. The * I *= 5/2 ^55^Mn nucleus is 100% naturally abundant, which through the hyperfine interaction gives rise to the 6-line spectrum observed. There are 30 fully allowed EPR transitions (*ΔM*_*S*_ = ± 1, *ΔM*_*I*_ = 0) for this spin system (Supplementary Information Fig. 18).^29–31^ Moreover, at 263 GHz the Zeeman splitting is so large that to thermally populate all six *M*_*S*_ states in order to observe all allowed EPR transitions, we performed our initial EPR experiments at the relatively high cryogenic temperature of 100 K (Supplementary Information Fig. 19). The six sharp features correspond to the *M*_*S*_ = − 1/2 ↔ *M*_*S*_ = 1/2 “inner transitions” that are broadened only to 2nd and higher orders in a perturbation treatment of the ZFS starting from pure Zeeman eigenstates^30,31^. These peaks are quite narrow at 263 GHz due to the very large intrinsic Zeeman interaction provided by this instrument (Supplementary Information Section 3.2). In contrast, the four remaining “outer transitions” are broadened to first order by ZFS interactions, resulting in larger linewidths. These transitions manifest themselves with a near-Gaussian lineshape, with some overlap between adjacent peaks in the 6-line pattern. Also, the semi-allowed transitions often observed at X-band are completely eliminated at 263 GHz with the dominance of the electronic Zeeman interaction over the ZFS interaction. In summary, the demonstrated sensitivity in our new high power 263 GHz instrument, operated without a resonator, in detecting the trace amount of H.S. S = 5/2 Mn(II) in the MgO sample is very high, with negligible noise at 100 K, even with only 0.512 s signal averaging per field value.

A comparison of the Mn(II) spectrum with and without the 10 W TWT amplification is displayed in Supplementary Information Section 3.4. The pulse EPR spectrum is  ≈ 9 times more intense with the TWT, as expected, given the much greater power afforded by the TWT (see discussion in Supplementary Information Section 3.4)

**Fig. 4:**
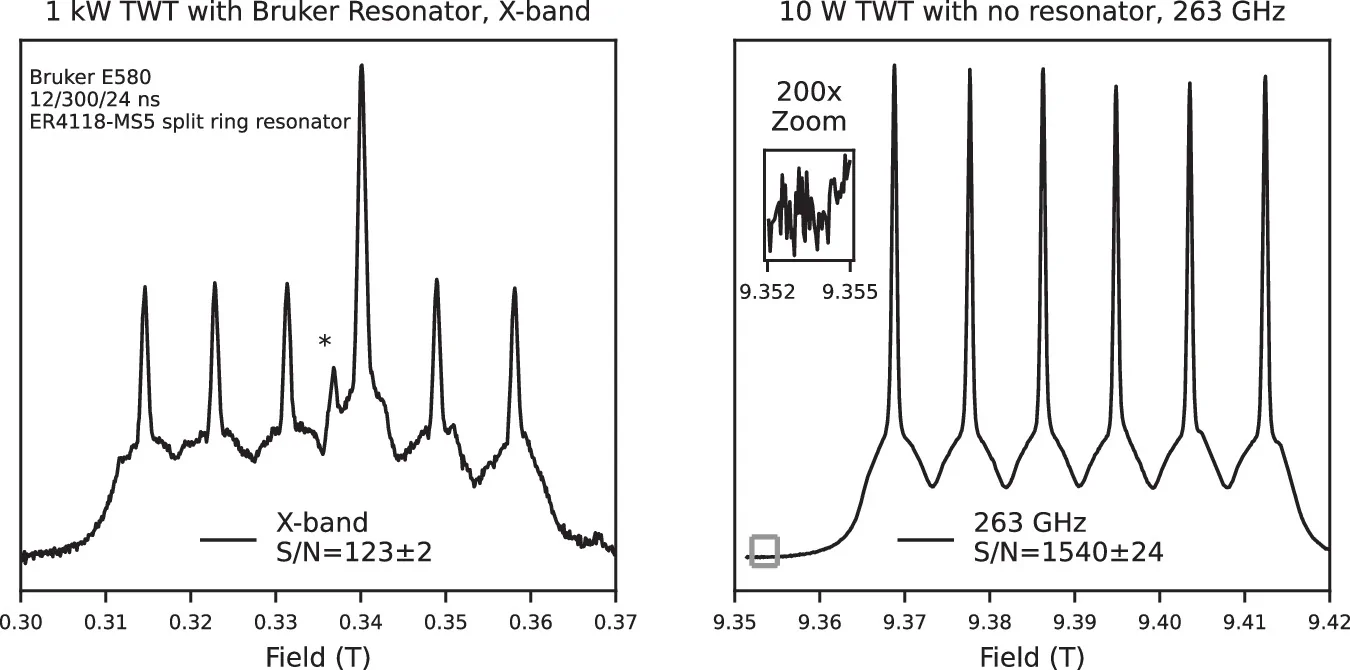
A comparison of X-band vs 263 GHz EPR spectra of the MgO powder in the Mn(II) spectral region. The X-band spectrum was taken with the UC Davis Bruker E580 pulse EPR spectrometer with a 1 kW TWT and a standard Bruker resonator. A sharp quartz radical signal is marked by *. The 263 GHz spectrum is from Fig. 3, but with a baseline region multiplied by x200 to get an accurate S/N estimate. The fourth peak with higher intensity is due to a Cr(III) signal that overlaps with the fourth Mn(II) central transition (Supplementary Information Fig. 25). Both spectra were obtained at 100 K with the identical sample mass (20 mg).

Figure 4 provides a direct comparison of the signal-to-noise obtained for this Mn(II) sample between the widely available Bruker pulsed X-band spectrometer and our 263 GHz instrument. The 263 GHz spectrum in the right panel is that of Fig. 3, but with an inset showing a baseline section multiplied by a factor of 200 to allow for an accurate noise estimate, resulting in a measured S/N of  ≈ 1540. The X-band spectrum in the left panel was obtained with our Bruker E580 pulse EPR with a standard Bruker resonator (ER4118-MS5). We measured a S/N ratio at X-band of  ≈ 123. Both sets of spectra were obtained with 20 mg of the MgO powder, with 512 echoes averaged at 1 kHz rep rate for each field value, and at 100 K temperature. Even without a resonator, the S/N ratio of the 263 GHz spectrum obtained for Mn(II) is enhanced by a factor of 12.5 over that of the X-band spectrum, demonstrating that the resonator-free approach for this mm-wave spectrometer performs quite well.

### The spectral resolution of the 263 GHz spectrometer for paramagnetic signals from trace elements in the MgO sample

**Fig. 5:**
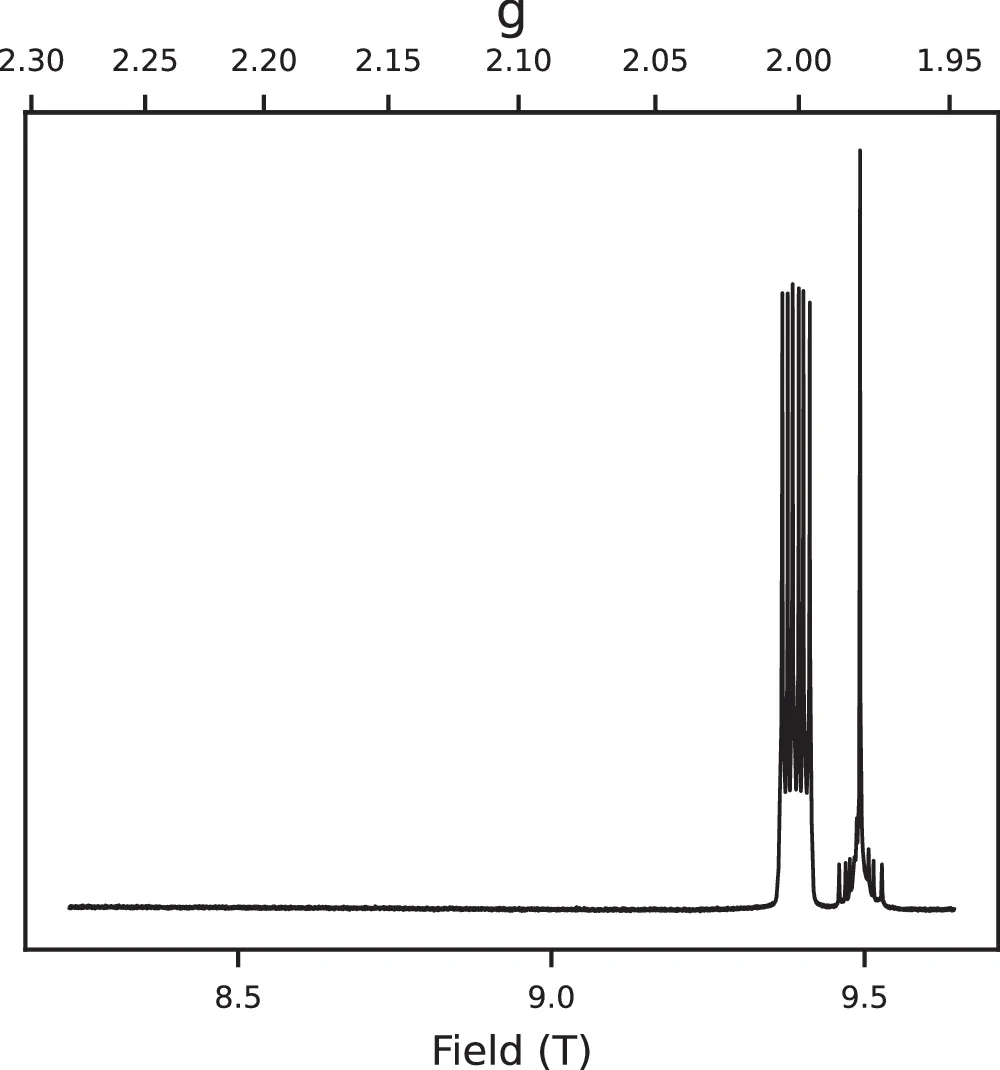
A wider 263 GHz field sweep of the MgO powder sample showing additional strong signals to higher field of Mn(II). The displayed spectrum is the average of 512 echos obtained with 1 kHz repetition rate. Temperature 100 K.

A broader field sweep of the MgO sample (Fig. 5), both above and below the Mn(II) spectral range, reveals that other strong EPR features are apparent upfield of the Mn(II) hexaplet signal. There is a strong single-line signal centered around *g *= 1.98. This overlaps with a signal with eight hyperfine lines at about the same *g*-value. This spectrum showcases the superb spectral resolution and clean baselines of the 263 GHz pulse instrument, which provides us the opportunity to clearly assign the spin states for these higher field signals via their Rabi oscillation patterns and to make a preliminary assignment as to their origins (Supplementary Information Section 3.6) as supported by an ICPMS analysis of the transition metal content of the nominal MgO sample (Supplementary Information Table 1). The strong sharp signal (labeled C in Supplementary Information Fig. 23) can be assigned to *S *= 3/2, and given the lack of hyperfine splitting and the large intensity, and the observed g = 1.98 value vs. literature reports^32^, we tentatively assign this signal to Cr(III), which is also consistent with the relatively high Cr concentration (16.387 ng Cr/mg MgO) from the ICPMS assay. The 8 line spectrum (denoted B in this figure) at the same g = 1.98 value, also assigned to *S *= 3/2, in principle could arise from *I *= 7/2 ^51^V of V(II) or ^59^Co of Co(II), but comparison of the *g* value is more consistent with literature reports for V(II)^33^, and moreover V was found to be present in the MgO sample as ~ten times greater concentration than Co (2.131 ng V/mg MgO vs. 0.192 ng Co/mg MgO).

In addition to the X-band spectra shown in comparison to the resonator-free 263 GHz spectrometer, Supplementary Information Sections 3.5 and 3.6 display spectra from this nominal MgO test from a recent vintage Q-band (34 GHz) pulse spectrometer.

### H.S. Mn(II) spectra obtained from frozen aqueous solution samples

**Fig. 6:**
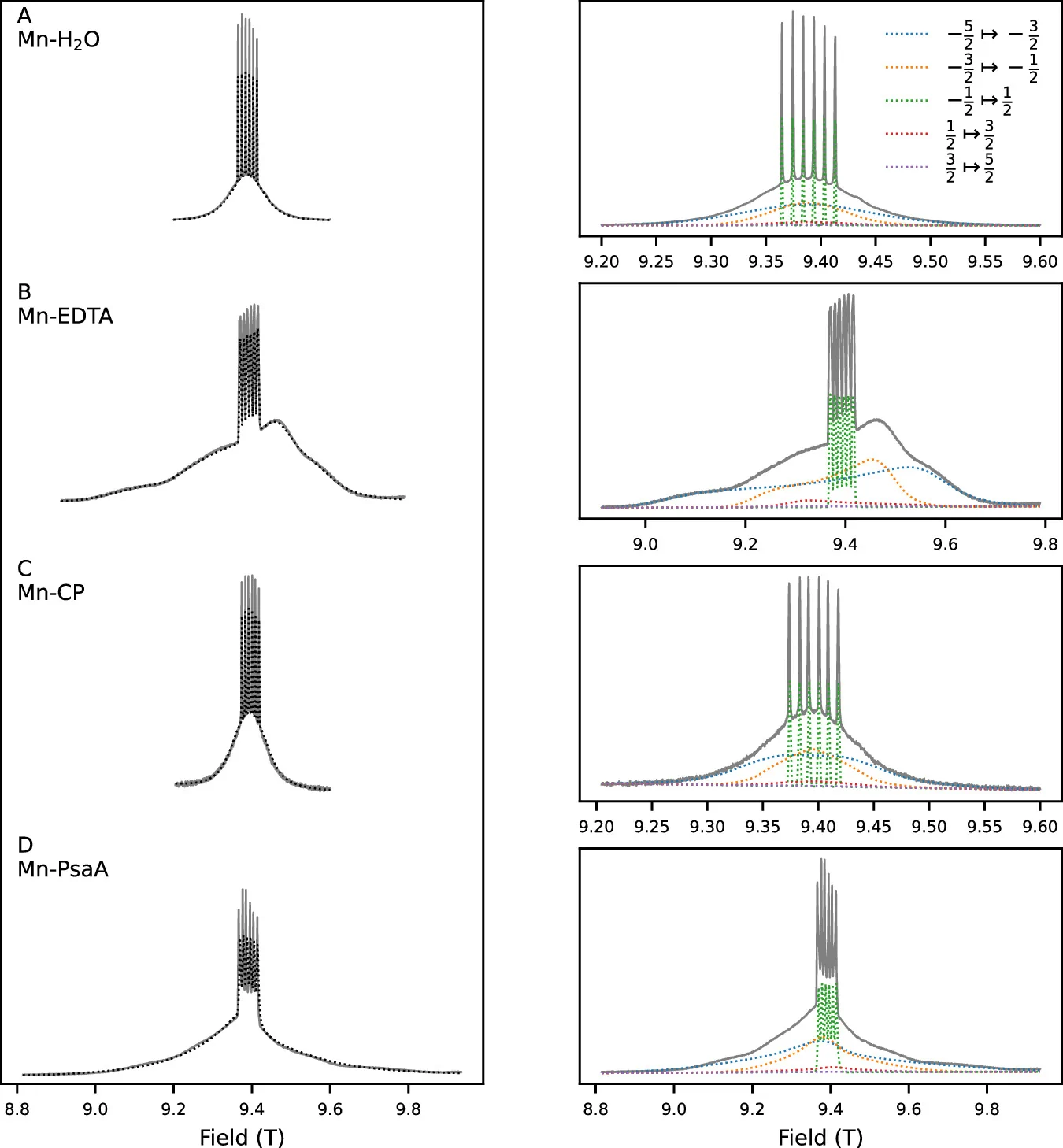
263 GHz Pulse EPR spectra of high spin Mn(II) complexes in frozen solution. Row A: (Mn-H_2_O): Left Panel: 2-pulse echo spectrum of Mn(II) in water. Temperature 10 K. The dashed line is the simulation. Right Panel: the simulation broken down to show the five sets of allowed EPR transitions for the spin *S *= 5/2 H.S. Mn(II) ion. Row B: (Mn-EDTA): Left Panel: 2-pulse echo spectrum of a Mn(II)-EDTA complex in water. Temperature 10 K. The dashed line is the simulation. Right Panel: the simulation showing each of the five sets of allowed EPR transitions. Row C: (Mn-CP): Left Panel: 2-pulse echo spectrum of a Mn(II) bound in the metal sequestering protein calprotectin. Temperature 10 K. The dashed line is the simulation. Right Panel: the simulation showing each of the five sets of allowed EPR transitions. Row D:(Mn-PsaA): Left Panel: 2-pulse echo spectrum of a Mn(II) bound in the metal sequestering protein PsaA. Temperature 10 K. The dashed line is the simulation. Right Panel: the simulation showing each of the five sets of allowed EPR transitions. The pulse EPR operation is described in Supplementary Information Section 1.2.

Our primary focus with this new high frequency pulse EPR spectrometer is in the broad areas of bioinorganic chemistry and metal based catalysis in solution, so it is crucial to characterize the performance of the instrument for high spin metal ions in solution as well as incorporated into metalloproteins. Figure 6 (Row A: Mn-H_2_O) shows the 263 GHz 2-pulse echo field sweep of H.S. Mn(II) in a buffered water/cryoprotectant frozen solution. The spectrum (left panel) is qualitatively different from Mn(II) in MgO solid, in that there are 6 sharp hyperfine lines arising in the center of a single broad Gaussian line. Decomposition of the simulation into the five sets of EPR transitions (right panel) shows that only the *M*_*S*_ = − 1/2 ↔ *M*_*S*_ = 1/2 transitions show resolved ^55^Mn hyperfine splitting, and due to larger ZFS interactions the outer transitions are much broader than in the MgO sample, and hence overlap significantly so that they collectively contribute to the single observed broad underlying feature.

The ZFS interaction for a molecular complex of Mn(II) with EDTA in frozen aqueous solution is in turn much larger than for Mn(II) alone, as shown in Fig. 6 (row B: Mn-EDTA). Here one observes asymmetric outer transitions with some resolution of the turning points in the frozen powder pattern, all together summing to a less symmetric lineshape compared to Mn(II) without this strongly chelating ligand^31^.

Figure 6 (Row C: Mn-CP), left panel, presents the 263 GHz spectrum of Mn(II) in the metal sequestering protein calprotectin, which binds this high spin ion in a highly symmetric binding site with ligation by six histidine residues^34,35^. This Mn(II)-His6 site was found to have a relatively small ZFS interaction in prior X-band CW and pulse experiments and high field CW EPR experiments^34,36^. This results in a 263 GHz spectrum similar to Mn(II) in water, but with an even narrower Gaussian lineshape arising from the four outer electron spin transitions because of the reduced ZFS arising from this very high symmetry protein environment. Calprotectin outcompetes for Mn(II) with some other Mn-binding proteins such as streptococcal PsaA, which binds Mn(II) in a lower symmetry site with a resultant larger ZFS interaction^36^. 263 GHz pulse EPR of Mn(II) in PsaA shows spectral changes induced by this larger ZFS interaction (Fig. 6, Row D: Mn-PsaA) with some resolution of the ZFS features appearing for the four outer spin transitions.

## Discussion

The results presented here show that 10 W 263 GHz pulses from the recently fabricated TWT amplifier are adequate to perform high-sensitivity and high-bandwidth pulse EPR spectroscopy in our new mm-wave pulse spectrometer, even without the use of a high Q resonator. Importantly, this 10 W pulse power level is also not so high as to damage the receiver system, so this seems to be an ideal amplifier power for mm-wave pulse EPR spectroscopy.

Additionally, employing this high frequency spectrometer in resonator-free mode offers numerous advantages:

- Much larger sample volume than available with a spectrometer designed with a high Q resonator at this high mm-wave frequency. The data displayed here employed Teflon sample cups with ~12 μL sample volume vs. about 30 nL in the capillary tubes used in the commercial 263 GHz EPR with its small volume resonator^26^. Thus, the instrument can sample many more spins than available in the alternative low power/small resonator implementation, while still using rather modest sample volumes which can be crucial for some biochemical samples^37^, for example with expensive magnetic isotope labels which are often crucial for pulsed EPR experiments.
- Ease of tuning and high bandwidth operation, including advantages for shaped and chirped AWG generated pulses, and electron-electron double resonance experiments that will not be limited in difference excitation frequencies by the relatively small bandwidth of a high Q resonator.
- The open 5 mm diameter sample geometry will allow for a variety of specialized applications, such as study of 2-D samples, devices that cannot be placed in tiny EPR tubes, straightforward in situ electrochemistry, and flash generation of light-induced paramagnetic signals obtained with a thin flat disk geometry. Moreover, we can directly flash-freeze reaction intermediates into a shallow recess in the gold plated probe bottom at liquid nitrogen temperature and immediately employ for pulse EPR experiments without removing the frozen sample “snow” and transferring/packing into tiny EPR tubes.
- Roomy access for RF coils for electron-nuclear double resonance (ENDOR) or dynamic nuclear polarization (DNP) without concern of “Q-spoiling”.
- The combination of the frequency agile TWT amplifier and the arbitrary waveform generator will make this instrument an effective platform for testing time domain DNP methods at a respectably high NMR frequency (400 MHz for protons, which is the reason we specifically targeted 263 GHz in the instrument design).

We observe three distinct pulse EPR signals in the MgO standard sample arising from high spin metal ions (Fig. 5) (Supplementary Information Section 3.6) over the tested field range. As demonstrated here, such H.S. ions are particularly good targets for such high-field pulse EPR since the central transitions become relatively narrow in the regime where the electronic Zeeman interaction dominates the ZFS interaction. This effect is directly analogous to the improvement in sensitivity and resolution for quadrupolar nuclei afforded by very high field NMR spectroscopy^38^.

We demonstrate the utility of 263 GHz pulse EPR of H.S. Mn(II) in varied environments, from that as a trace component in an MgO solid powder (Fig. 3) to Mn(II) in simple aqueous solution, in a coordination complex with EDTA, and bound in metal sequestration sites in two different proteins (CP and PsaA) (Fig. 6). In all cases the observed 6-line spectrum, arising from the ^55^Mn hyperfine interaction with the *M*_*S*_ ± 1/2 electron spin levels, is strong and well resolved. This will provide an excellent basis for hyperfine-targeting pulse EPR experiments such as ENDOR and EDNMR in these and other Mn(II) binding proteins. In addition, the ZFS interactions at such high fields act as perturbations upon the stronger electron Zeeman interactions, so we measure the ZFS effects on the outer Mn(II) spin transitions with high sensitivity and precision (Table 1), and ENDOR/EDNMR performed at these fields will provide “orientation selectivity” with additional information about the hyperfine tensors vs. ZFS tensors (Supplementary Information Section 3.2)^31^. The ^55^Mn hyperfine interaction is not resolved in these broader features, but we anticipate being able to measure this with these hyperfine-focused pulse sequences. Additionally, for some Mn(II) binding proteins, dipolar spectroscopies such as DEER will provide interspin distances and distance distributions. And the high sensitivity and resolution shown here for H.S. Mn(II) will translate to other H.S. ions where the high Zeeman frequency in our new pulse instrument will provide superior EPR interrogation, not only d-block transition metals but also f-block ions which have been targeted with renewed focus in diverse areas such metalloprotein binding, quantum information, and organometallic catalysis in recent years^27,39,40^.

**Table 1:** Mn(II) Zero-Field Splitting Parameters, Confidence Intervals (C.I.), and Prior Measurements

Mn(II) Sample	*D*(GHz)*	∣ *E* ∣	∣ *E/D* ∣	*D* 95% C.I.	*E* 95% C.I.	Ref
Mn(H_2_O)	0.657	0.186	0.28	0.651–0.664	0.179–0.193	this work
	0.550	0.055	0.1			^59^
	0.430	0.140	0.32			^60^
	0.610	0	0			^16^
Mn-EDTA	–2.534	0.088	0.04	−2.539 - –2.529	0.068–0.108	this work
	–2.55	0.255	0.1			^61^
	1.5	0.5	0.3			^62^
	3	0.3	0.1			^16^
Mn-CP	0.548	0.128	0.23	0.539–0.558	0.112–0.144	this work
	0.485	0.146	0.3			^34^
	0.525	0.158	0.3			^35^
Mn-PSA	+2.749	0.667	0.24	2.607–2.890	0.638–0697	this work
	+2.87	0.35	0.122			^36^

*Determination of the sign of *D* is based on the asymmetry of the spectrum (Supplementary Information Section 3.3). In the table, if there is no + or - sign, the actual sign of *D* could not be conclusively determined

In order to accentuate the sharp central transitions for Mn(II) which originate from thermally excited spin manifolds, the spectra shown here were obtained at relatively high cryogenic temperatures, well above 4.2 K or the pumped He temperatures that can be obtainable in our sample cryostat. At readily accessable lower temperatures on our setup (tested to 1.37 K), and at the high field of our experiments in this 263 GHz instrument, pulse EPR sequences will begin with thermal equilibrium providing nearly pure spin ground states and near unity magnetic polarization (Fig. 1, see Supplementary Information Section 3.3 for details). This is a rare situation for conventional magnetic resonance, allowing this new 263 GHz pulsed EPR instrument to be extremely useful in the fields of molecular magnetism and quantum science as targeted by EPR spectroscopy^41–44^, including characterizing the important role of ZFSs in molecular magnetism of Mn compounds^45^.

The Mn(II) signal-to-noise obtained in the 10 W resonator-free 263 GHz pulse instrument is appreciably higher than for the Bruker E-580 X-band pulse EPR spectrometer. This indicates the instrument will have the required sensitivity for important pulse EPR experiments such as ENDOR^31,46^, ELDOR detected NMR (EDNMR)^47,48^, and double electron electron resonance (DEER)^49,50^.

Although the high frequency line narrowing for the *m*_*s*_ = − 1/2 ⇆ *m*_*s*_ = + 1/2 transitions is particularly useful for high spin ions, the high field pulse EPR will also be very useful for S = 1/2 radical species with modest g-resolution that are often not well resolved in low field experiments, limiting the power of orientation selection in high resolution pulse experiments. The other advantages noted above will still be in place. We do note that for S=1/2 samples with well resolved g-values at low field, such as Cu(II) or S = 1/2 states of Fe-S clusters, there will be a downside of spreading the spectra over field values well in excess of what is needed for proper g-resolution. Lower field instruments may indeed be better for many experiments in such systems, but it will be good to evaluate a few test samples to see how the competing factors trade off in practice. The other advantages of the resonator-free system listed above will still hold, including eliminating the loading of small Q-band EPR tubes with RFQ frozen powders.

Finally, as briefly noted earlier, with the addition of NMR electronics and RF coil components, this 263 GHz pulse EPR spectrometer promises to be a useful testbed for exploring time domain dynamic nuclear polarization methods for transferring the very high electron spin polarization at these high frequencies and fields to nuclear spins in order to achieve large sensitivity gains in NMR spectroscopy, and at a reasonably high NMR frequency (400 MHz for protons)^51–54^. The fact that the TWT is a high bandwidth pulse *amplifier*, not an oscillator *(*a source) like a gyrotron or free electron laser^55^, will provide much versatility for such electron—nuclear polarization transfer experiments, particularly given the huge electron polarization achieved in this instrument as well as the wide ranging pulse versatility that will be accessible with this broadband spectrometer using an arbitrary waveform generator as an input source^56^. We also note that the instrument itself accurately measures the 263 GHz pulse EPR spectra and electron spin relaxation times, something not generally possible with gyrotron-based DNP instruments.

## Methods

### MgO sample

The nominal 95% pure fused MgO was purchased from Sigma: HR23225JC.

### ICP-MS

#### Chemicals

Concentrated Trace Metal Grade (TMG) nitric acid (A509P500) and single-element ICP-MS calibration standards for V, Cr, Mn, Fe, Co, Ni, Cu, and Zn were purchased from Fisher Scientific (Waltham, MA, USA). Single-element internal standard solutions containing Sc, Ge, Y, In, and Bi were obtained from Inorganic Ventures (Christiansburg, VA, USA). NIST 1643f Trace Elements in Water certified reference standard was acquired from the National Institute of Standards and Technology (Gaithersburg, MD, USA). All solutions were prepared using 18.2 M*Ω*/cm MilliQ Water (MQW) water (Direct-Q 3, Millipore, Burlington, MA, USA).

#### Sample preparation for ICP-MS analysis

Commercial 95% MgO powder (7.4 mg) was digested in 1.5 mL concentrated Trace Metal Grade (TMG) HNO_3_ (A509P500, Fisher Scientific) at 100 ^∘^C for 2–4 h in loosely capped vessels using a heat block. The digest was then diluted 10-fold by adding 300 μL of the stock digest to 3.0 mL final volume with 18.2 M*Ω*/cm MQW and internal standard solution to achieve a final concentration of 5% concentrated TMG HNO_3_. A digestion process water blank was prepared following the same procedure without sample addition.

#### ICP-MS analysis

Digested MgO samples and digestion blanks were analyzed for V, Cr, Mn, Fe, Co, Ni, Cu, and Zn, which are common transition metal impurities in commercial metal oxide materials. Digested MgO samples and digestion blanks were analyzed using an Agilent 8900 ICP-MS Triple Quadrupole instrument (Agilent Technologies) at the Interdisciplinary Center for Plasma Mass Spectrometry, University of California, Davis. The instrument was equipped with Ni interface cones and an x-Lens, and operated in MS/MS He Mode using a 3-point peak pattern with three replicates per injection and 50 sweeps per replicate. Samples were introduced via an Agilent SPS 4 Autosampler with 0.5 mm ID sample probe using a 0.1 rps peristaltic pump and mixed at ~17:1 ratio with custom internal standard solution containing Sc, Ge, Y, In, and Bi using a mixing tee. A 400 μL/min Micro Mist nebulizer generated aerosol in a 2 ^∘^C temperature-controlled double pass Scott-type spray chamber leading to a 1550 W plasma. Helium was used as collision cell gas to manage polyatomic interferences. All measurements were performed using the most abundant isotope for each element (^51^V, ^52^Cr, ^55^Mn, ^56^Fe, ^59^Co, ^60^Ni, ^63^Cu, and ^66^Zn).

#### Calibration and quality control

Single-element calibration standards for V, Cr, Mn, Fe, Co, Ni, Cu, and Zn were prepared from certified reference materials (Inorganic Ventures) in 5% concentrated TMG HNO_3_. Seven-point calibration curves ranging from 5 to 1000 ppb were established using Ge as internal standard. Quality assurance included analysis of digestion process blanks, NIST 1643f Trace Elements in Water standard, and quality control standards analyzed before and after every ten sample injections to monitor instrument drift and verify calibration. Raw data were processed using MassHunter 4.6 ICP-MS software (G7201C, Version C.01.06, Agilent) to determine trace metal concentrations reported as ng metal/mg MgO powder.

### Mn(II) binding proteins

Buffers for experiments with CP-Ser and PsaA were prepared with ULTROL grade HEPES (EMD Millipore), Suprapur NaCl (EMD Millipore), and MQW water (18.2 M*Ω*cm, 0.22 μm filter). Emsure NaOH (Supelco) was used to adjust the buffer pH. Stock solutions of Ca(II) (1 M) and Mn(II) (1 M) were prepared in acid-washed volumetric glassware from ≥99.5% CaCl_2_2H_2_O (Sigma Aldrich) and ≥99.99% MnCl_2_4H_2_O (Sigma Aldrich), respectively, and Milli-Q water, and the solutions stored in polypropylene tubes. Working solutions of metal stocks were prepared by dilution of the 1 M stock solutions in Milli-Q water immediately prior to each experiment. The apo CP-Ser heterodimer [S100A8(C42S)/S100A9(C3S)], was overexpressed, purified, and stored as described previously^57^. CP-Ser concentration was determined using the calculated extinction coefficient for the S100A8(C42S)/S100A9(C3S) heterodimer (*ϵ*_280_ = 18,450 M^−1^cm^−1^, calculated using the ProParam tool available at http://web.expasy.org/protparam/). The protein PsaA was overexpressed, purified, demetalated, and stored as described previously^58^. PsaA concentration was determined using the calculated extinction coefficient (*ϵ*_280_ = 35,870 M^−1^cm^−1^, calculated using the ProParam tool). Protein samples were thawed on ice and buffer-exchanged into 75 mM HEPES, 100 mM NaCl, pH 7.5 using 0.5-mL 10K MWCO Amicon spin concentrators. CP-Ser was exchanged three times, PsaA was exchanged six times as previous experiments showed evidence of residual EDTA contamination from the protein purification in the protein samples when less thorough buffer exchange was performed^58^. To prepare Ca(II)- and Mn(II)-bound CP-Ser (heterotetramer), CP-Ser was concentrated to 1 mM (50 μL), and first incubated with 10 mM Ca(II) (5 μL from a 100 mM working solution) for 15 min. Subsequently, 750 μM Mn(II) was added to the sample (3.75 μL from 10 mM working solution), and incubated for 15 min before being aliquoted (10 μL) into polypropylene sample cups or PCR tubes and frozen in liquid nitrogen. To prepare Mn(II)-bound PsaA, protein samples were concentrated to 1 mM (50 μL), then incubated with 750 μM Mn(II) (3.75 μL from 10 mM working solution) or 375 μM Mn(II) (18.75 μL from 10 mM working solution), respectively, for 15 min. The samples were then aliquoted (10 μL) into polypropylene sample cups or PCR tubes and frozen in liquid nitrogen. In previous work, a comparison of the Mn(II) EPR spectra of Mn(II)-bound PsaA prepared with and without PEG-200 showed that the presence of PEG-200 affected the signal^58^. As a result, all studies were performed without PEG in the sample. The frozen samples were shipped to the University of California Davis overnight on dry ice for EPR spectroscopic analysis.

## Supplementary information

Supplementary Information

Transparent Peer Review file

## Source data

Source Data

## Data Availability

Data are available in the Source Data file. Source data are provided with this paper.
