# Supplementary material for: Very high frequency (263 GHz) pulse EPR spectroscopy of high spin transition metal centers
Source: Nat Commun. 2026 Oct 1;17:11275. doi: 10.1038/s41467-026-78076-w (PMC13720073; doi:10.1038/s41467-026-78076-w)
Supplement: Supplementary file 1 [file 41467_2026_78076_MOESM1_ESM.pdf]

Supplementary Material for: Very high frequency (263 GHz) pulse EPR spectroscopy of high-spin transition metal centers

Zikri Hasanbasri[1], Shasha Qiu[2], Guodong Rao[1], Amanda Caceres[1], Erica Debley[3], Yuan Zheng[2], Elizabeth M. Nolan[3], Marie Heffern[1], Paul Stucky[1*], Neville C. Luhmann, Jr.[2*], R. David Britt[1*]

[1]Department of Chemistry, University of California Davis, 1 Shields Ave, Davis, CA, 95616, USA.
[2]Department of Electrical and Computer Engineering, University of California Davis, 1 Shields Ave, Davis, CA, 95616, USA.
[3]Department of Chemistry, Massachusetts Institute of Technology, Cambridge MA, 02139, USA.

*Corresponding author(s). E-mail(s): pastucky@ucdavis.edu; ncluhmann@ucdavis.edu; rdbritt@ucdavis.edu;

1 Supplementary Methods

1.1 10 W 263 GHz EPR Spectrometer

Supplementary Tables 2 and 3 list the spectrometer components for the transmitter (TXMR) and receiver (RCVR), respectively, and are referenced by number in the spectrometer diagram of Supplementary Figure 1. The base frequency, f_o, of our spectrometer is 8.7667 GHz which is mixed and multiplied up to 263 GHz by the TXMR microwave electronics which includes a custom 200+ mW amplifier-multiplier chain (AMC) developed by Virginia Diodes, Inc. (VDI). The VDI AMC is the input driver for our custom 10 W pulsed traveling wave tube (TWT) amplifier. A portion of the base frequency is shunted to the RCVR via twenty feet of low-loss coaxial cable and forms the basis for the two local oscillators for the heterodyne double down conversion to baseband. The TXMR includes four selectable phase channels for perfoming phase cycling and unwanted echo cancellation. A second microwave channel is included for performing EDNMR-type experiments[1, 2]. A SpinCore Pulseblaster card is used to control the various microwave switches and the spectrometer control software is Specman4EPR[3] developed and maintained by Boris Epel, University of Chicago. A Cryogenics, Ltd., 12 T superconducting magnet working at 9.4 T (for a g=2 EPR signal) creates the main static field for our spectroscopy. This spectrometer topology is common in many high frequency EPR spectrometers[4, 5]. A highlight of the RCVR is another VDI AMC (MixAMC) which includes a subharmonic mixer for the first down conversion from 263 GHz to 8.7667 GHz. The second down conversion utilizes a Marki Microwave I/Q mixer where RF and LO frequencies are identical so that the baseband signal is the envelope detected 263 GHz waveforms that include EPR preparation pulses and ultimately electron spin echoes. The layout and dimensions of our spectrometer, especially the quasi-optical (QO) components and QO design parameters of the TXMR and RCVR plates and probehead, are derived from spectrometers developed by Takahashi[6] and Sherwin[7]. The main RCVR isolation from the high power TXMR preparation pulses is accomplished

via the polarization-based induction method[8]. Isolation via the induction method was measured to be better than 35 dB.

A CAD rendering of most of the spectrometer details is shown in Supplementary Figure 2. The TXMR assembly and in particular the TWT amplifier is placed about thirteen feet horizontally from the central bore of the magnet to reduce the potential effects of stray magnetic field on the electron optics of the TWT's electron beam in the slow wave structure of the tube. The stray field of the magnet as measured near the TWT was about 7 G when the main field was at 9.4 T. As an added precaution, the TWT was placed inside a large bore mu-metal shield. No significant effect on TWT operation is observed under these conditions. The 10 W EPR preparation pulses are conveyed from the WR3.4 fundamental waveguide output of the tube by a Gaussian corrugated horn with 18 mm ID aperture that is butted up against and coaxial with one end of lab-built helically corrugated waveguide[9] which is 12 ft in length and formed by connecting 1 ft sections. With the TWT amplifier placed at this distance, it permits relatively simple shielding from the stray field of the 12 T superconducting magnet via an open-ended tube of HyMU80 mu-metal, shown in Supplementary Figure 3. The estimated saturated output power at the TWT's output flange is 10 W peak pulse power. The peak pulse power that is radiated from the end of the long corrugated waveguide in the form of a TEM00 Gaussian beam is about 4.4 W of peak pulse power. Including measured loss of QO components and probehead, the estimated power delivered to the sample space is about 2.2 ± 0.2 W.

A schematic flow diagram of the Gaussian beam quasi-optical (QO) circuit is shown in Supplementary Figure 4. A combination of corrugated waveguide which supports the HE11 waveguide mode and free space sections which support a TEM00 Gaussian beam mode are employed to reduce attenuation and permit easier integration of QO elements such as wire grid polarizers and flat ferrite Faraday rotators.

Elements of the probehead are shown in Supplementary Figure 5. From left to right in the figure the probehead is shown in its vertical stand. The straight section is about 954 mm of 18 mm ID corrugated waveguide. The center panel shows the corrugated waveguide taper at the lower end of the probe for transitioning from 18 mm ID to the 5 mm ID sample section. Finally, the top of the probe with its anti-reflection coated quartz window and two vacuum ports is shown in the right panel. The top of the probehead and waveguide sections were fabricated by Thomas-Keating in the UK. The remaining outer superstructure with vertical nickel silver tubes and horizontal copper radiation baffles were lab-built.

The beam paths for the TXMR and RCVR plates are shown in Supplementary Figure 6. The figure shows the as-built optical breadboard plates with QO components such as ellipsoidal mirrors (fabricated at UC Davis College of Letters and Sciences machine shop), wire grid polarizers (made by Pure Wave Polarizers, UK) and a flat ferrite Faraday rotator on the TXMR plate (made by Thomas-Keating, UK). The ellipsoidal turning mirrors are designed with QO focal length of 254 mm [10, 6]. The parent ellipse parameters from which each mirror's surface was accordingly machined is shown in Supplementary Figure 7. The mirrors are 150 mm wide by 100 mm tall made from 6061 alloy aluminum. The machining parameters are the following: tool was a 4 flute carbide ball end mill, 0.75 in diameter, end mill finishing step over of 0.0075 in, 45 inch per minute feed rate at 7000 rpm and finished along the x (long) axis. The post machining polishing parameters are the following: wet sand lightly starting with 600 grit and working up to 2000 grit, which makes polishing easier. Finally, Osborn white rouge was employed with a cotton buff wheel along both x (long) and y axes (short) to bring the mirror surface to optical mirror quality which is not necessary, but is best when using a red or green laser for initial "sighting in" of the QO set up.

The physical arrangement of the spectrometer in the laboratory is shown in Supplementary Figures 8, 9, 10. The transmitter assembly with its TWT amplifier is set about thirteen feet away from the TXMR and RCVR plates to isolate the TWT amplifier from the stray field of the superconducting magnet. The mu-metal tube is shown open to reveal the TWT amplifier in Supplementary Figure 9. The height of the spectrometer was dictated by the magnet height in combination with the length of the probehead. The TXMR and RCVR plates can be opened and closed as shown in Supplementary Figures 11 and 12. The plates slide on linear bearings so that they can be opened to permit insertion or removal of the probehead. Upon closing the plates the TXMR and RCVR plate quasi-optics align with the vertical corrugated waveguide aperture of the probehead. Fine adjust of the position of each plate in three dimensions is built into the overall assembly.

The sample space at the bottom of the probehead is shown in Supplementary Figures 13 and 14. The probehead's corrugated waveguide tapers from 18 mm ID to 5 mm ID and the 5 mm ID section is about 50 mm long. The waveguide ends in a gold plated flat metal reflector upon which a small PTFE (i.e., Teflon) sample holder or "bucket" of 12 μL volume can be symmetrically placed using a bit of Corning vacuum grease to secure it, shown in Supplementary Figure 15. The gold reflector plate allows for simple sample handling for loading at cold temperatures. A simple setup that uses cold baths, such as liquid N_2, enables conditioning of the reflector plate to maintain the temperature of the sample that has already been flash-frozen prior to loading, depicted in Supplementary Figure 16. Hence, samples can be flash-frozen under conditions appropriate for the sample/protein of interest and carried over to the probehead while remaining frozen.

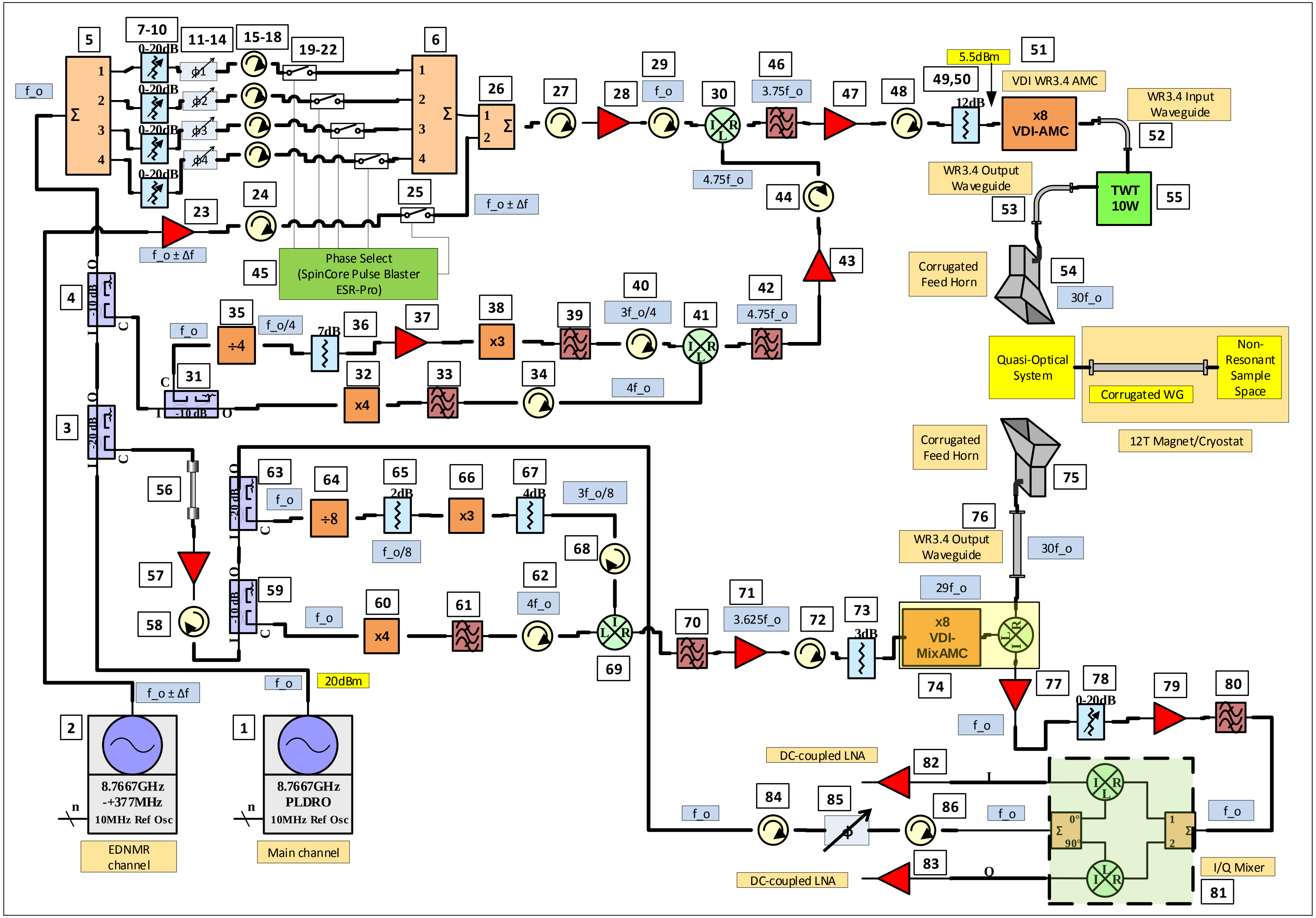

Supplementary Figure 1: 10 W 263 GHz pulse spectrometer block diagram.

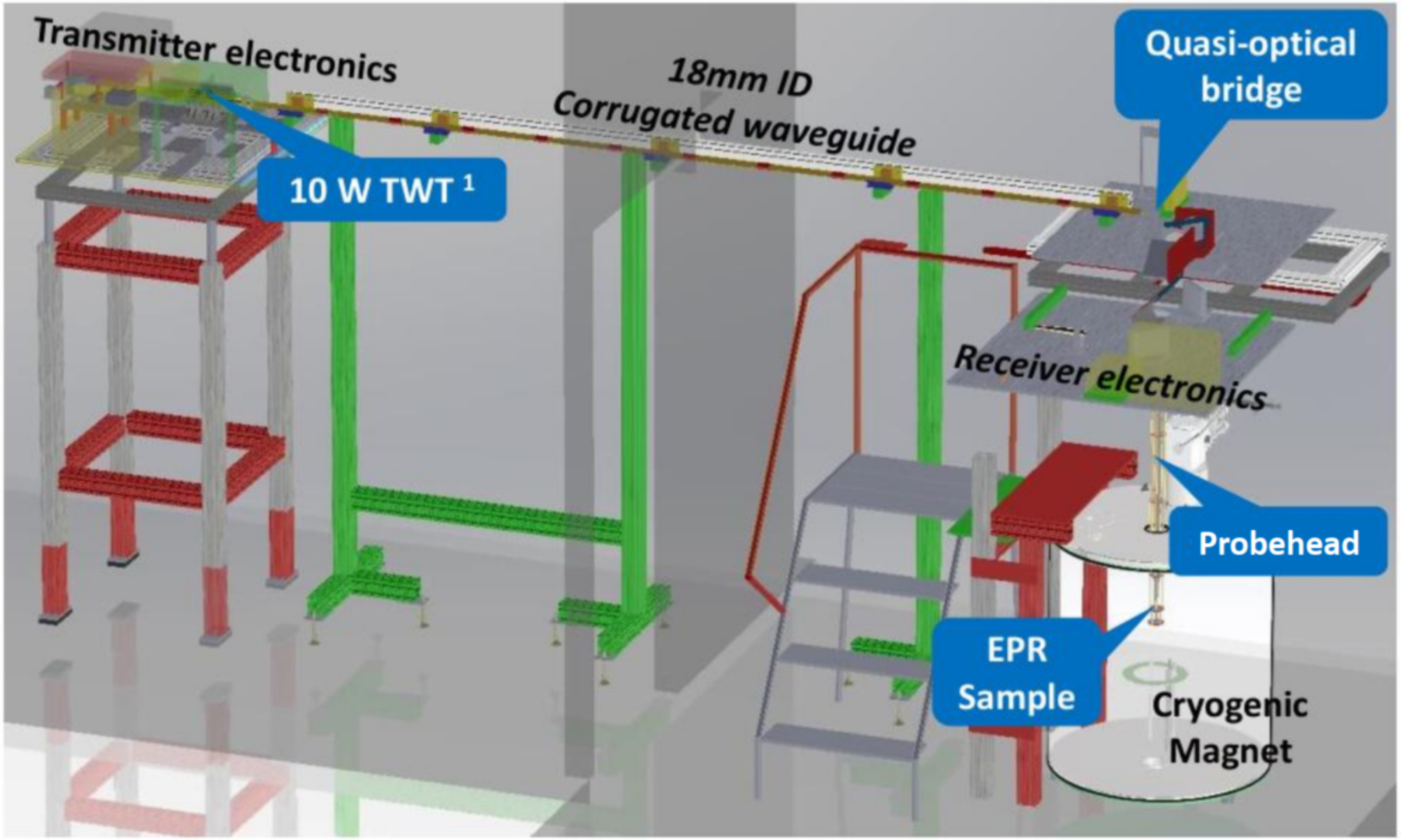

Supplementary Figure 2: 263 GHz quasi-optical pulse EPR spectrometer CAD Rendering.

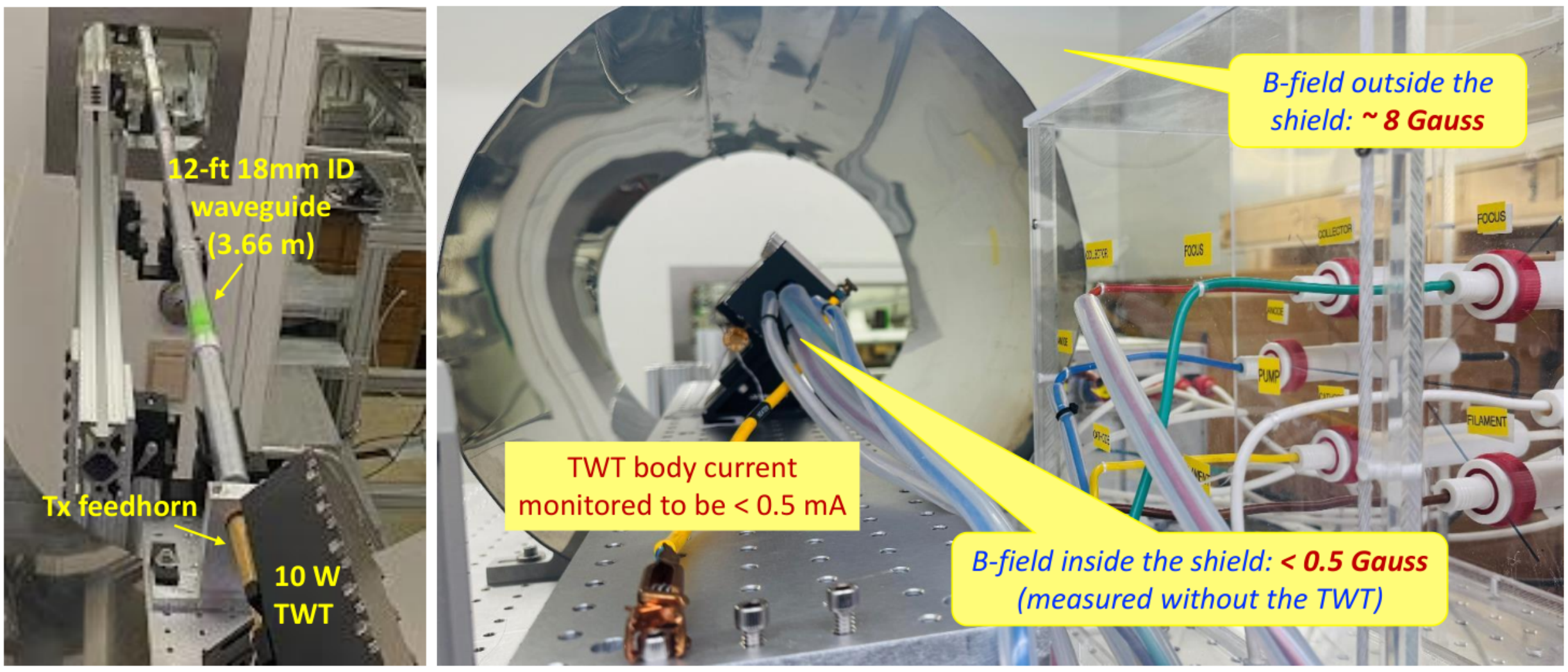

Supplementary Figure 3: View of the twelve foot long 18 mm ID helically corrugated waveguide and view from the rear of the TWT inside its mu-metal magnetic shield.

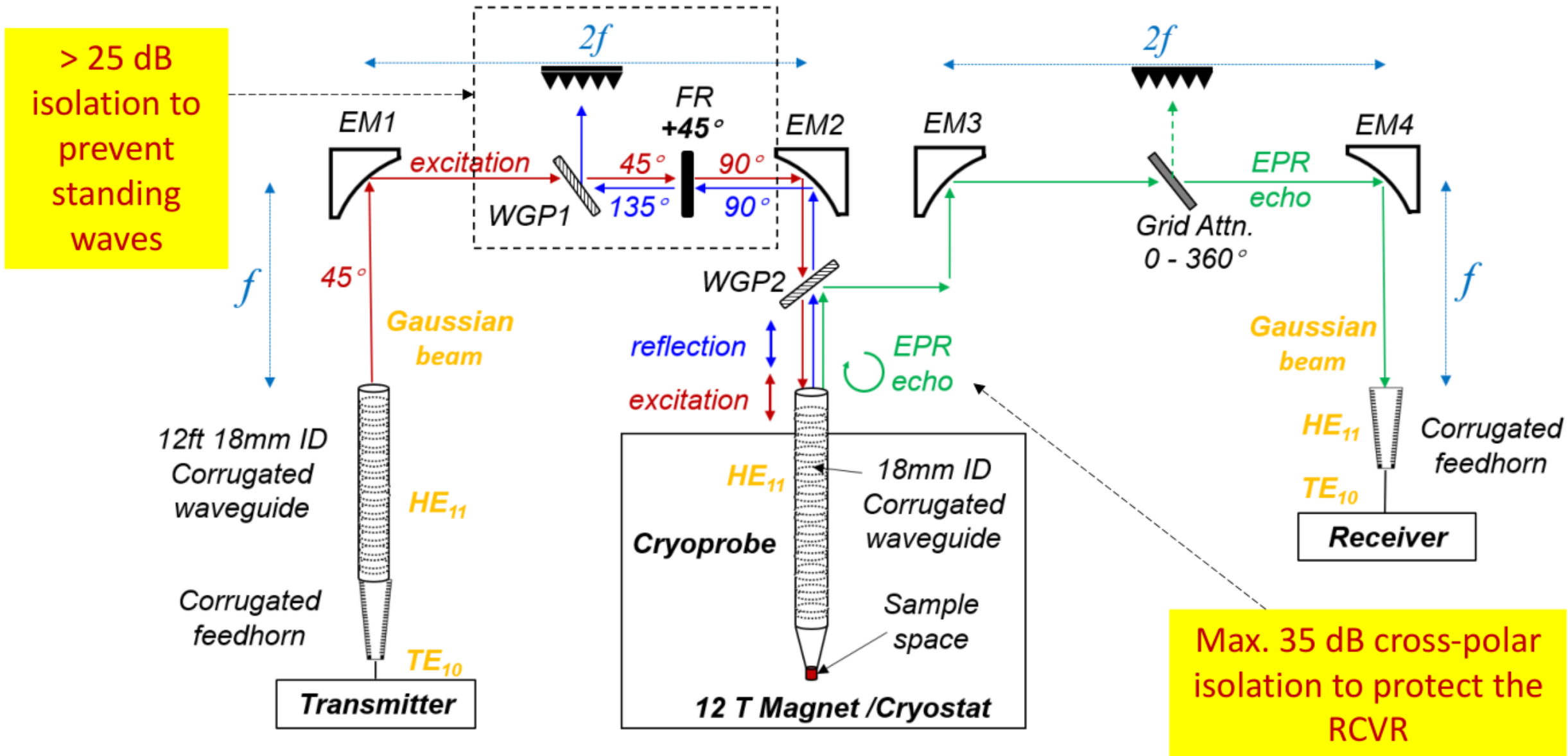

Supplementary Figure 4: Schematic diagram of quasi-optical (QO) free-space TEM00 Gaussian beam and corrugated waveguide HE11 mode components and the pulse flow in the QO circuit.

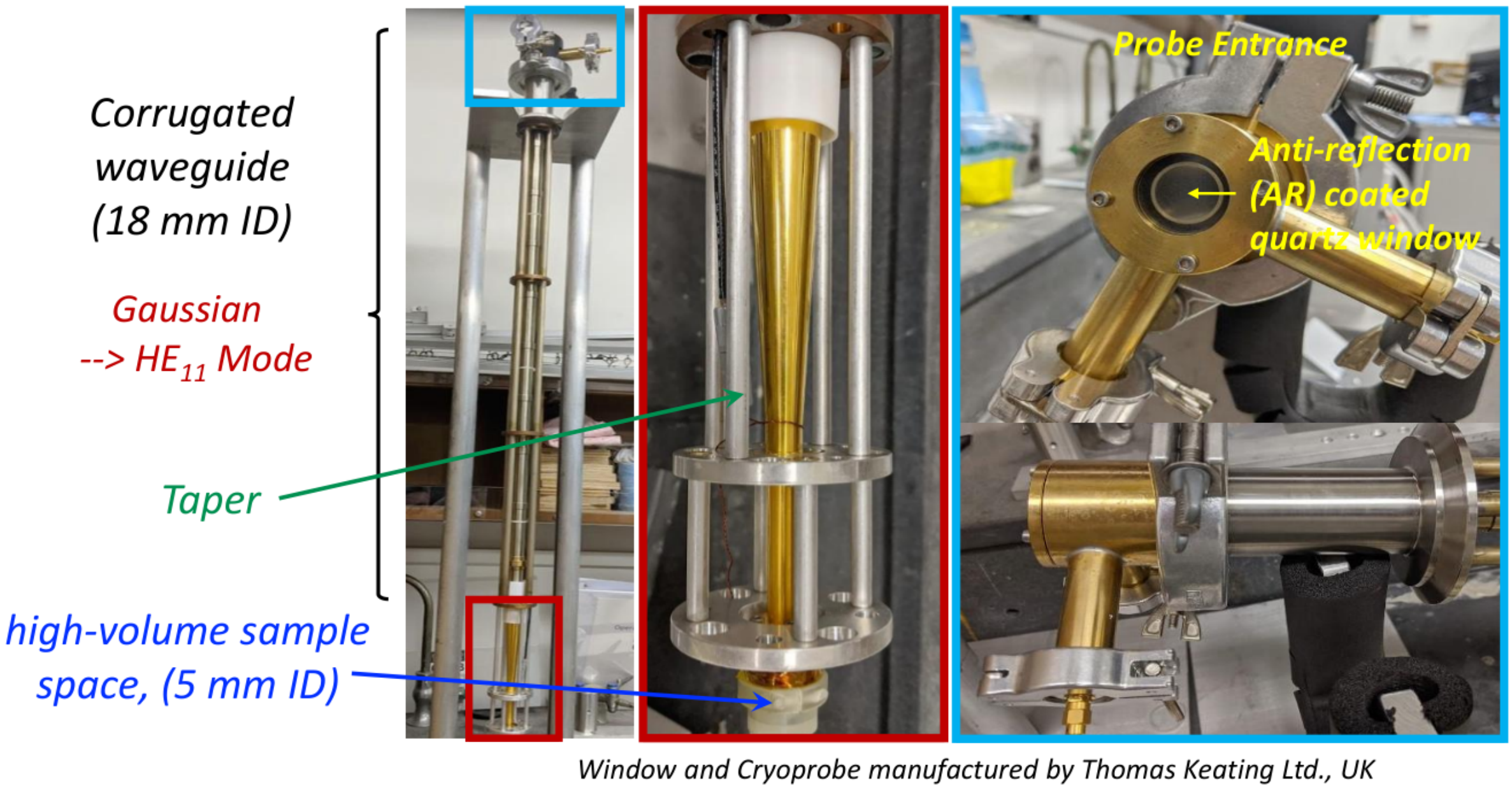

Supplementary Figure 5: Detail of the 1.16 m long probehead showing radiation baffles, anti-reflection coated quartz window, 18 mm to 5 mm ID taper and 50 mm long 5 mm ID sample section.

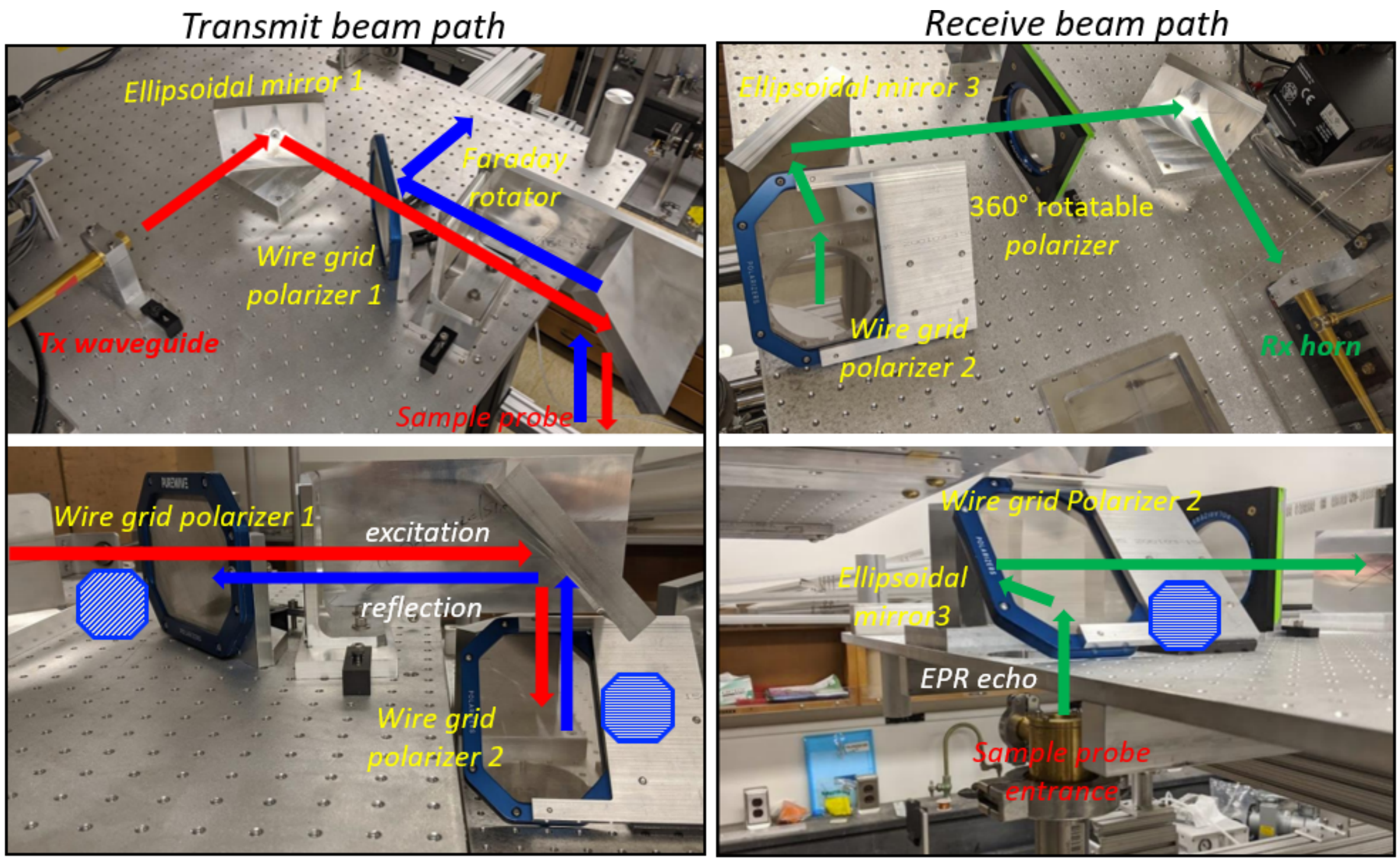

Supplementary Figure 6: Beam path on both the transmitter and receiver plate. A quasi-optic flat ferrite Faraday rotator-based isolator with better than 25 dB isolation and 2 dB of insertion loss is employed on the transmitter plate to reduce reflections back towards the TWT. So far no similar isolator has been needed on the receiver plate.

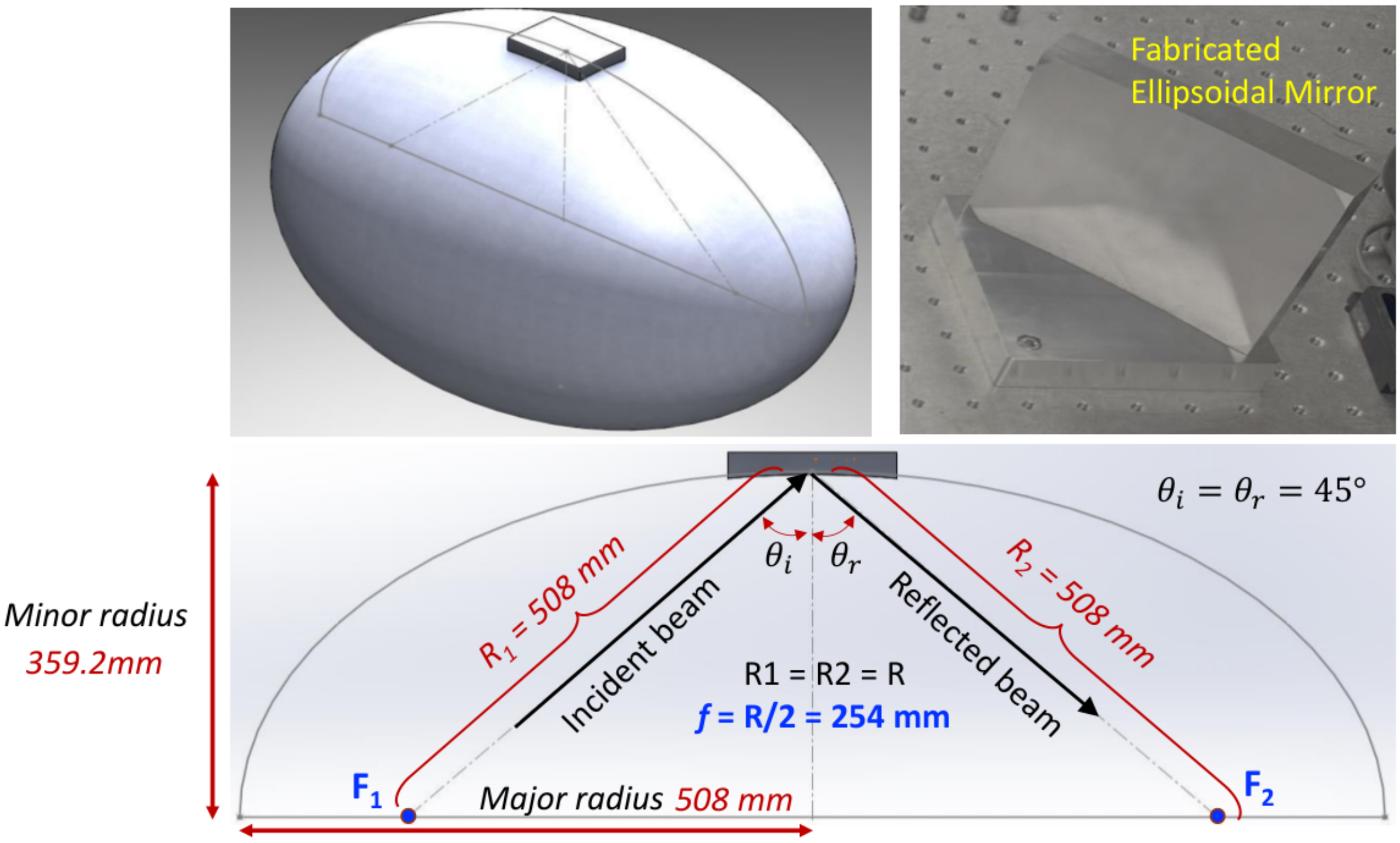

Supplementary Figure 7: Ellipsoidal 90°turning mirror design showing the mirror's concave surface's relationship to the parent ellipsoid and the chosen design focal length, f, of 254 mm (10 in).

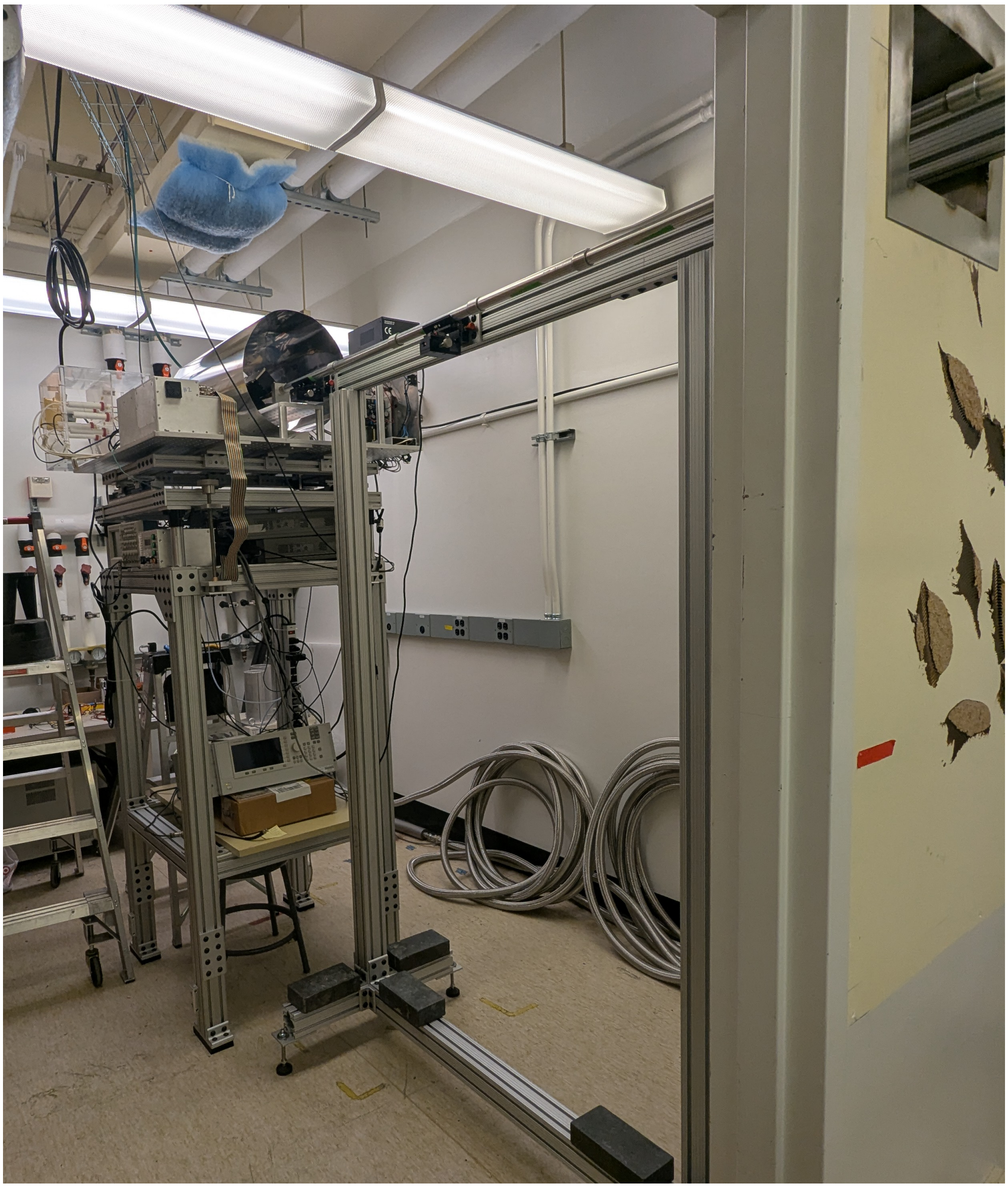

Supplementary Figure 8: The spectrometer's transmitter assembly including the TWT tube and its high voltage modulator and modulator control box, mu-metal magnetic shield and helically corrugated waveguide, 12 ft.

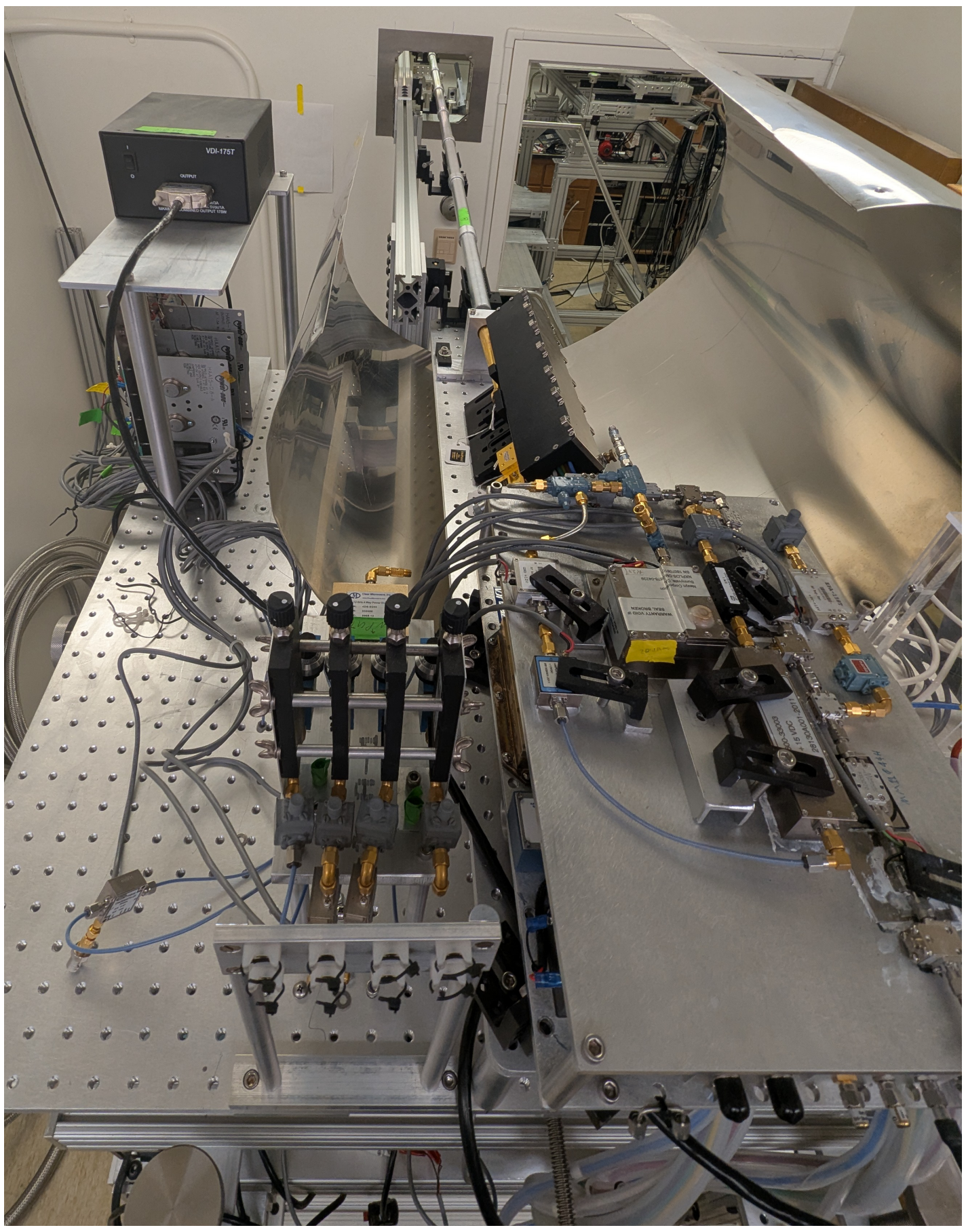

Supplementary Figure 9: The spectrometer's transmitter coaxial assembly including a VDI-AMC (lower plate) and supporting microwave components (upper plate) as the driver for the TWT input.

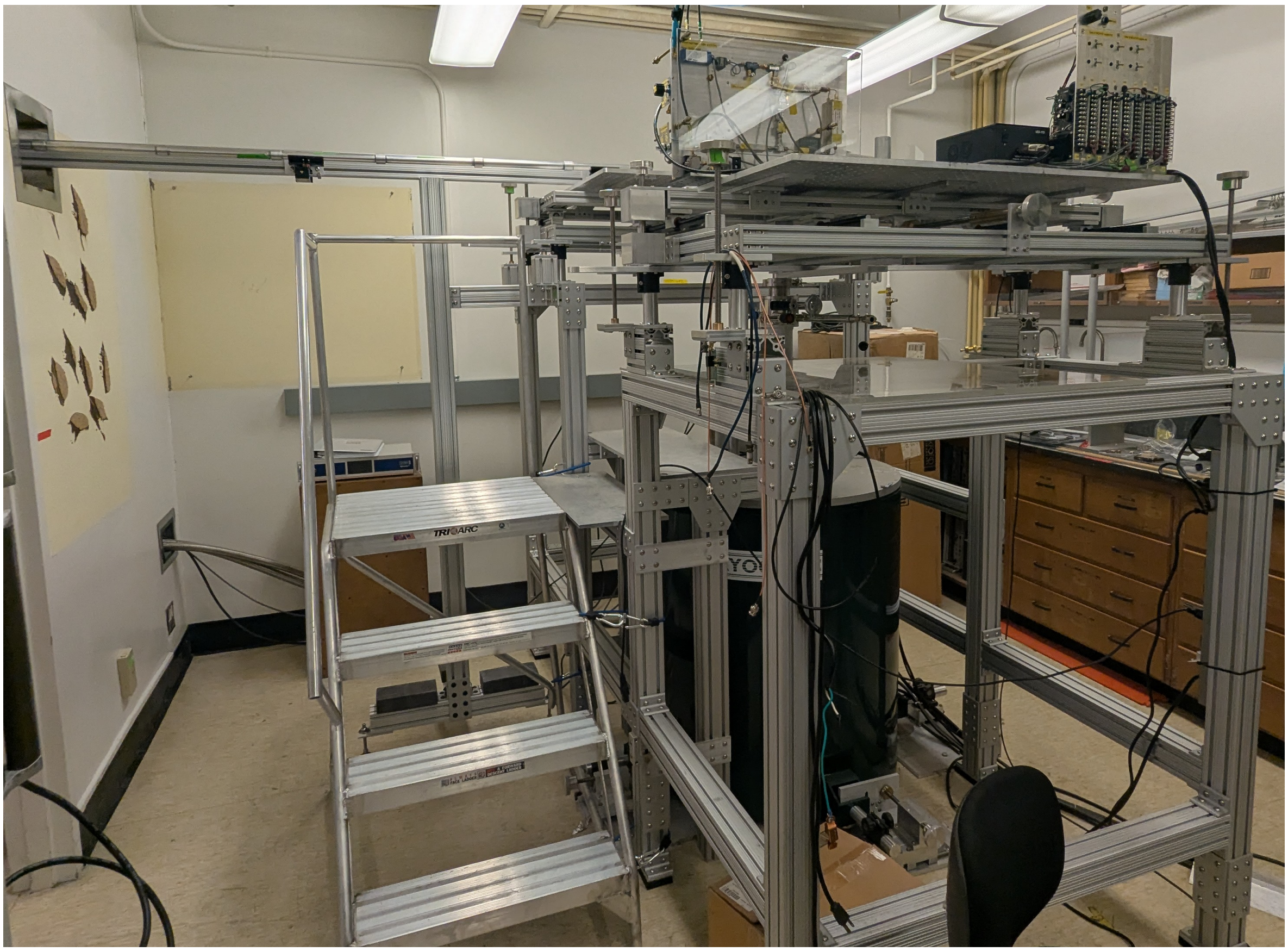

Supplementary Figure 10: The spectrometer's transmitter plate and receiver plate (foreground).

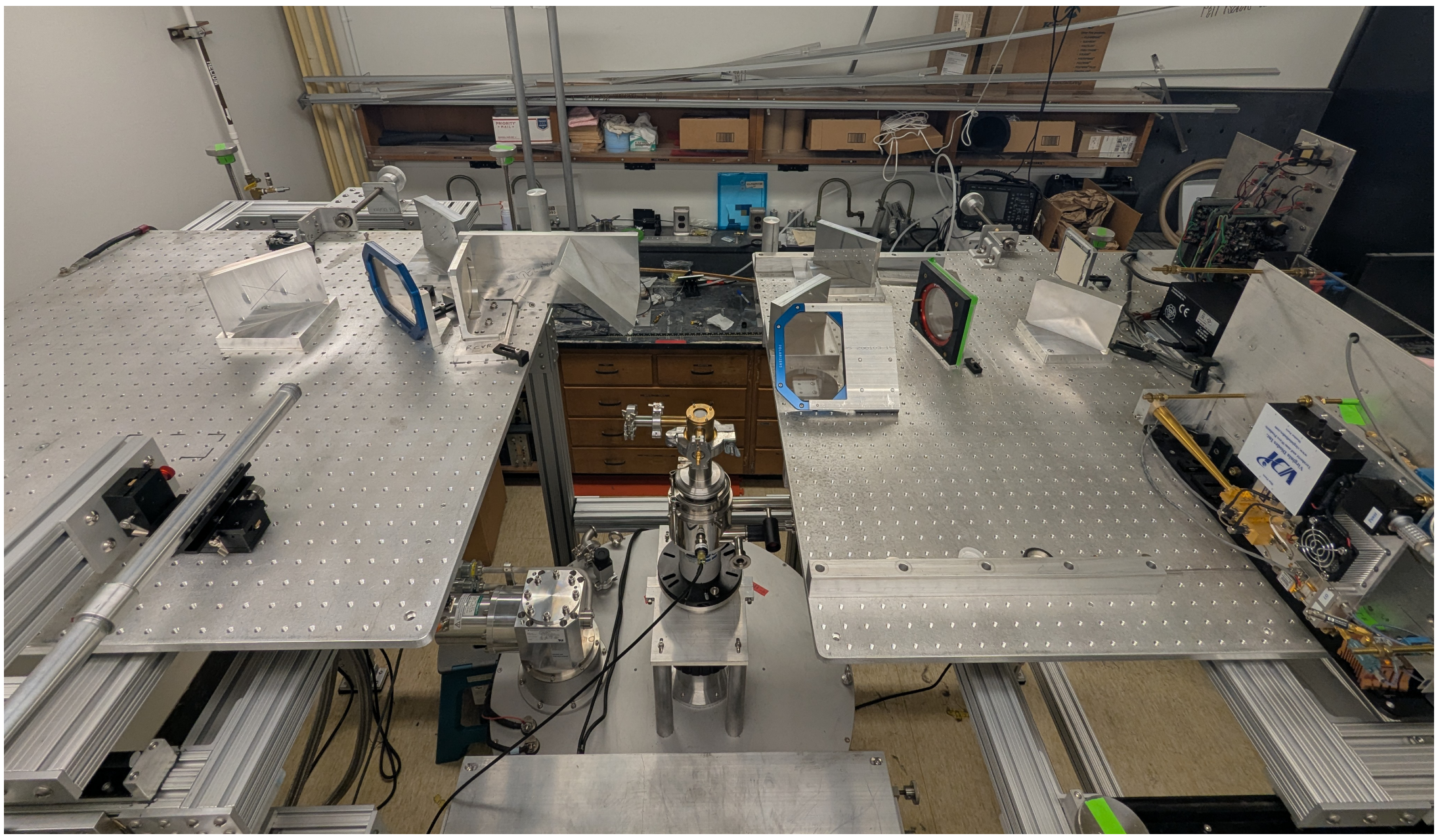

Supplementary Figure 11: The spectrometer's transmitter plate, TXMR (left), and receiver plate, RCVR (right), spread apart to permit insertion and removal of the probehead.

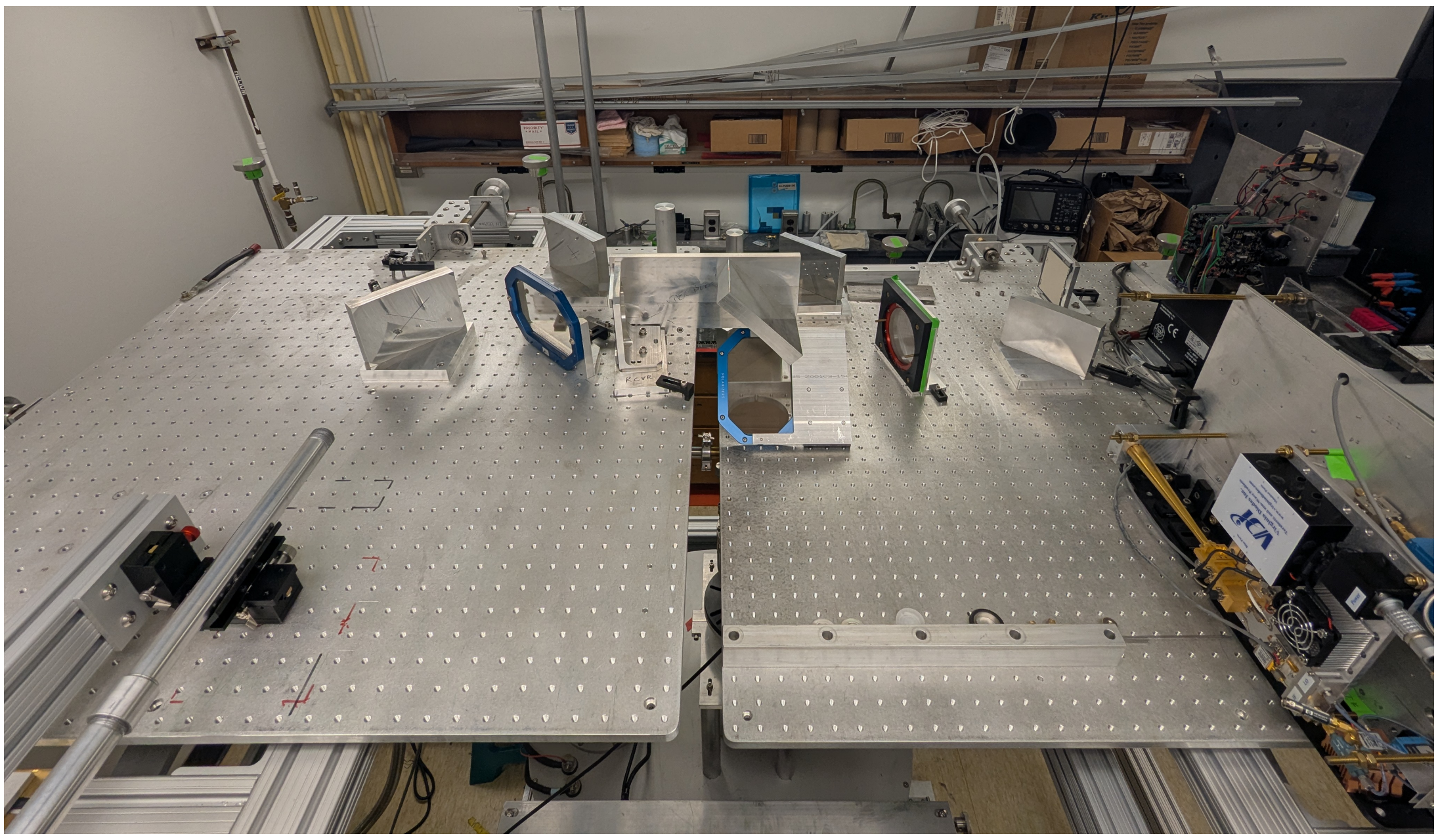

Supplementary Figure 12: The spectrometer's transmitter plate, TXMR (left,) and receiver plate, RCVR (right,) closed and aligned over the probehead and ready for use.

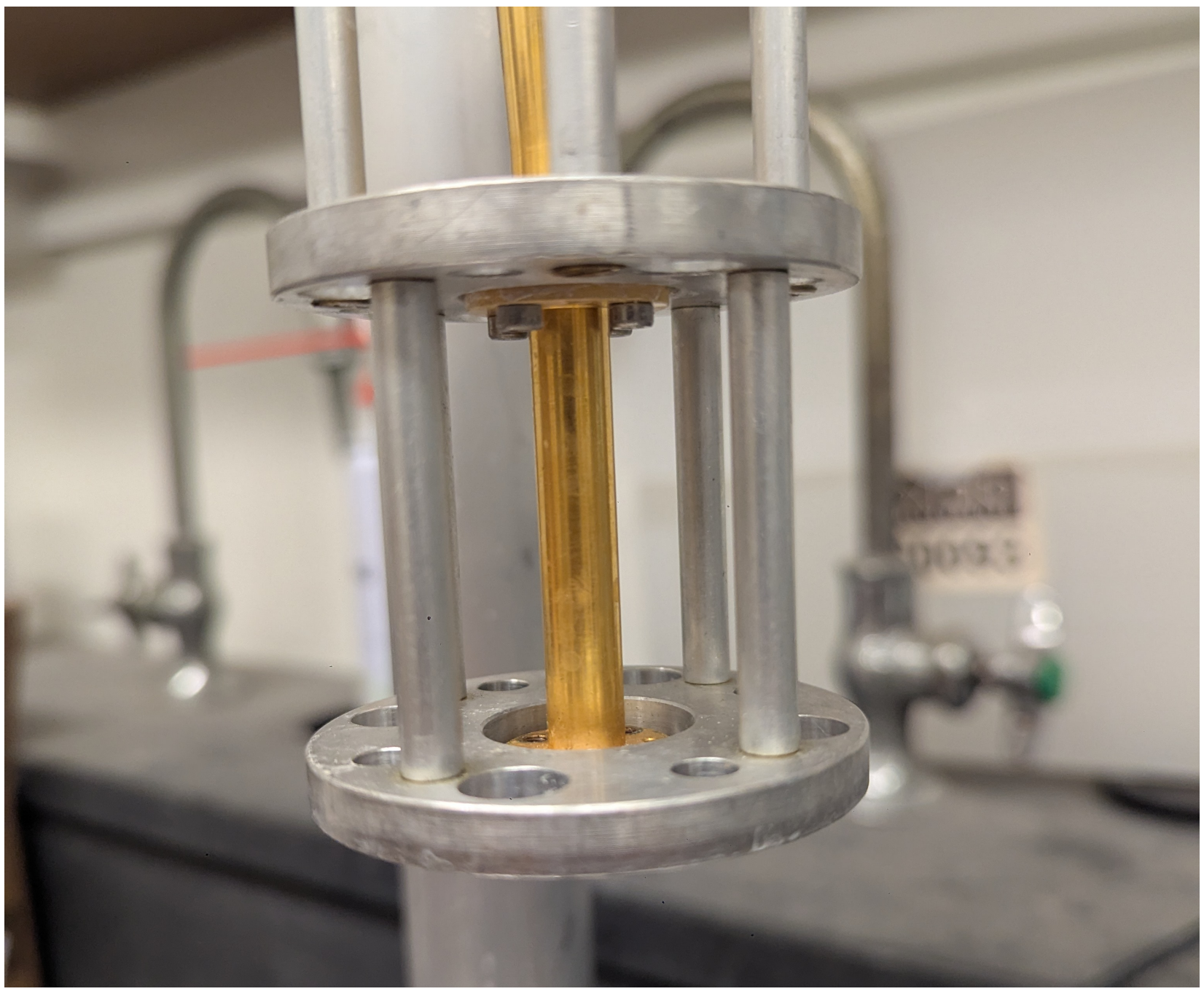

Supplementary Figure 13: Shown is the probehead's sample space waveguide section which is a 50 mm long section of corrugated waveguide with a 5 mm ID.

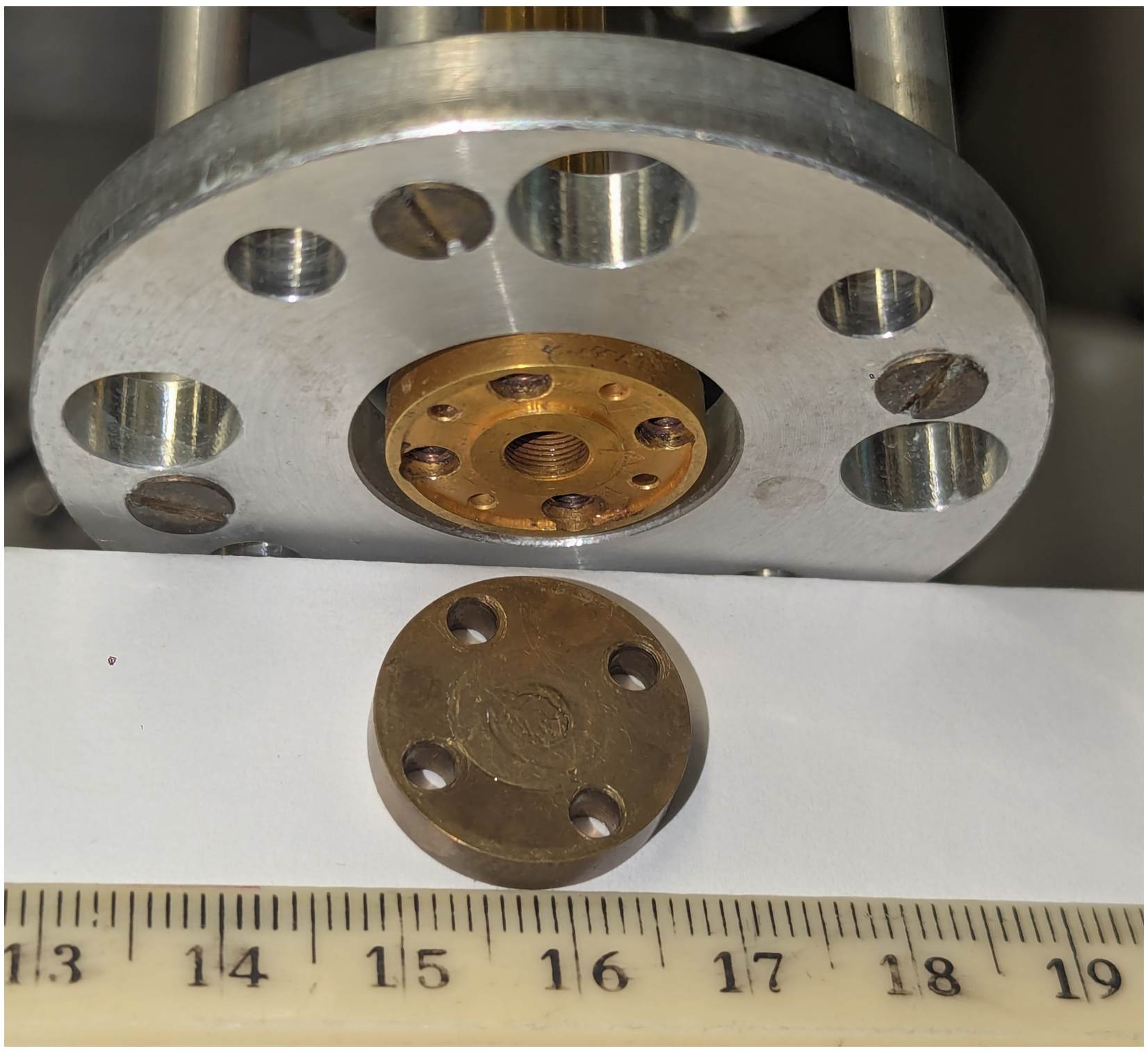

Supplementary Figure 14: Shown is the probehead's non-resonant sample space where a sample is placed on the center of the flat reflector plate. The plate is then attached to the 5 mm ID waveguide.

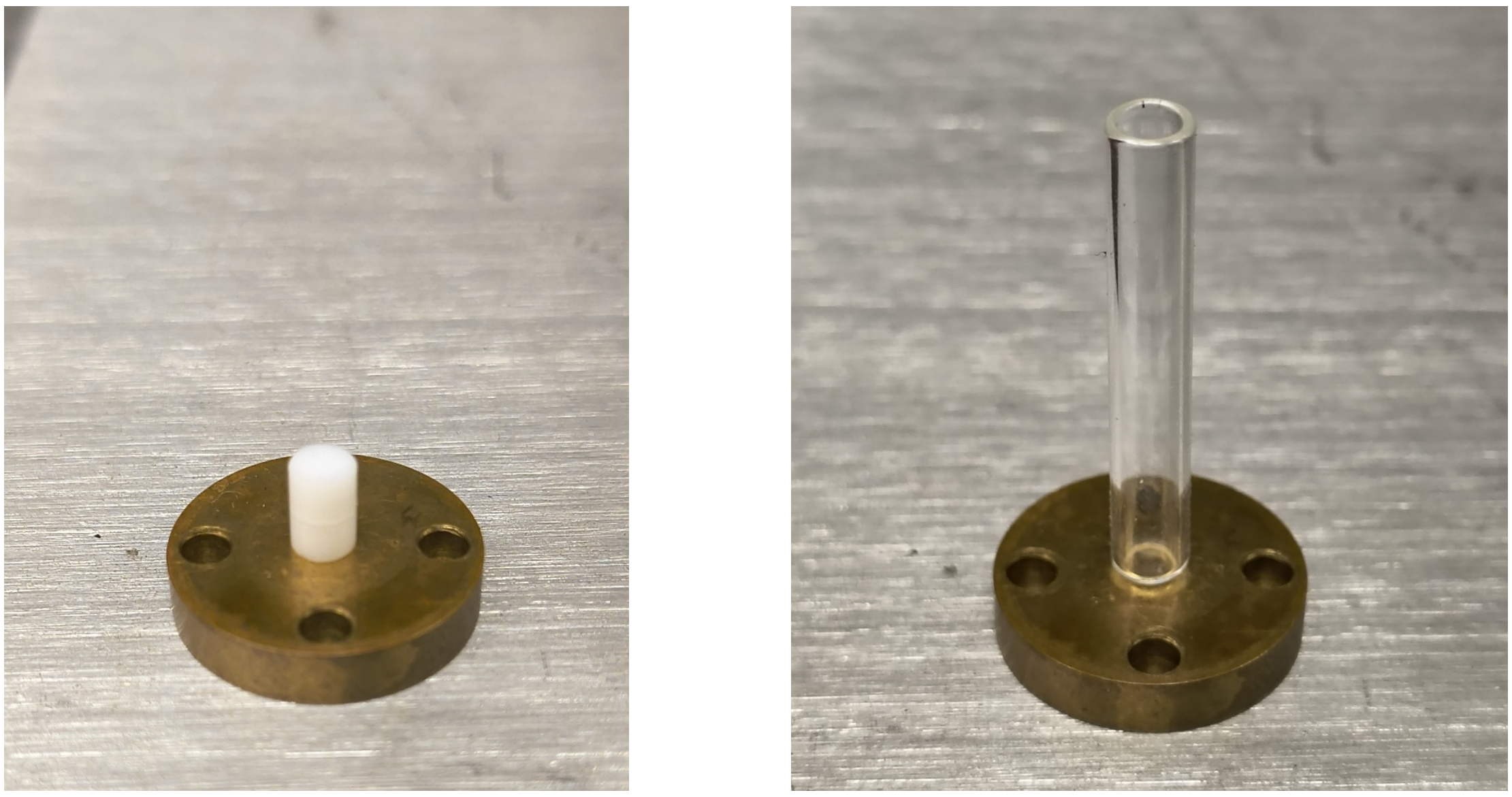

Supplementary Figure 15: Left: 4-mm OD teflon 'cup'. Right: Flat-bottom 4.51-mm OD tube. Both are examples of possible sample holders.

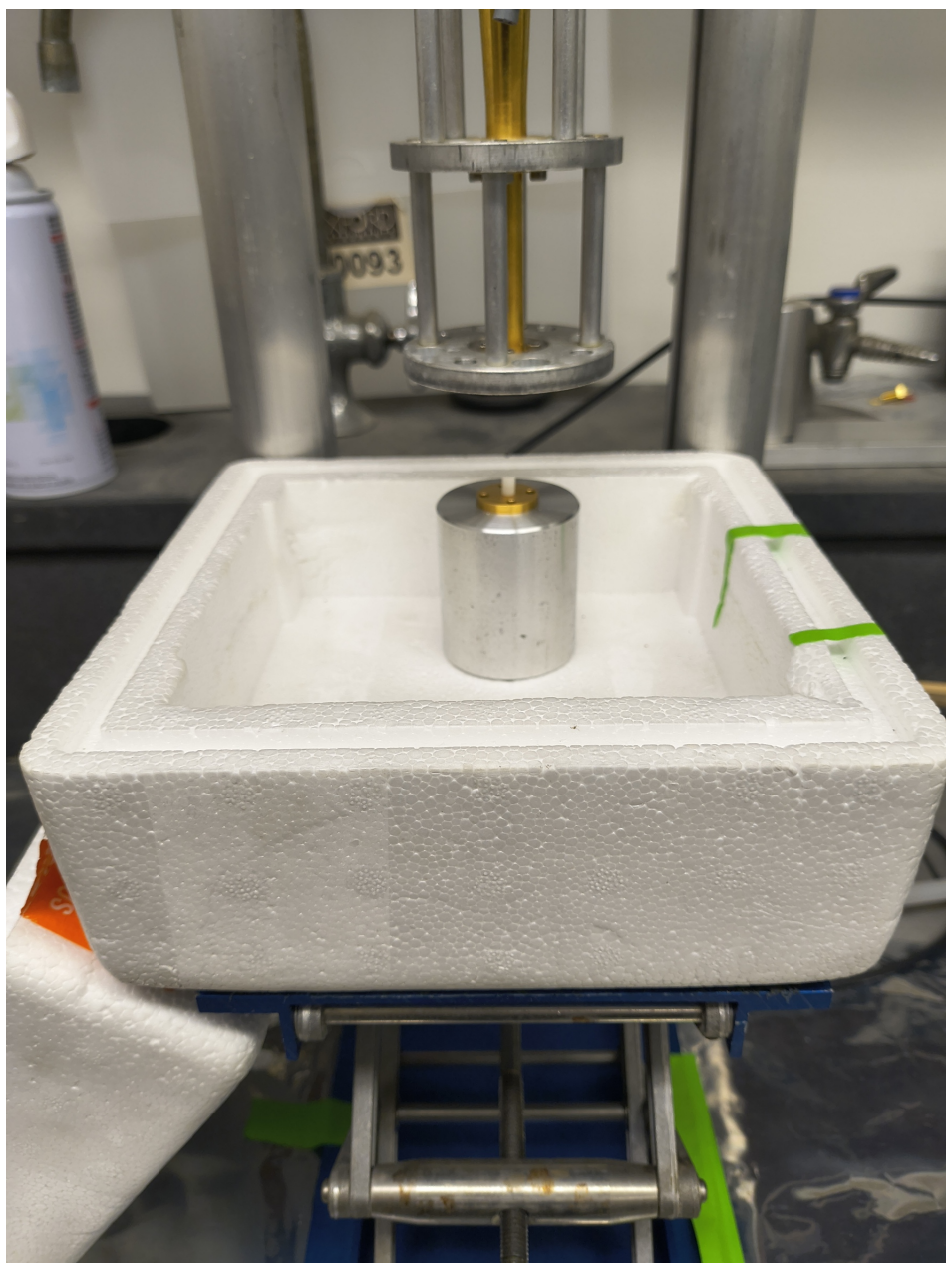

Supplementary Figure 16: Shown is the loading setup of the teflon cup into the probehead. The reflector plate lies on a cylindrical aluminum block inside a box that can be filled with liquid N_2 to maintain the cryogenic temperature of the sample while loading. The whole box can be elevated to load the sample and attach the reflector plate with the probehead.

1.2 Pulse EPR Operation

For the MgO powder experiments at 263 GHz, 20 mg of MgO powder was used to fill the Teflon sample holder. All solution samples were prepared with 25% ethylene glycol by volume in Teflon sample holders. Dow Corning High Vacuum grease was used to keep the sample in place as the reflector plate was attached to the probehead. The sample and probehead were inserted into the Spectrostat CF Cryostat (Oxford Instruments), which can achieve temperatures as low as 1.37 K. Liquid Helium flow was controlled using a GF4 pump and a VC-U gas flow controller, and the temperature was monitored using a MercuryITC temp controller. The spectrometer was controlled using Specman4EPR[3].

Two pulse echoes of MgO were collected at X-band, Q-band, and 263 GHz. The X-band (9.440 GHz) spectrum of MgO was collected using $\pi/2$-τ-π of 12 ns-300 ns-24 ns and averaged from 512 scans with a 1 kHz repetition rate. The pulse lengths were shorter than those at 263 GHz to achieve the best X-band spectrum. The data was collected using Bruker ELEXSYS-E580 spectrometer equipped with a 1 kW TWT amplifier, an ER 4118X-MS5 resonator, and an Oxford Instruments CF935 continuous-flow liquid helium cryostat. On the other hand, the Q-band spectrum (34.002 GHz) was collected using $\pi/2$-τ-π of 30 ns-300 ns-60 ns and averaged from 512 scans with a 1 kHz repetition rate. The pulse lengths were chosen to best match the parameters used for 263 GHz without minimal signal compromise. The Q-band spectrum was collected using a Bruker ELEXSYS-E580 spectrometer equipped with a 100 W solid-state amplifier, a Bridge12 QLP-QT2 resonator, and a Bruker ColdEdge WaveGuide Cryostat.

Two-pulse echoes at 263 GHz were collected using a sequence of $\pi/2$-τ-π with lengths of 30 ns-300 ns-60 ns. These parameters were based on Rabi nutation experiments of Mn(II) discussed in Supplementary Material Section 3.5. The echoes were averaged from 512 scans with a 1 kHz repetition rate, except for Mn-EDTA and Mn-PsaA, where the echoes were averaged from 1024 scans to ensure a sufficient signal-to-noise ratio with their overall broader spectra. The signal from the spectrometer receiver was digitized using an Acqiris U5303A PCIe ADC card with on-board processing and 1.6 GS/s sampling rate. Echoes were collected as the magnetic field was swept at a rate of 1.6 G/s. The areas of the echoes were then integrated to obtain the amplitude of the two-pulse echo spectra of the Mn samples. Each spectrum of Mn solution samples was simulated using EasySpin 6.0.10[11], with the esfit program. The genetic algorithm was used to avoid the fitting of local minima. The parameters from esfit served as initial parameters for further fine tuning in the next simulation as each transition must be weighted appropriately. The simulations were done with the 'transitions' set in the 'separate' option, which allows for the spectrum to be separated by their individual transitions. Each transition was then weighted based on the effective flip angle of each transition [12]. Details of how each transition affects the Rabi nutation and flip angle are discussed in Supplementary Material Section 3.5.

Three-pulse Rabi nutation experiments were collected using the sequence $p_1 - t_1 - \pi/2 - t_2 - \pi$, where p_1 represents the first pulse and t_n represents the n-th time separation. In each case, $\pi/2$ and π were set to best match the observed π rotation. Parameters t_1 and t_2 were set as 200 ns and 500 ns, respectively. The Rabi nutation experiment measures the integrated echo area as a function of p_1 length, which were set as 0 ns and increased by 4-ns steps.

2 Supplementary Table

Supplementary Table 1: Concentration of transition metals in the nominal MgO sample determined by ICP-MS

Element	Concentration (ng/mg MgO)	Detection limit (ng/mg)
V	2.231	0.010
Cr	16.387	0.002
Mn	25.728	0.002
Fe	314.145	0.020
Ni	18.198	0.010
Co	0.192	0.002
Cu	1.856	0.010
Zn	4.966	0.010

Supplementary Table 2: List of 263 GHz pulse spectrometer transmitter (TXMR) components

Component List			
Number	Manufacturer	Model	Details
1	Nexyn Corp.	NXPLOS-DM-0876-04239	PLDRO, 8.7667 GHz, 20 dBm Output
2[a]	Herley-CTI	XS-7314	Synth., 8.7667 $\pm$ 0.377 GHz, 10 Hz res., 15 dBm Output
3	Narda Microwave	4015C-20	7-12.4 GHz, 20 dB Coupling
4, 31	Narda Microwave	4015C-10	7-12.4 GHz, 10 dB Coupling
5, 6	Clear Microwave	4DS-9D00	6-12 GHz, 4-way Power divider / combiner
7-10	Arra Microwave	T6804-20	8-12.4 GHz, Variable level-set, 20 dB
11-14	Sage Laboratories	6705K-14	DC-26.5 GHz, Var. mech. phase shifter
15-18, 27, 40	Teledyne Microwave	T-5S73T-3	5.9-13 GHz, 17 dB Isolation
19-22, 25[a]	Custom Microwave Components	CMCS0947A-C2	0.1-18 GHz, 3.5 dB IL, 60 dB Isolation, 1 ns rise/fall time
23[a]	Minicircuits.	ZVA-183-S+	0.7-18 GHz, 26 dB Gain, 24 dBm P1dBm Output
24[a]	Narda Microwave	4915	7-10 GHz, 20 dB Isolation
26	Advanced Technical Materials	P216-4315-2	8-12.4 GHz, 2-way Power divider / combiner, 20 dB isolation
28	RF Bay, Inc.	EPA-158T	6-12 GHz, 19 dB Gain, 19 dBm P1dBm Output
29	Narda Microwave	4946	8-18 GHz, 16 dB Isolation
30	Marki Microwave	MM1-1044H	10-44 GHz RF/LO, DC-14 GHz IF, 9 dB CL
32	Marki Microwave	AQA-2156	21-56 GHz, x4 Active frequency multiplier, 20 dBm Output
33, 46	Marki Microwave	FB3270	BPF, 32.4 GHz CF, 9.88 GHz BW, 2.4 dB IL
34	Ditom, Inc.	D3I2004	20-40 GHz, 10 dB Isolation, 2 dB IL
35	RF Bay, Inc.	FPS-4-13	0.1-13 GHz, $\div$4 Active frequency divider, 5 dBm Output

Continued on next page...

Number	Manufacturer	Model	Details
36	Minicircuits	BW-S7W2+	DC-18 GHz, 7 dB, 2 W, Attenuator, SMA
37	Minicircuits	ZX60-6013E-S+	0.02-6 GHz, 16 dB Gain, 13 dBm P1dBm Output
38	Wenzel Associates	600-33003	6.321-6.827 GHz, x3 Active multiplier, 13 dBm Output
39	K&L Microwave	4FV20-6575/H510-O/O	BPF, 6.575 GHz CF, 0.51 GHz 0.5 dB-BW, 0.5 dB IL
41	Marki Microwave	ML1-1050I	10-50 GHz RF/LO, DC-16 GHz IF, 9 dB CL
42	Marki Microwave	FB4000	BPF, 40.18 GHz CF, 13.32 GHz BW, 2.4 dB IL
43, 47	Marki Microwave	A2050	20-50 GHz, 23 dB Gain, 15 dBm P1dBm Output
44	Ditom, Inc.	D3I4043	40-43.5 GHz, 18 dB Isolation, 1.2 dB IL
45	Spincore	PBESR-Pro-400	Pulseblaster PC card, digital word/pattern generator, 21 ch, 2.5 ns resolution, 400 MHz
48	Ditom, Inc.	D3I2640	26.5-40 GHz, 14 dB Isolation, 1.0 dB IL
49	Minicircuits	BW-K10-2W44+	DC-40 GHz, 10 dB, 2 W, Attenuator, 2.92 mm
50	Minicircuits	BW-K2-2W44+	DC-40 GHz, 2 dB, 2 W, Attenuator, 2.92 mm
51	Virginia Diodes	VDI-AMC-721	Amplifier-Multiplier Chain, x8, 260-265 GHz, 23.2 dBm (215 mW) Output at 263 GHz
52	Eravant mmWave	680J-LH/387, STA-03-03-F1, STA-30-03-M1-C-1.2, SWD-2020H-03-SB, SWB-03090-EB	WR-3.4 waveguide: 45 deg. LH Twist (MI-Wave, FL), 3 dB fixed precision attenuator, micrometer 30 dB variable attenuator, 20 dB dir. coupler, 90 deg E-plane bend
53	Eravant mmWave	SWB-03090-EB	WR-3.4 waveguide, E-plane bend
54	Thomas-Keating UK	Project 23472	263 GHz Corrugated horn antenna, 18 mm ID circular aperture, WR-3.4 waveguide aperture
55	Beijing Vacuum Electronics Research Institute (BVERI)	TWT Amplifier	WR-3.4 input/output, Custom folded waveguide 10 W pulsed traveling wave tube, 258–264 GHz 10 W-BW, 1 $\mu s \leq$ pulse length $\leq$ 20 μs, Maximum duty cycle 10%, Maximum repetition rate 5 kHz
**End of Table**			

[a] Second channel components for EDNMR experiments to be incorporated and may include an attenuator and a phase shifter.

Supplementary Table 3: List of 263 GHz pulse spectrometer receiver (RCVR) components

Component List			
Number	Manufacturer	Model	Details
56	Micro-Coax, Inc.	UtiFlex UFA210B	DC-26.5 GHz, Flexible coaxial cable, 20 ft, approx. 5.5 dB IL at 8.7667 GHz
57	CTT, Inc.	APO/120-2520-30	6-12 GHz, 20 dB Gain, 20 dBm P1dBm Output
58	Teledyne Microwave	T-7S43T-15	7-11 GHz, 28 dB Isolation, 0.4 dB IL
59	MAC Technology	C3206-10	7-12.4 GHz, 10 dB Coupling
60	Marki Microwave	AQA2040	20-40 GHz, x4 Active frequency multiplier, 20 dBm Output
61	Marki Microwave	FB3270	BPF, 32.4 GHz CF, 9.88 GHz BW, 2.4 dB IL
62	Ditom, Inc.	D3I2004	20-40 GHz, 10 dB Isolation, 2 dB IL
63	Omni-Spectra	2020-6622-20	7-12.4 GHz, 20 dB Coupling
64	Nexyn Corp.	NXD-0880/8-04239	8-9.6 GHz, ÷8 Active frequency divider, 13 dBm Output
65	Minicircuits	BW-S2W2+	DC-18 GHz, 2 dB, 2 W, Attenuator, SMA
66	Wenzel Associates	600-29611	3.12-3.45 GHz, x3 Active frequency multiplier, 12 dBm Output
67	Minicircuits	BW-S4W2+	DC-18 GHz, 4 dB, 2 W, Attenuator, SMA
68	Teledyne Microwave	T-2S73T-6	2-4.5 GHz, 16 dB Isolation, 0.6 dB IL
69	Marki Microwave	ML1-1144I	11-44 GHz RF/LO, DC-21 GHz IF, 7 dB CL
70	ATMI-Microwave Circuits	B1031G81	BPF, BPF, 31.8 GHz CF, 4.22 GHz 3 dB-BW, 1.3 dB IL
71	CTT, Inc.	ALW/400-6010	26.5-40 GHz, 10 dB Gain, 12 dBm P1dBm Output
72	Ditom, Inc.	D3I2640	26.5-40 GHz, 14 dB Isolation, 1.0 dB IL
73	Inmet, Inc.	40AH-3	DC-40 GHz, 3 dB, 2 W, Attenuator, 2.92 mm
74	Virginia Diodes	VDI-MixAMC-252	Subharmonic Mixer, Amplifier-Multiplier Chain, x8, 240-272 GHz RF, 30-34 GHz LO, 25 kHz-20 GHz IF, 6 dB CL at 263 GHz
75	Thomas-Keating UK	Project 23472	263 GHz Corrugated horn antenna, 18 mm ID circular aperture, WR-3.4 waveguide aperture
76	Eravant mmWave	SWB-03090-EB	WR-3.4 waveguide, E-plane bend
77	Minicircuits	ZX60-06183LN+	6-18 GHz, 20 dB Gain, 15 dBm P1dBm Output, 2.8 dB NF
78	Arra Microwave	T6804-20	8-12.4 GHz, Variable level-set, 20 dB
79	Minicircuits	ZX60-183-S+	6-18 GHz, 24 dB Gain, 18 dBm P1dBm Output
80	Marki Microwave	FB0860	BPF, 8.6 GHz CF, 1.3 GHz 3 dB-BW, 1.9 dB IL
81	Marki Microwave	MLIQ-0416L	4-16 GHz RF/LO, DC-3.5 GHz IF, 8.5 dB CL, IQ mixer

Continued on next page...

Number	Manufacturer	Model	Details
82, 83	Avtech Electrosystems Ltd.	AV-141C1	DC-800 MHz, x10 A_V Vol. Gain , 0.8 ns rise/fall time, ± 3 V Max. output
84, 86	TRW Microwave	ASI-7011	7-11 GHz, 20 dB Isolation, 0.5 dB IL
85	Arra Microwave	9426A	DC-18 GHz, Mech. phase shifter, SMA
**End of Table**			

3 Supplementary Discussion

3.1 10 W 263 GHz pulse EPR performance

We determine the optimal pulse lengths by performing Rabi nutation experiments with the 10 W TWT. Specifically, we use the OX071 radical (O2M Technologies), which is a hydrophylic trityl derivative, as a starting sample. This radical is particularly useful due to its exceptionally sharp spectrum, as depicted in Supplementary Figure 17. From the OX071 spectrum, we estimate that we can detect 1.0*10^{14} spins with an S=1/2 for an S/N of 10, which we arbitrarily set as our limit of detection. Note that we are using OX071 as a sharp and simple S=1/2 radical, which differs from the high-spin systems discussed in the main article. The sharpness of the spectrum leads to no complications in the Rabi nutation from orientational effects, and the narrow range of resonant frequencies minimizes damping of the signal in the Rabi nutation experiment.

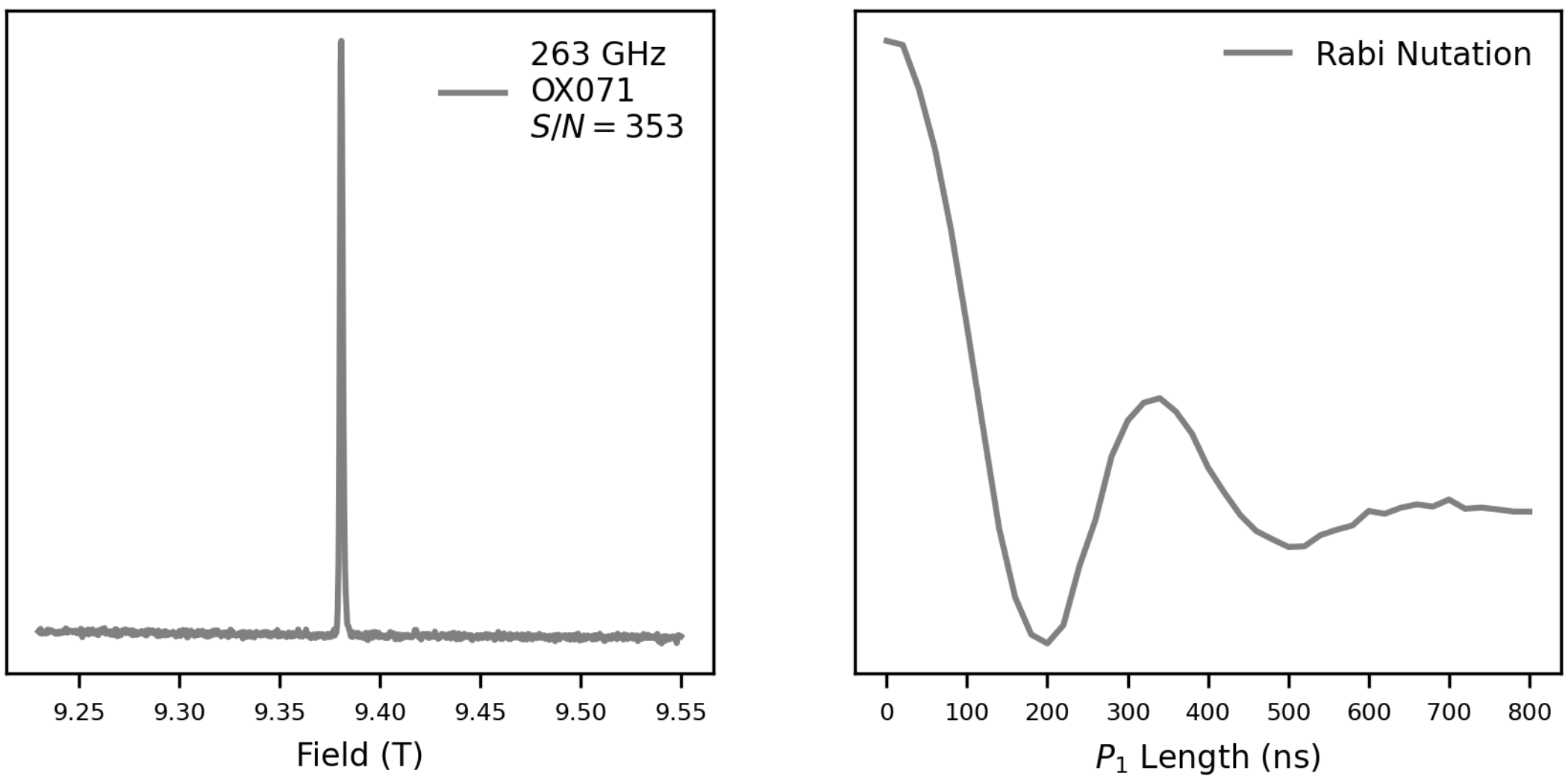

Supplementary Figure 17: Left: Echo-detected spectrum of 12 μL, 500 μM OX071 trityl in water and 25% ethylene glycol at 80 K, 263 GHz. Right: Rabi nutation of OX071. The optimal π-pulse for OX071 is 200 ns.

Overall, we observe that a pulse length of 200 ns results in the minimum point of the Rabi nutation, which corresponds to a π rotation of the OX071 spins. For context, the π rotation for BDPA (S=1/2) is 750 ns in the Bruker E780 at 263 GHz without a resonator [13]. Hence, the 200 ns π-pulse is a significant improvement enabled by the 10 W TWT.

3.2 Mn(II) lineshape analysis

The effective spin Hamiltonian for Mn(II) with $S > \frac{1}{2}$ and isotropic g value and hyperfine is as follows:

$$\hat{H} = \hat{H}_Z + \hat{H}_{HF} + \hat{H}_{ZFS} \quad (1)$$

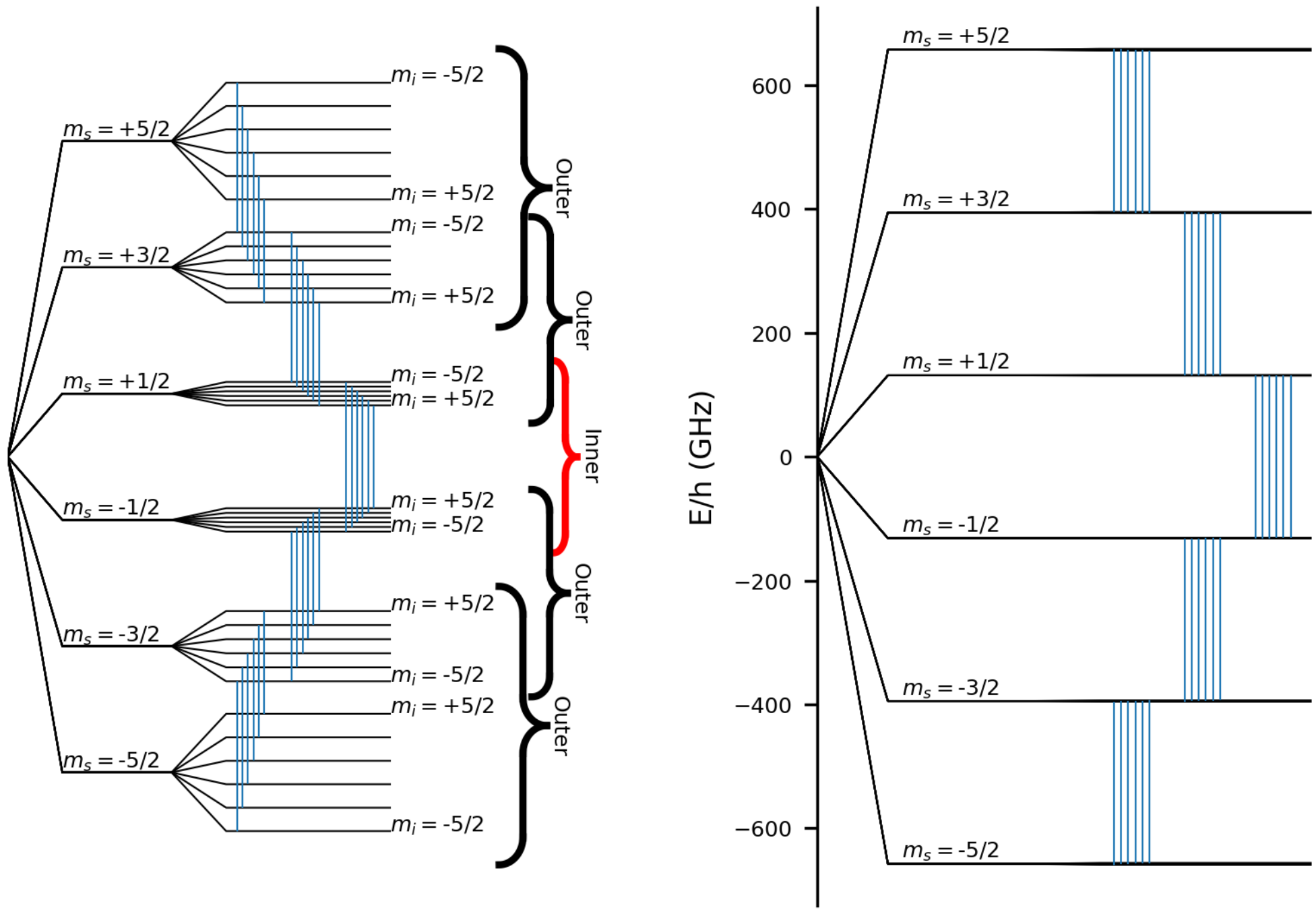

Supplementary Figure 18: Left Panel: Energy diagram of Mn with $S = 5/2$ and $I = 5/2$. The allowable EPR transitions $\Delta m_s = \pm 1$ are depicted as solid blue lines. The $m_s = -\frac{1}{2}$ to $m_s = \frac{1}{2}$ transition is labeled as Inner transition, while the rest are labeled as Outer transitions. Right Panel: Energy diagram of Mn with accurate scaling of the energy differences between states at 263 GHz and 4.2 K. The depiction assumes $g = 2$.

$$\hat{H} = g\beta_e \mathbf{B_0}\hat{S} + A\hat{S}\cdot\hat{I} + \hat{S}\cdot\tilde{D}\cdot\hat{S} \tag{2}$$

where $\mathbf{B_0}$ is the applied magnetic field, A is the hyperfine constant, $\tilde{D}$ is the ZFS tensor, $\hat{S}$ is the electron spin operator, and $\hat{I}$ is the nuclear spin operator. With the $\tilde{D}$ being traceless, the ZFS term can be further described with scalar parameters D and E:

$$\hat{H}_{ZFS} = \frac{D}{3}[3\hat{S}_z^2 - S(S-1)] + E[\hat{S}_x^2 - \hat{S}_y^2] \tag{3}$$

$$D = \frac{3D_z}{2}, E = \frac{D_x - D_y}{2} \tag{4}$$

Note that the ZFS is a purely anisotropic component of the spin Hamiltonian. Hence, we can further describe the $\hat{H}_{ZFS}$ as [14]:

$$\hat{H}_{ZFS} = [\frac{D}{3}(3cos^2\theta - 1) + \frac{E}{2}sin^2\theta cos2\phi][3\hat{S}_z^2 - S(S+1)] \tag{5}$$

where θ and ϕ are the zenith and azimuth angles in terms of the orientations of the molecular axes with respect to the applied magnetic field.

In the limit of the high-field regime, the electron Zeeman interaction dominates, as depicted in Supplementary Figure 18. Consequently, the spin Hamiltonian can be rewritten for the perturbation treatment as:

$$\hat{H} = \hat{H}_0 + \hat{H}_1 \tag{6}$$

$$\hat{H}_0 = g\beta_e B_0\hat{S}_z \tag{7}$$

$$\hat{H}_1 = g_n\beta_n B_0\hat{I}_z + A\hat{S}\cdot\hat{I} + \frac{D}{3}[3\hat{S}_z^2 - S(S-1)] + E[\hat{S}_x^2 - \hat{S}_y^2] \tag{8}$$

where $\hat{H}_0$ is the zero order term and $\hat{H}_1$ is the first order perturbation.

Note that the ZFS term in the first-order perturbation scales with m_s^2. Hence, both $m_s = -1/2$ and $m_s = 1/2$ have the same ZFS contribution in the first order perturbation, leading to the Inner transition unaffected by ZFS and resonant fields described as [14]:

$$|\pm\frac{1}{2}, m_i\rangle \leftrightarrow |\mp\frac{1}{2}, m_i\rangle \; B = B_0 + Am_i \tag{9}$$

while the resonant fields of the outer transitions are broadened by the ZFS to the first order:

$$|\pm\frac{5}{2}, m_i\rangle \leftrightarrow |\pm\frac{3}{2}, m_i\rangle \; B = B_0 + Am_i \mp 2[D(3cos^2\theta - 1) + 3Esin^2\theta cos2\phi] \tag{10}$$

$$|\pm\frac{3}{2}, m_i\rangle \leftrightarrow |\pm\frac{1}{2}, m_i\rangle \; B = B_0 + Am_i \mp 2[D(3cos^2\theta - 1) + 3Esin^2\theta cos2\phi] \tag{11}$$

3.3 Intensity of Mn(II) EPR Transitions

At significantly low T and high B_0, the higher energy states become depopulated, affecting the intensity of Mn transitions in the spectra. Specifically, the intensity depends on the population difference of the $|m_s\rangle$ and $|m_s + 1\rangle$ states and their transition probability. The relative population of each state is as follows [15]:

$$P(m_s) = \frac{exp(-g\beta_e B_0 m_s/kT)}{\sum_{m_s=-S}^{S} exp(-g\beta_e B_0 m_s/kT)} \tag{12}$$

while the transition probability is as follows:

$$I(m_s, m_s + 1) = \frac{1}{4} g^2 \beta_e^2 B_1^2 [S(S + 1) - m_s(m_s + 1)] \tag{13}$$

From these equations, we can predict the signal intensity of each transition by multiplying the population difference of each transition by its probability, as shown in Supplementary Figure 19.

Here, we see that the lowest transition $|-5/2\rangle \rightarrow |-3/2\rangle$ continues to increase as T decreases. This effect is primarily due to the lowest energy state becoming more populated following the Boltzmann distribution. On the other hand, the upper transitions become more depopulated, leading to a decrease in the signal intensity at different T critical points. The behavior in the transition intensity is what leads to the asymmetrical Mn spectra at low T and high B_0.

Spectra of Mn can be easily understood if we consider Supplementary Figure 20. Let us assume a Mn system with axial symmetry (E=0) and ignore hyperfine for simplicity. The top panels correspond to a case where $D > 0$. The left panel shows the energy diagram as a function of B_0 when the $\mathbf{B_0}$ is parallel to the Z-direction of the molecular axis ($\theta = 0°$). At zero magnetic field, ZFS separates the energy levels between $m_s = \pm 1/2$, $m_s = \pm 3/2$, and $m_s = \pm 5/2$. Note that the order of the energy states follows the equation for $\hat{H}_{ZFS}$ and $\theta = 0°$. Then, with the application of $\mathbf{B_0}$, the Zeeman interaction further removes the degeneracy between the energy states. The double-headed arrows correspond to different positions where transitions $\Delta m_s = \pm 1$ for a given microwave excitation. The various resonances are depicted as spectral peaks of varying colors along B_0.

We can see the anisotropy effects of ZFS when we rotate our model such that $\mathbf{B_0}$ is parallel to the X-direction of the molecular axis, depicted in the middle panel. Note that at zero magnetic field, the order of the energy levels is reversed, where $m_s = \pm 5/2$ is lower than $m_s = \pm 1/2$, compared to when $\mathbf{B_0}||Z$. Consequently, the resonant fields vary with orientation.

The right panel highlights the spectral shape assuming a powder sample where the molecular orientations follow the expected statistical distribution. Only the $-5/2 \mapsto -3/2$ and $3/2 \mapsto 5/2$ transitions are depicted with zero linewidth for clarity. We can see that the spectrum of $3/2 \mapsto 5/2$ (purple) reflects the spectrum $-5/2 \mapsto -3/2$ (blue) along the center of the overall spectral breadth. However, the purple spectrum is less intense than the blue spectrum. This asymmetry is due to the fact that the higher energy states are depopulated at sufficiently high B_0 and low T, as highlighted by Supplementary Figure 19. More importantly, the asymmetry depends on the sign of D. The bottom panels of Supplementary

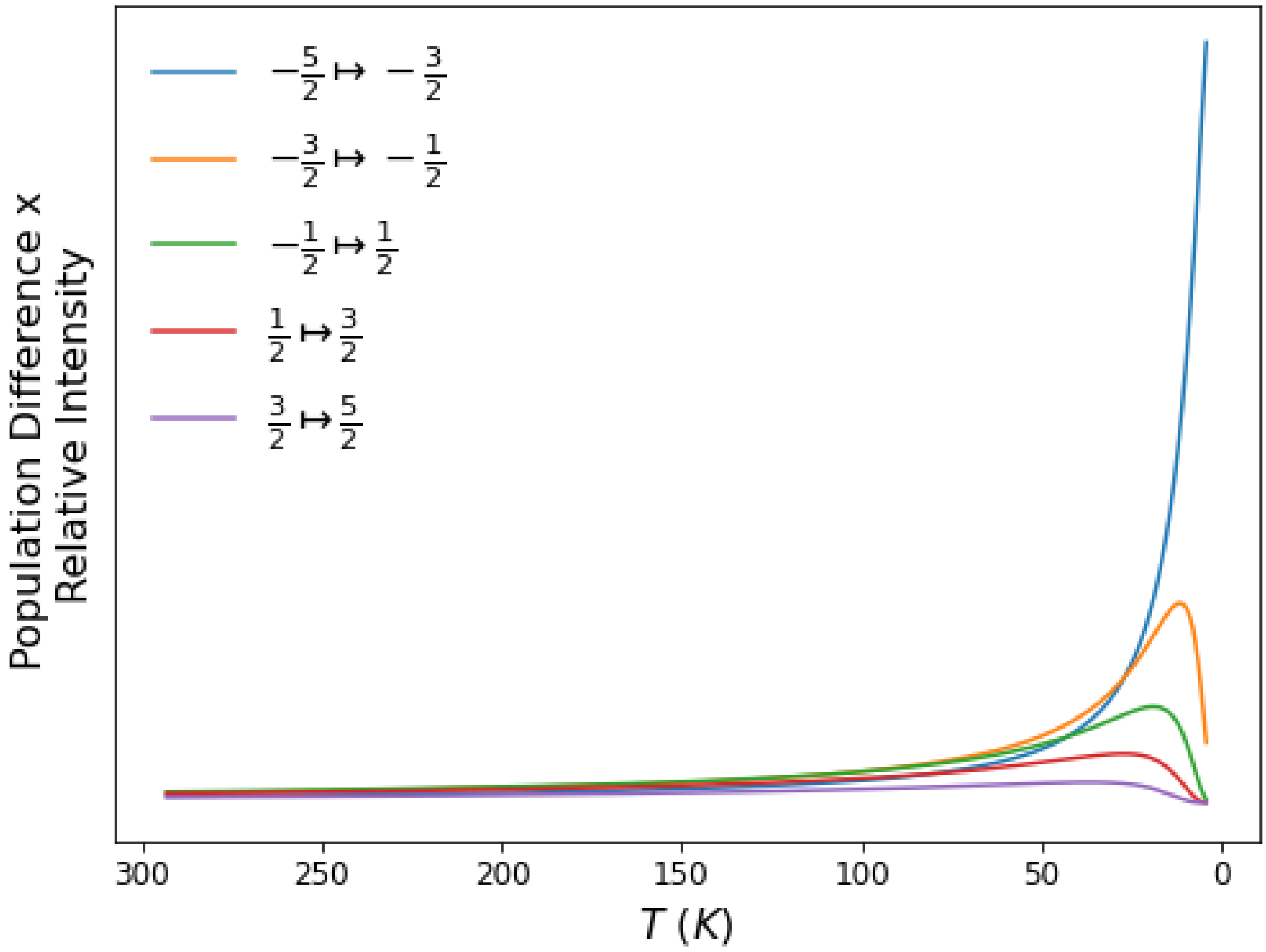

Supplementary Figure 19: Intensity of different Mn EPR transitions as a function of temperature. Calculation assumes $g = 2$ and 263 GHz.

Figure 20 show the case where $D < 0$. Following the same logic, we also see that $D < 0$ causes asymmetry, but in the opposite direction of the $D > 0$ case. Overall, the asymmetry of the Mn spectrum enables direct determination of D and its sign.

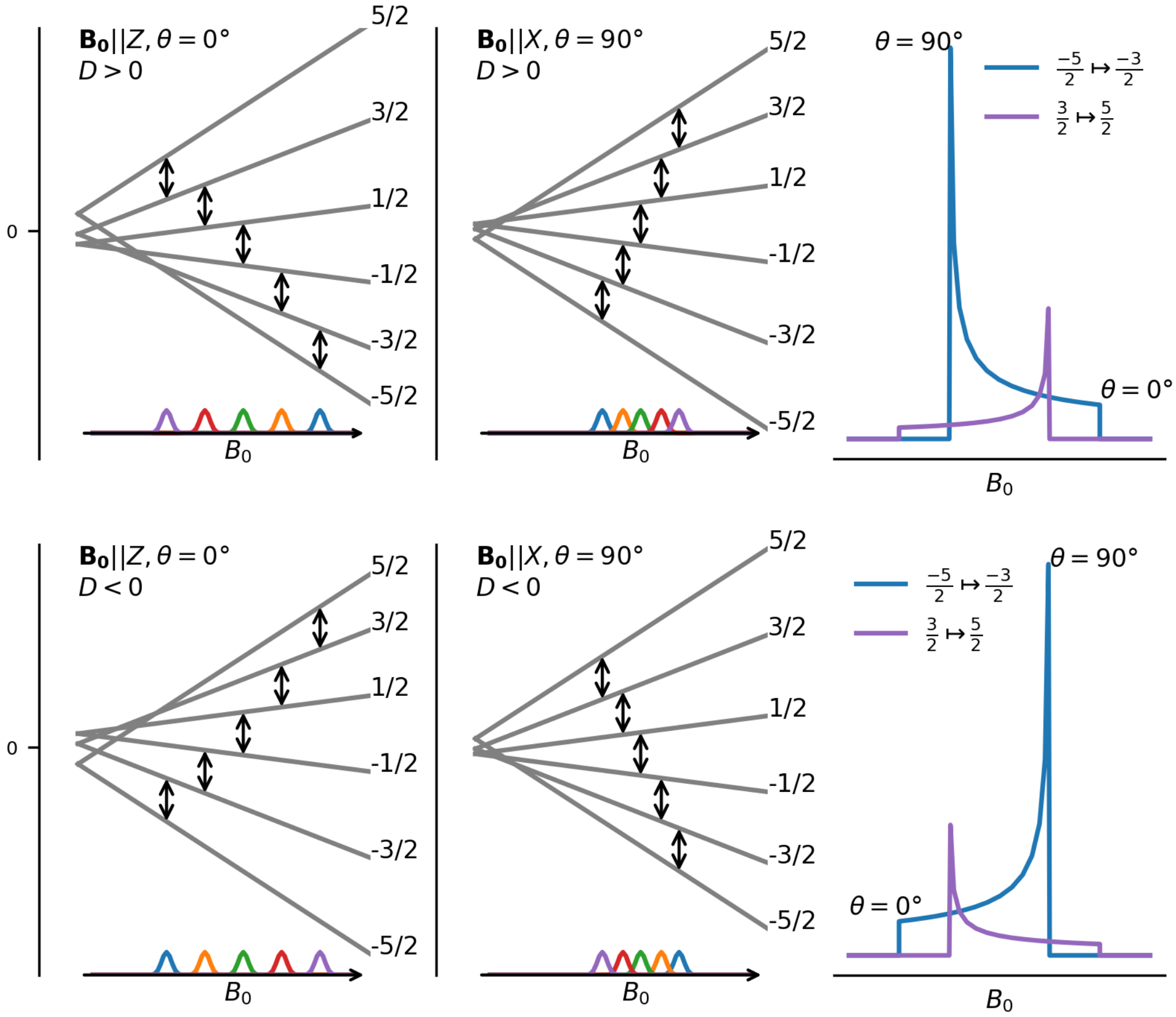

Supplementary Figure 20: Left: Energy diagram of Mn considering only ZFS and Zeeman interactions when $\mathbf{B_0}||Z$. Middle: Energy diagram of Mn considering only ZFS and Zeeman interactions when $\mathbf{B_0}||X$. Right: Spectral shapes of $-5/2 \mapsto -3/2$ and $3/2 \mapsto 5/2$ transitions for a powder sample with linewidth of 0. Two cases are considered for $D > 0$ in the top panels and $D < 0$ in the bottom panels.

3.4 263 GHz Mn(II) spectrum: Sensitivity comparison with and without 10 W TWT

We perform echo-detected field sweep experiments on Mn in MgO to quantify the signal enhancement from the 10 W TWT. Supplementary Figure 21 shows the echo-detected spectra of Mn in MgO collected with and without 10 W TWT. The S/N are calculated by taking the intensity of the Mn peaks divided by the standard deviation of the noise. The S/N is reported as the mean $\pm$ standard deviation of the 6 Mn peaks. Overall, we achieve an almost 9-fold increase in signal intensity due to the 10 W TWT.

We note several nuances that lead to the 9-fold gain. From the combination of the waveguide, the QO components, and probe, we estimate an overall 2.2 W of power at the sample space when we use the 10 W TWT. On the other hand, when using the low-power (0.2 W) setup, we were able to set the microwave source much closer to the QO-components and the magnet since the VDI source is not sensitive to the stray fields when operating at 9.4 T. Hence, the loss we expect from this low-power setup only comes from the QO-components and the probe. Hence, with the low-power setup, we estimate 0.1 W of power at the sample space. Consequently, even with the additional loss from the long waveguide, the high-power (10 W) setup delivers 20-fold more power than the low-power setup.

The difference in power reflects the choice of pulse lengths used for the Mn spectrum. The low-power (0.2 W) Mn spectrum was collected using a $\pi/2$-τ-π of 130 ns-760 ns-260 ns. The pulses are 4.33-fold longer than those used with the 10 W TWT (π-pulse of 60 ns). The longer pulses also require longer τ to mitigate the overlapping of the pulse-bleedthrough and the broader echo. This longer τ further reduces

the expected echo signal due to the phase-memory time relaxation. Overall, the combination of longer pulses and τ lead to the 9-fold weaker signal than the signal collected with the 10-W TWT.

With short pulses from the 10 W TWT, we can estimate the limit of detection for Mn(II). At 100 K, we can detect as low as $3.6*10^{13}$ Mn(II) spins with an S/N of 10. However, we must note that extrapolating our sensitivities with other samples is not straightforward as the effects of relaxation, temperature, pulse lengths, and spectral shape contribute to the limit of detection.

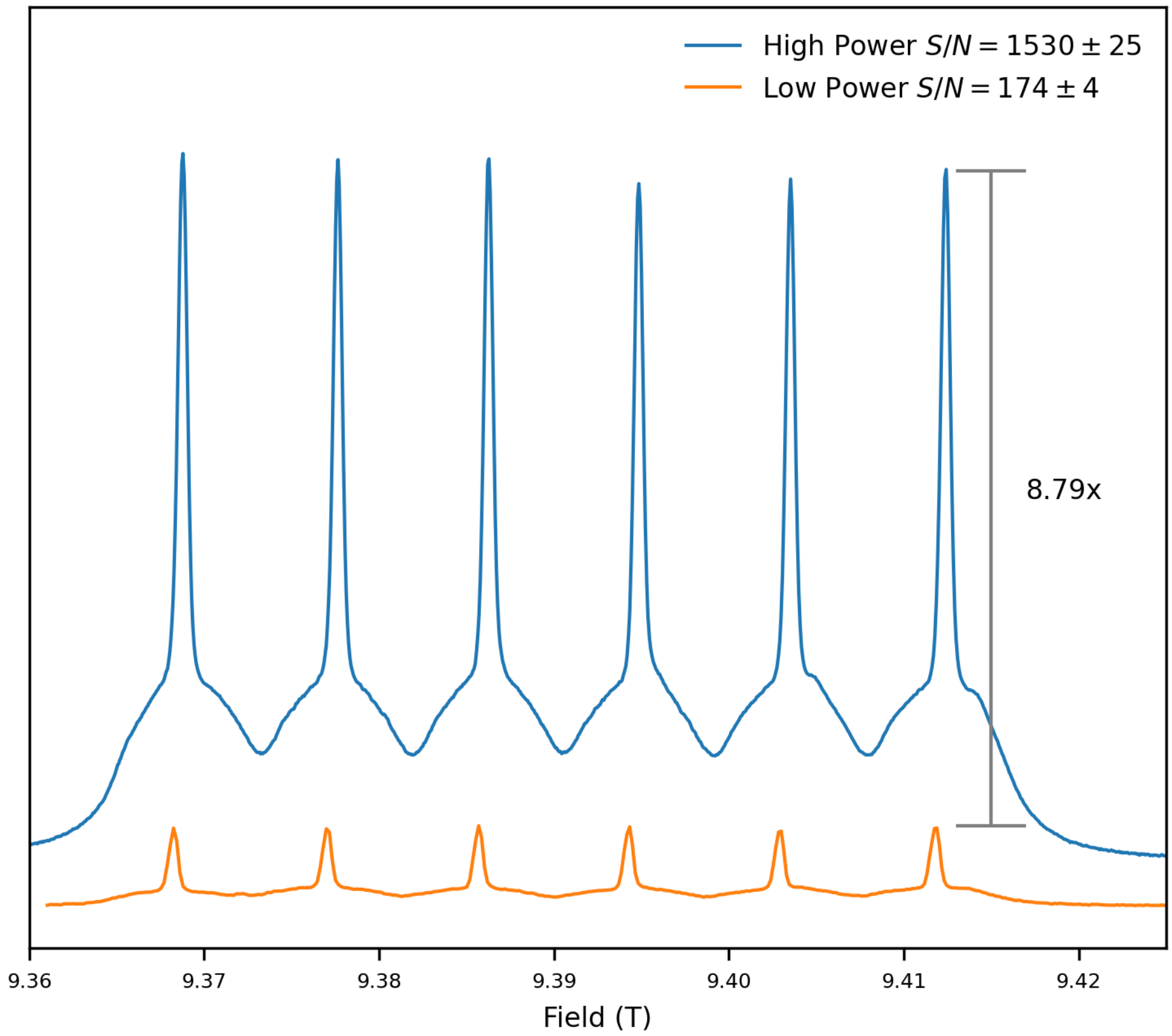

Supplementary Figure 21: Comparison of the Mn in MgO spectra collected with either 0.2 W (without TWT, orange) or 10 W (with TWT, blue) pulses. The two spectra are staggered for visualization. The 10 W pulses lead to about a 9-fold increase in sensitivity.

3.5 Sensitivity comparison with Q-band Mn(II) spectrum

We also perform an echo-detected field sweep experiment on Mn in MgO at Q-band to compare the sensitivity gain between Q-band and 263 GHz. Supplementary Figure 22 shows the spectrum of the MgO sample at Q-band. The S/N achieved is 1927, slightly higher than the 1540 at 263 GHz. The Q-band sensitivity gain reasonably agrees with a previous report of 13-fold increase from X-band [16]. Overall, the gain in sensitivity at 263 GHz is not as high as the gain from Q-band. However, we note that this comparison is based on equal volumes of samples across the two frequencies and thus describes only the absolute spin sensitivity.

The 20 mg of MgO corresponds to 2/3 of the active volume of the Q-band ER 5106-QT2 resonator from Bruker. On the other hand, at 263 GHz, 20 mg of MgO only fills 2/15 of the active volume where the applied magnetic field is constant. Here, because the 263 GHz spectrometer does not rely on a resonator, the volume limitation is much larger than at Q-band. Hence, the relative sensitivity at 263 GHz is still higher than at Q-band, due to the ability to load more spins into the sample space. The

results presented in this work show only the initial sensitivity gain with the 263 GHz spectrometer and the 10W-TWT, while future upgrades to the rest of the spectrometer offer exciting potential for much higher sensitivity gains.

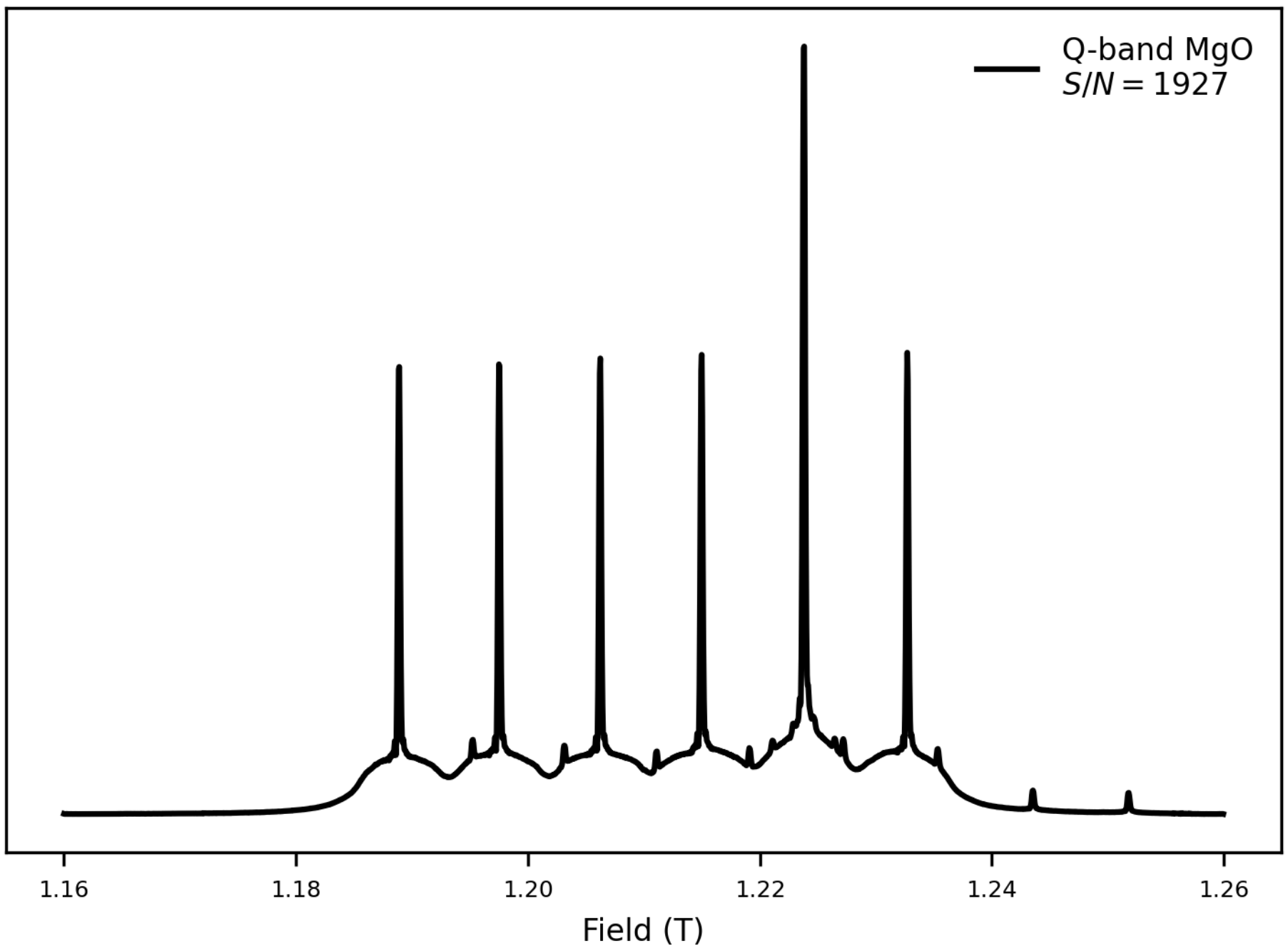

Supplementary Figure 22: Echo-detected spectrum of 20 mg MgO at Q-band, 100 K. Experimental details are described in Supplementary Material Section 1.2.

3.6 Metal assignments of EPR signals in MgO at 263 GHz

The spin state of an EPR signal can be determined by Rabi nutation experiments. Specifically, the optimum π-pulse depends on the electron spin number, S, and the electron transition $|m_s\rangle \rightarrow |m_s'\rangle$, of the sample. The frequency of the Rabi nutation in relation to the spin states is as follows [17]:

$$\omega = \sqrt{S(S+1) - m_s(m_s')}\frac{g\beta_e B_1}{\hbar} \tag{14}$$

where m_s and m_s' are the electron spin states for a $\Delta m_s = \pm 1$ transition, g is the g-value of the spin, β_e is the electron bohr magneton, $\hbar$ is the reduced Planck's constant, and B_1 is the magnetic field component of the pulse. More importantly, we can use the π rotation in the Rabi nutation relative to OX071 (S=1/2) to determine S for the different signals in MgO, shown in Supplementary Figure 23.

The top-left panel shows the different MgO EPR signals, each marked at a different position. Note that we do not observe a signal from Fe, which is the most abundant contaminant in our MgO sample, as listed in Supplementary Table 1. Fe in MgO is expected to be S=2 with large ZFS in the range of 10 cm^{-1} [18, 19], difficult to study with HFEPR alone. Previous work showed that S=2 Fe was observed at 5-6 T using CW HFEPR at 4.5 K [20], but required Frequency-Domain Magnetic Resonance Spectroscopy to resolve the ZFS. So far, the Fe signal has not appeared in our data. However, the search for the Fe signal will be part of future exploration.

The signal at A comes from Mn(II), which we can use as an internal reference for Rabi nutations within the MgO sample. Rabi nutation at A (top right panel) shows a π rotation at 60 ns. Solving for ω with $m_s = -1/2, S = 5/2$, we expect the π rotation to be 3 times faster than the 200 ns π rotation of OX071. Hence, the 60 ns π rotation at A is in reasonable agreement with the Mn(II) assignment.

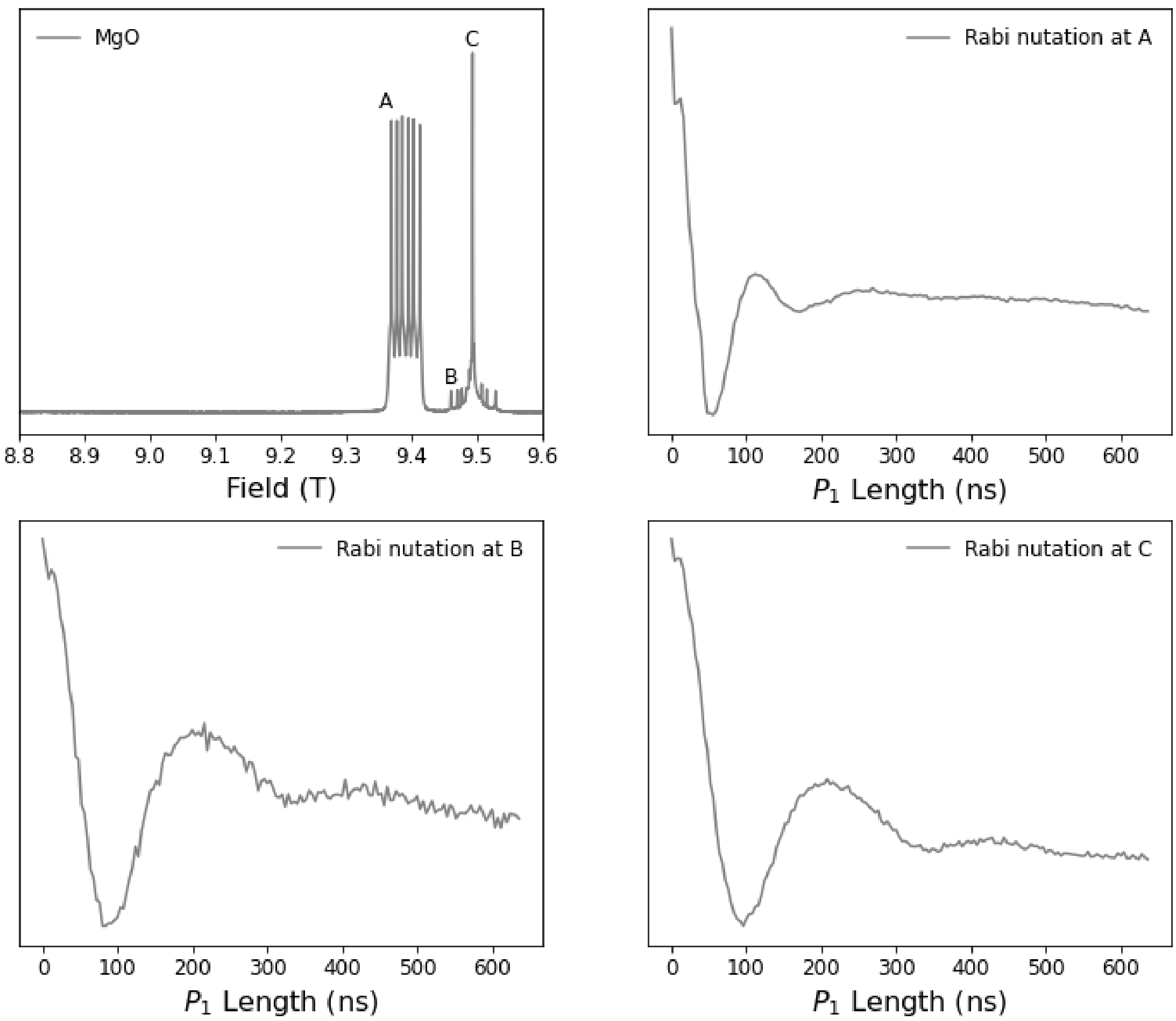

Supplementary Figure 23: Top Left: Field-Swept Spectrum of MgO powder at 100 K. Different EPR signals are labeled to indicate positions for different Rabi nutation experiments. Top Right: Rabi nutation at the sharp Mn(II), S=5/2 signal at (A). Bottom Left: Rabi nutation at the sharp satellite signal at (B). Bottom Right: Rabi nutation at the sharp major signal at (C). Both B and C indicate that the EPR signals are from spins of S=3/2.

With OX071 and Mn(II) as our π rotation references, we can further use Rabi nutations at B (bottom left) and C (bottom right) to further identify the other EPR signals in MgO. Both B and C show a similar π rotation of 100 ns, corresponding to S=3/2. Given the possible spin states of the metals detected in ICP-MS (Table 1), we can assign C as Cr(III) due to its singular peak at g=1.98 with a nuclear spin of I=0, which matches with previously published Cr(III) in MgO spectrum at X-band [21]. Additionally, the shoulder feature at the base of g=1.98 likely comes from Chromium-53 (^{53}Cr) with I=3/2 (9.5% natural abundance). On the other hand, the signal at B is part of an 8-peak EPR signal, likely coming from an I=7/2. Hence, the signal can come from V(II) (S=3/2, I=7/2) or Co(II) (S=3/2, I=7/2). However, V(II) has been observed at g=1.98 in MgO [22], which better supports the assignment of V(II), rather than Co(II), to the 8-peak signal. To further eliminate Co(II) as a possibility, we performed X-band CW EPR on the MgO sample.

Supplementary Figure 24 shows the X-band CW of the MgO powder at 10 K, where typical HS Co(II) peaks appear. The signal appears at g_{eff} of 4.27, as depicted in the bottom simulation with an S=1/2. The measured g_{eff} is in agreement with the published value of g=4.25 [23]. We further simulated the data with S=3/2 and obtained g as 2.147 with a D of 30 cm^{-1}. We note that the accuracy of g and the D parameter is limited at X-band. However, the g used in our simulation falls within the typical values

for Co(II), which are generally $g > 2$ across different coordination numbers [24]. Hence, the signal at 1.98 observed in the 263 GHz spectrum is unlikely to be Co(II). In general, we assign the three EPR signals in the MgO spectrum at 263 GHz as ^{55}Mn(II), ^{52}Cr(III), and ^{51}V(II). Simulation of the assignments are shown in Supplementary Figure 25. However, we note that this assignment is tentative until further confirmation using specific control samples.

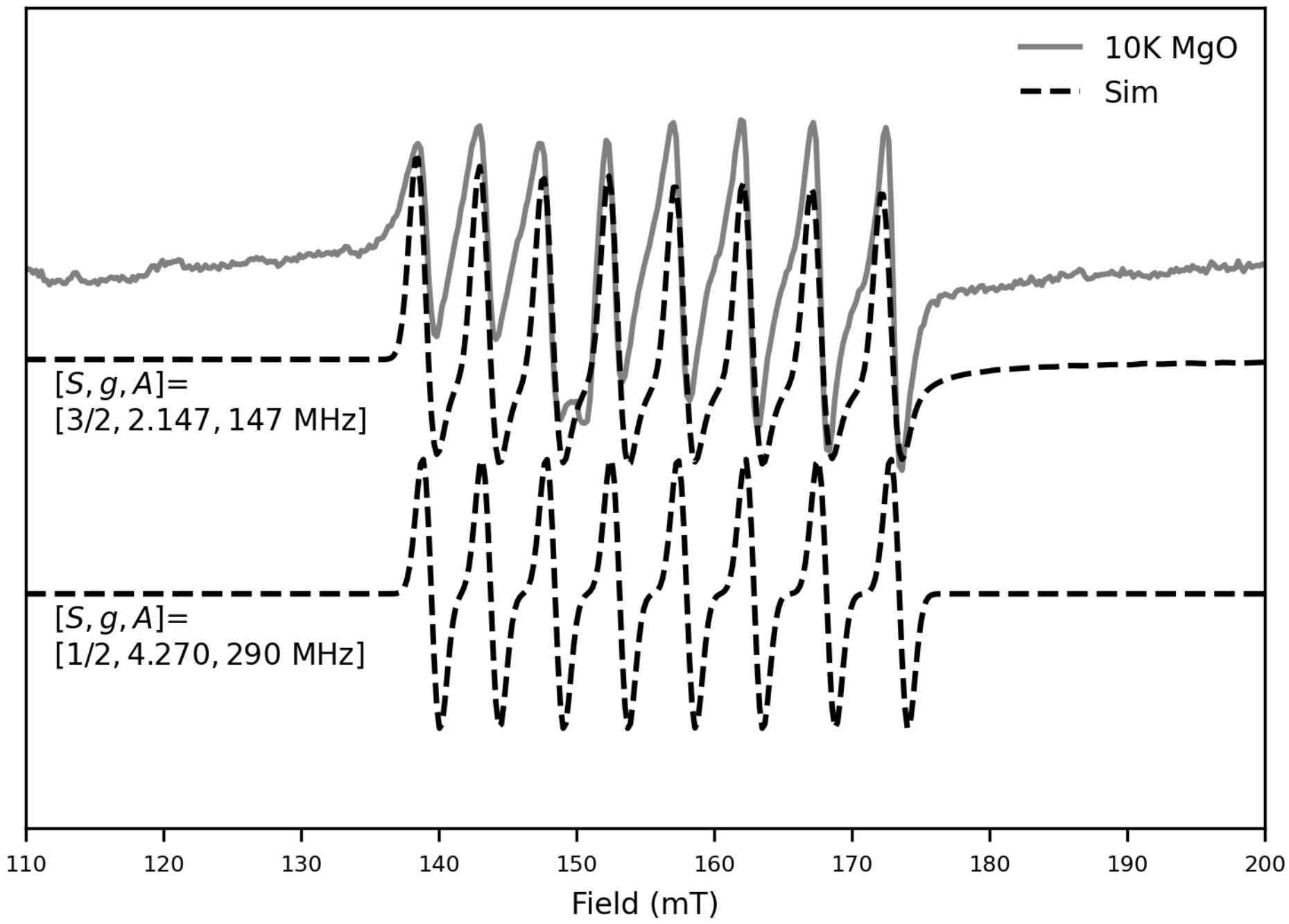

Supplementary Figure 24: X-band CW of MgO in the low field region where HS Co(II) signal appears at T=10 K. Simulation was done either using $S_{eff} = 1/2$, $g_{eff} = 4.27$, $A = 290 MHz$ (bottom simulation) or $S = 3/2$, $g = 2.147$, $A = 147 MHz$, and $D = 30 cm^{-1}$ (top simulation).

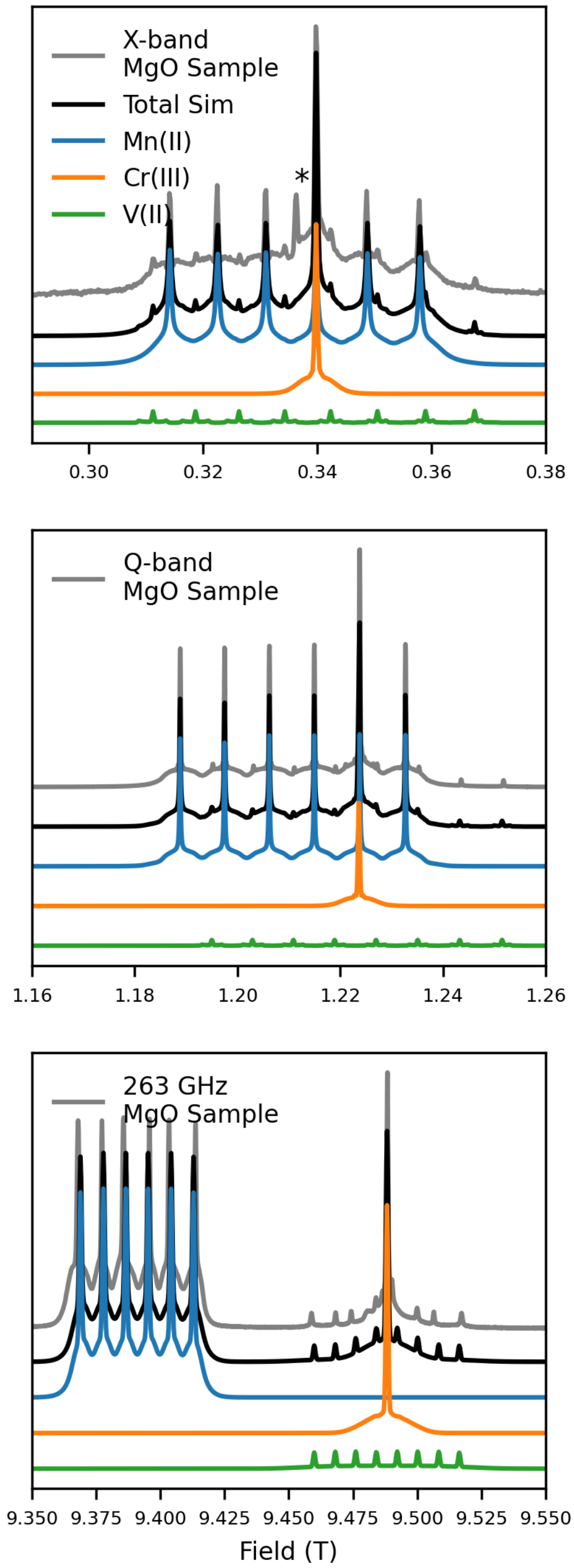

Supplementary Figure 25: Top: X-band echo-detected Field-Swept spectrum of MgO. Unlike the X-band spectrum in the main manuscript, this data was collected with 1024 shots to further resolve the V(II) signal. Middle: Q-band echo-detected Field-Swept spectrum of MgO. Bottom: 263 GHz echo-detected Field-Swept spectrum of MgO. Spectra were collected at 100 K. Simulations were done using the following parameters: ^{55}Mn(II) (S=5/2, g=2.0009, A=245.6 MHz, D=-50 MHz), Cr(III) (S=3/2, g=1.9804, A=15 MHz, D=-50 MHz), and ^{51}V(II) (S=3/2, g=1.9804, A=223.5 MHz). The use of A for Cr(III) is to fit the shoulder feature at the base of g=1.98 that comes from Chromium-53 (^{53}Cr) that has I=3/2 (9.5% natural abundance).

References

[1] Schosseler, P., Wacker, T. & Schweiger, A. Pulsed ELDOR detected NMR. *Chemical physics letters* **224**, 319–324 (1994). https://linkinghub.elsevier.com/retrieve/pii/0009261494005486.

[2] Cox, N., Nalepa, A., Lubitz, W. & Savitsky, A. ELDOR-detected NMR: A general and robust method for electron-nuclear hyperfine spectroscopy? *Journal of Magnetic Resonance* **280**, 63–78 (2017). http://dx.doi.org/10.1016/j.jmr.2017.04.006.

[3] Epel, B., Gromov, I., Stoll, S., Schweiger, A. & Goldfarb, D. Spectrometer manager: A versatile control software for pulse epr spectrometers. *Concepts in Magnetic Resonance Part B: Magnetic Resonance Engineering* **26B**, 36–45 (2005). https://doi.org/10.1002/cmr.b.20037.

[4] Smith, A. A. *et al.* A 140 GHz pulsed EPR/212 MHz NMR spectrometer for DNP studies. *Journal of Magnetic Resonance* **223**, 170–179 (2012). http://dx.doi.org/10.1016/j.jmr.2012.07.008.

[5] Oyala, P. H. *et al.* Biophysical characterization of fluorotyrosine probes site-specifically incorporated into enzymes: E. coli ribonucleotide reductase as an example. *Journal of the American Chemical Society* **138**, 7951–7964 (2016). http://dx.doi.org/10.1021/jacs.6b03605.

[6] Cho, F. H., Stepanov, V. & Takahashi, S. A high-frequency electron paramagnetic resonance spectrometer for multi-dimensional, multi-frequency, and multi-phase pulsed measurements. *The Review of scientific instruments* **85**, 075110 (2014). http://dx.doi.org/10.1063/1.4889873.

[7] Takahashi, S. *et al.* Pulsed electron paramagnetic resonance spectroscopy powered by a free-electron laser. *Nature* **489**, 409–413 (2012). http://dx.doi.org/10.1038/nature11437.

[8] Cruickshank, P. A. S. *et al.* A kilowatt pulsed 94 GHz electron paramagnetic resonance spectrometer with high concentration sensitivity, high instantaneous bandwidth, and low dead time. *The Review of scientific instruments* **80**, 103102 (2009). http://dx.doi.org/10.1063/1.3239402.

[9] Nanni, E. A., Jawla, S. K., Shapiro, M. A., Woskov, P. P. & Temkin, R. J. Low-loss transmission lines for high-power terahertz radiation. *Journal of infrared, millimeter and terahertz waves* **33**, 695–714 (2012). http://dx.doi.org/10.1007/s10762-012-9870-5.

[10] Goldsmith, P. F. *Quasioptical Systems* (IEEE, 1998). https://doi.org/10.1109/9780470546291.

[11] Stoll, S. & Schweiger, A. Easyspin, a comprehensive software package for spectral simulation and analysis in epr. *Journal of Magnetic Resonance* **178**, 42–55 (2006). https://doi.org/10.1016/j.jmr.2005.08.013.

[12] Clayton, J. A. *et al.* Quantitative analysis of zero-field splitting parameter distributions in gd(iii) complexes. *Physical Chemistry Chemical Physics* **20**, 10470–10492 (2018). URL http://dx.doi.org/10.1039/c7cp08507a.

[13] Gromov, I. Novel MM-wave EPR spectrometer ELEXSYS 7th series:design and performance (2015). http://rgdoi.net/10.13140/{RG}.2.1.3306.3525.

[14] Reed, G. H. & Markham, G. D. EPR of mn(II) complexes with enzymes and other proteins. In Berliner, L. J. & Reuben, J. (eds.) *Biological Magnetic Resonance*, 73–142 (Springer US, Boston, MA, 1984). https://doi.org/10.1007/978-1-4615-6546-8_3.

[15] Pake, G. E. & Estle, T. L. *The Physical Principles of Electron Paramagnetic Resonance* (Reading, Mass., W.A. Benjamin, Advanced Book Program, 1973), 2nd edn.

[16] Ghimire, H., McCarrick, R. M., Budil, D. E. & Lorigan, G. A. Significantly improved sensitivity of q-band PELDOR/DEER experiments relative to x-band is observed in measuring the intercoil distance of a leucine zipper motif peptide (GCN4-LZ). *Biochemistry* **48**, 5782–5784 (2009). URL http://dx.doi.org/10.1021/bi900781u.

[17] Astashkin, A. & Schweiger, A. Electron-spin transient nutation: a new approach to simplify the interpretation of ESR spectra. *Chemical physics letters* **174**, 595–602 (1990). https://doi.org/10.1016/0009-2614(90)85493-V.

[18] Abragam, A. & Bleaney, B. *Electron Paramagnetic Resonance of Transition Ions* (Oxford University Press Academic UK, 2012), illustrated edn.

[19] Krzystek, J., Ozarowski, A. & Telser, J. Multi-frequency, high-field EPR as a powerful tool to accurately determine zero-field splitting in high-spin transition metal coordination complexes. *Coordination chemistry reviews* **250**, 2308–2324 (2006). URL https://linkinghub.elsevier.com/retrieve/pii/S0010854506000737.

[20] Telser, J. *et al.* High-frequency/high-field EPR spectroscopy of the high-spin ferrous ion in hexaaqua

complexes. *Magnetic Resonance in Chemistry* **43 Spec no.**, S130–9 (2005). URL http://dx.doi.org/10.1002/mrc.1689.

[21] Low, W. Paramagnetic resonance and optical absorption spectra of cr^{3+} in mgo. *Phys. Rev.* **105**, 801–805 (1957). https://doi.org/10.1103/PhysRev.105.801.

[22] Low, W. Paramagnetic resonance spectra of some ions of the $3d$ and $4f$ shells in cubic crystalline fields. *Phys. Rev.* **101**, 1827–1828 (1956). https://doi.org/10.1103/PhysRev.101.1827.

[23] Schwab, T., Niedermaier, M., Zickler, G. A., Ončák, M. & Diwald, O. Isolated cobalt ions embedded in magnesium oxide nanostructures: Spectroscopic properties and redox activity. *Chemistry – A European Journal* **26**, 16049–16056 (2020). https://doi.org/10.1002/chem.202002817.

[24] Tripathi, S., Dey, A., Shanmugam, M., Narayanan, R. S. & Chandrasekhar, V. *Cobalt(II) Complexes as Single-Ion Magnets*, 35–75 (Springer International Publishing, Cham, 2019). https://doi.org/10.1007/3418_2018_8.
