# Supplementary material for: Very high frequency (263 GHz) pulse EPR spectroscopy of high spin transition metal centers
Source: Nat Commun. 2026 Oct 1;17:11275. doi: 10.1038/s41467-026-78076-w (PMC13720073; doi:10.1038/s41467-026-78076-w)
Supplement: Supplementary file 2 [file 41467_2026_78076_MOESM2_ESM.pdf]

nature portfolio

Peer Review File

Very high frequency (263 GHz) pulse EPR spectroscopy of high spin transition metal centers

Corresponding Author: Professor R. David Britt

This file contains all reviewer reports in order by version, followed by all author rebuttals in order by version.

Version 0:

Reviewer comments:

Reviewer #1

(Remarks to the Author)
This manuscript reports on a substantial advancement in high-field electron paramagnetic resonance (EPR) instrumentation that has a potential for wide application across several scientific disciplines. Therefore, it is of sufficiently broad interest for Nature Communications. The performance of the instrument is impressive, the results are of high quality, and the main conclusions are supported by experimental evidence. The manuscript is concise and, in general, well written. However, some aspects of the presentation could be improved and the Supplementary Material (SM) contains a few mistakes. I recommend minor revision.

Details:

1. Figure 1 indicates a relatively long ringing time, especially considering that no resonator is used. Please discuss this at least shortly. Is this due to reflections? Would a design as the one in St Andrews at 94 GHz be expected to improve this? Although sensitivity is quite good, it might be even better if this problem could be solved.

2. In a way, high-spin species such as Mn(II) are an easy target. They are a sufficiently large field to make the spectrometer widely applicable, but the authors should be more upfront about the microwave field amplitude being too small for most applications that would be of interest for S = ½ species. Currently, readers only recognize this if they are experts in the field or at least read the SM.

3. Due to the line narrowing at higher fields, high-spin Kramers ions also exhibit higher sensitivity advantage than other paramagnetic species. This must be mentioned. I expect that the authors did already compare sensitivity for the trityl sample (used in the SM for determining microwave field amplitude) between the commercial X-band spectrometer and the instrument described here. The result should be reported. If this has not yet been done, this experiment needs to be performed. Even if the title explicitly mentions high spin metal centres, the readers should be informed that the instrument is (probably) less attractive for organic radicals.

4. The different population of the different states for a high spin metal ion (discussed in the SM) allows for determination of the sign of the zero-field splitting from the asymmetry (more intensity low field than high field or vice versa). This should be mentioned. Apparently, the authors even have examples with different sign (EDTA and the PsaA complex). The signs should be given for these cases.

5. The authors mention EDNMR as a potential application. How likely is it that this will work out? Transition moments of formally forbidden transitions are lower at higher field and the transition Rabi frequency for the allowed transitions is substantially lower than for W-band spectrometers capable of EDNMR. At least this caveat should be mentioned.

6. Please point out in the main text that the Cr(III) signal is also visible at X-band. If the Mn(II):MgO sample is similar to the one in my lab, I believe that the putative V(II) signal can also be seen with CW EPR at X-band frequencies. The authors should check that and if so, mention this.

7. In the main text, the spin state for V(II) and Co(II) should be mentioned.

8. In Fig. 4, please mention that the temperature is 100 K as well for the X-band experiment.

9. In the SM, the probehead is called a cryoprobe. This is misleading. The term cryoprobe is used in NMR for probes with a cryogenic low-noise amplifier. Please avoid using this established term for something else.

10. In the SM in the equation below line 145 a spin operator is missing.

11. In the SM, all equations on p. 18 mix a Hamilton operator with energy eigenvalues.

Typos:

l. 255: “or or” delete one “or”

Reviewer #2

(Remarks to the Author)
This paper reports the application to Mn(II) of the 263 GHz amplifier that was described in the IEEE Transactions in 2023. Significant advantages for the study of high-spin metal systems are demonstrated at each increase in EPR microwave frequency and magnetic field. Direct comparison of the performance on the new spectrometer with the commercially available 263 GHz spectrometer is provided, with explanation of the differences.

The parts list and the photographs of the spectrometer in the supplementary materials are crucial to understanding the paper.

There will be general interest in the paper and it should be published.

Minor revision in accordance with the following comments will enhance the paper.

The better signal-to-noise achieved with the new spectrometer relative to the Bruker spectrometer is well-demonstrated, but it is also necessary to discuss the capabilities that were given up in the new design. One key feature is that the temperature and atmospheric environment of the sample cannot be controlled while the sample is loaded into the holder (figure S14). This eliminates use with many biological systems for which the high frequency would be desired. However the text in line 213 refers to an “Open 5 mm diameter sample.” Please provide a corresponding picture of how the 5 mm sample can be used. Why “open”? Is this what is referred to separately as a teflon cup? Overall, clarification of limitations on sample geometry and handling is essential.

In figure 6 very little of the Mn(II) intensity is in the 6 sharp lines. The dotted lines show how the overall pattern is a sum, but have the field-swept spectra ben acquired at different pulse powers to optimize for each different transition to substantiate the assignments? Since the focus of the paper is the information that one can acquire about Mn(II) at 263 GHz, more discussion of these spectra is expected.

Given the high Fe concentration in the MgO sample (Supplementary Table 1), why is no Fe EPR signal observed?

It is appropriate that the paper state that the Mn in MgO sample that they used is no longer available from the commercial source, but it would be useful to state that almost any commercial CaO (and probably many other divalent oxides) has a Mn(II) EPR signal that could be used instead.

Figure 4 caption should explain the high-intensity signal.

Reviewer #3

(Remarks to the Author)
This manuscript presents a significant instrumental development in pulse high field EPR, 263 GHz, featuring high power (10 W), which is exceptional at these frequencies. This is coupled with a bandwidth of 5.6 GHz of the power amplifier and a resonator free design that allows for a 12 l sample size. The construction and design of the power amplifier (already published) and the spectrometer are impressive and novel and open many new possibilities. The properties of this new spectrometer are demonstrated on rather standard high-spin Mn(II) (S=5/2) complexes and two Mn(II) containing proteins. The motivation is the well-known fact that linewidth of the central transition is inversely proportional to the spectrometer frequency, which increases sensitivity and resolution. While the prospects of this new spectrometer are excellent, none of these are demonstrated in the work. Therefore, this work, which is mainly instrumental in character, should be published in journals like Rev. Sci. Instruments or JMR or MR.
Below we list concerns and comments that should be addressed before publication in any journal.
1. The authors demonstrate the performance of the spectrometer on trivial samples and the simplest pulse EPR experiment, Echo-detected EPR, that yields the EPR spectrum, which can be obtained with CW-spectrometers as well. To convince the reader that this new spectrometer indeed stands out to its promise the authors should show pulse EPR results that emphasize the prospect of the high power and band width that are hard to achieve with existing spectrometers (W-band and higher) in terms of resolution.

2. To show the “breakthrough” nature of the new instrument the authors use a comparison with X-band spectrometers. This is an obsolete comparison. The comparison should be done with state of the art commercial Q-band spectrometers that are widely available and more importantly with 95 GHz spectrometers (homebuilt and commercial) , which have been around for a long time and reported a plethora of advanced pulse EPR applications such as ENDOR, DEER, HYSCORE, ELDOR-detected NMR etc, applied to both S=1/2 and high-spin systems (none of this is referenced). The information derived from the echo-detected EPR examples shown in this work is easily obtained at W-band or with the 263 GHz Bruker spectrometer.
3. The new spectrometer indeed allows relatively short /2 pulses for Mn(II), 30 ns, because of its high spin nature. The pulse length for S=1/2 is longer, 100 ns, which is not that attractive. A Bruker 263 GHz spectrometer is commercially available and uses a resonator which limits the sample size and band width. Nevertheless, it affords a high B1 that yields a /2 pulse of 30 ns for S=1/2 . Very nice pulse ENDOR measurements were reported with this spectrometer (Bennati’s lab)
4. There is no discussion about sensitivity limits. Please add.

Other minor issues:
1. Is phase cycling implemented, how ?
2. What is the deadtime of the spectrometer?
3. The bandwidth of the amplifier is given, but not of the full bridge.
4. What is the cooling system and what is the lowest possible temperature?

Reviewer #4

(Remarks to the Author)
I co-reviewed this manuscript with one of the reviewers who provided the listed reports. This is part of the Nature Communications initiative to facilitate training in peer review and to provide appropriate recognition for Early Career Researchers who co-review manuscripts.

Version 1:

Reviewer comments:

Reviewer #1

(Remarks to the Author)
The authors have revised the manuscript in a satisfying way, addressing my main concerns. I agree with the arguments that the achieved advancement is substantial and justifies publication in Nature Communications (as I already recommended for the first version). I now recommend publication of the manuscript in its present form.

Reviewer #2

(Remarks to the Author)
the authors responded well to the comments of all reviewers.
the revised paper and supplementary materials are ready for publication.

Please fix the spelling of P. Hofer in reference 10.

Reviewer #3

(Remarks to the Author)
We appreciate the authors’ efforts in revising the manuscript and responding to our comments. While several minor technical issues have been addressed, the authors have disagreed with our concerns and have chosen not to revise the manuscript accordingly. Although they have provided lengthy explanations of their position, we do not find them sufficiently compelling to alter our overall assessment of the manuscript. Consequently, our evaluation remains unchanged. We also note that discussions of plans and the status of related grant applications are relevant to the current manuscript.
1. We agree that the prospects of such a high-power spectrometer are promising and impactful. However, for a publication in a high-impact, broad-scope journal, we would expect at least one clear, convincing example (sample or experiment, not a standard sample) demonstrating the combined advantages of high frequency and high power over existing spectrometers.
2. When presenting a newly developed, state-of-the-art spectrometer, it is important to benchmark its performance against existing leading instruments. In this case, a comparison with high-frequency pulsed spectrometers (e.g., 95, 140, 180, 195, 263, or 390 GHz systems) would be more appropriate. Comparisons limited to low- and medium-frequency pulsed X- and Q-band spectrometers may not fully capture the advancement, even if those systems are widely available. While we understand that such comparisons may be planned for future work, their absence in the current manuscript weakens the conclusions.

Reviewer #4

(Remarks to the Author)
I co-reviewed this manuscript with one of the reviewers who provided the listed reports. This is part of the Nature Communications initiative to facilitate training in peer review and to provide appropriate recognition for Early Career Researchers who co-review manuscripts.

Version 2:

Reviewer comments:

Reviewer #2

(Remarks to the Author)
The modifications to comparison of other EPR spectrometers in the latest revision is at an appropriate level of detail. When more demonstrations of spectra and relaxation times are produced we can expect even more impressive comparison of the capabilities of this instrument relative to others.

Reviewer #3

(Remarks to the Author)
There were two points of concern in our last review. The authors made an effort to address point (2), which are text changes. With respect to point (1), unfortunately, it seems that the authors do not have examples demonstrating the combined advantages of their high frequency and high-power spectrometer over existing spectrometers. Hopefully, they will have this in the future. We have no objection to publishing this manuscript in your journal.

Reviewer #4

(Remarks to the Author)
I co-reviewed this manuscript with one of the reviewers who provided the listed reports. This is part of the Nature Communications initiative to facilitate training in peer review and to provide appropriate recognition for Early Career Researchers who co-review manuscripts.

Open Access This Peer Review File is licensed under a Creative Commons Attribution 4.0 International License, which permits use, sharing, adaptation, distribution and reproduction in any medium or format, as long as you give appropriate credit to the original author(s) and the source, provide a link to the Creative Commons license, and indicate if changes were made.
In cases where reviewers are anonymous, credit should be given to 'Anonymous Referee' and the source.
The images or other third party material in this Peer Review File are included in the article's Creative Commons license, unless indicated otherwise in a credit line to the material. If material is not included in the article's Creative Commons license and your intended use is not permitted by statutory regulation or exceeds the permitted use, you will need to obtain permission directly from the copyright holder.
To view a copy of this license, visit https://creativecommons.org/licenses/by/4.0/

REVIEWER COMMENTS and our responses

As an overview to our response to reviews, before the point-by-point discussion, we emphasize that the focus of this manuscript is to show how new mm-wave pulse amplifier technology, as described in a recent paper [1], gives unique access to high power pulse EPR spectroscopy at significantly higher frequencies/fields and with much greater frequency bandwidth than has existed before. Slow wave vacuum tube amplifiers are the de-facto standard for X band (~9.5 GHz) pulse EPR spectrometers. The traveling wave tube (TWT) vacuum tube design works very well at these relatively low microwave frequencies. (For example, the Applied Systems Engineering 8-18 GHz TWT we bought in 1989 for our first UC Davis pulse EPR still works fine.) But for the TWT vacuum electronics amplifier, the internal active workings must be about 30X smaller at 263 GHz, which makes fabrication a big challenge as the path the high voltage electrons must clear within the amplifier is now very small.

Here we demonstrate new gains in pulse EPR at much higher frequency using this unique new TWT designed by my late UC Davis engineering colleague Neville Luhmann Jr and his group members. The 10 W TWT design and performance was published in 2023 [1], and it is now integrated into our 263 GHz quasioptics bridge EPR spectrometer as a first implementation of high power amplification for pulse EPR at such a high frequency. The preprint of this Nature Communications manuscript has already generated much attention, including from companies who want to partner with us for even higher power implementations, so clearly this work is having an impact in the pulse EPR/DNP community, including commercial operators. In particular, DNP (dynamic nuclear polarization) is typically carried out with single frequency continuous wave gyrotrons for the electron spin resonance, which limits its performance, particularly at higher frequencies. We now have a frequency agile amplifier that can drive time domain DNP, which is recognized as an important development in the community. In fact, we have two proposals submitted to implement 400 MHz DNP (which matches the 263 GHz EPR frequency) based on this platform, and we are partnered with the new Pines Magnetic Resonance Center in Berkeley to work on such future projects. More about time domain DNP has been added in the revision (lines 305-315)

To clarify further, we did not build this instrument to do continuous wave EPR (or DNP). Low power sources are available to much higher frequency than 263 GHz, and we have coauthored a number of such papers over the years, mostly with the National High Magnetic Field Laboratory EMR group. The challenge has been to do high power *pulse* EPR because amplifiers with sufficient power and bandwidth have been lacking above about 95 GHz (W-band). The submitted manuscript demonstrates breakthrough success at this frequency which is approximately three times higher than that of the *few* existing high power W band instruments, with an additional benefit of much higher

bandwidth. Most importantly, this instrument has enough power (10 W) at this high mm-wave frequency to achieve high performance without requiring a resonant cavity or any other form of resonator. Such resonators invariably limit sample volume and flexible sample geometry at such high mm-wave frequencies, given that their dimensions scale inversely with frequency, giving approximately an inverse cube relation as to sample volume and forcing samples to be loaded into tiny capillary tubes. Bruker offers a commercial low power 263 GHz system, and I agree with reviewers that it works well given the necessarily limited sample volumes. However, a crucial point here is that we are demonstrating an alternative approach, which accommodates much larger sample volumes and alternate geometries, and bypasses many of the constraints that come with tiny EPR tubes, such as difficulty of packing trapped intermediates or incorporating electrochemical cells, etc. In my view, alternative design implementations should be considered a major plus in the magnetic resonance community.

This manuscript was submitted when the basic spin echo implementation of 263 GHz pulse instrument was built and its performance characterized targeting an array of high spin transition metal centers. We have a pending NSF proposal under review, which if funded, will allow us to add some of the reviewer-suggested capabilities in the future. This is very much our plan. But we consider the results reported in the original submission adequate for a first *Nature Communications* article, as agreed by reviewers 1 and 2. The results we present nicely match the length of the *Communications* format. Since we do not have the resources of a multinational corporation, it’s step by step for us moving forward, and we are making steady progress based on the breakthrough pulse EPR performance described here, including using the work described in the manuscript as the basis of current grant applications to fund interesting future developments.

It is also important to note that it is the nature of the diverse classes of samples for EPR spectroscopy (including pulse EPR) that no one frequency band is uniquely superior for all applications (lines 292-304). That is why our lab is equipped with many instruments, ranging in frequency from 8 GHz up to now 263 GHz. Some of the details are discussed in the point-by-point discussion of the reviewer comments, which begin here:

Reviewer #1 (Remarks to the Author):

This manuscript reports on a substantial advancement in high-field electron paramagnetic resonance (EPR) instrumentation that has a potential for wide application across several scientific disciplines. Therefore, it is of sufficiently broad interest for Nature Communications. The performance of the instrument is impressive, the results are of high quality, and the main conclusions are supported by experimental evidence. The manuscript is concise and, in general, well written. However, some aspects of the

presentation could be improved and the Supplementary Material (SM) contains a few mistakes. I recommend minor revision.

We thank the reviewer for thoughtful opinions including concluding that this is a good Nature Communications manuscript, and we have addressed the suggestions, including carefully revising the SM.

Details:

1. Figure 1 indicates a relatively long ringing time, especially considering that no resonator is used. Please discuss this at least shortly. Is this due to reflections? Would a design as the one in St Andrews at 94 GHz be expected to improve this? Although sensitivity is quite good, it might be even better if this problem could be solved.

Based on a Mark Sherwin paper [2] *(see figure 4), we assign this effect to some reflections from the waveguide and the quasioptics as opposed to cavity ringdown (as noted we indeed have no cavity/resonator). We note that movement of our reflector plate and the mirrors in practice affects this pulse bleedthrough. The St Andrews setup [3] does seem to have more control of the reflector plate to minimize stray cross-polar reflections, and we can work towards this as the project development continues to move forward. We also plan to soon implement Hahn echo phase cycling to be able to actively subtract some of this bleedthrough. At lower frequency, such as X- band, we could "blank" this pre-echo bleedthrough, but at 263 GHz such solid state switches have too much insertion loss and we do not wish to sacrifice actual signal intensity. These are future improvements to be made (and as we noted above we are actively working towards grant support for future improvements of the instrument). It is worth nothing some such "improvements" may affect the overall simplicity of our current system, including our ability to utilize the large sample space as discussed. Ultimately, we may employ different probe designs for different applications: it is easy to swap out the bottom section of the probe so this could be an effective strategy. But for the moment we are happy to exploit the simple "no resonator/large versatile sample space" mode that we demonstrate good performance within this manuscript. But in the long run we will likely explore other operating modes as well.*

2. In a way, high-spin species such as Mn(II) are an easy target. They are a sufficiently large field to make the spectrometer widely applicable, but the authors should be more upfront about the microwave field amplitude being too small for most applications that would be of interest for S = ½ species. Currently, readers only recognize this if they are experts in the field or at least read the SM.

We agree as to the advantage for high spin ions, as have noted this in our prolog above and have made clear in the revised manuscript. But we do not fully agree with the second conclusion. We have one simple way planned to increase effective B1 field with the current amplifier, which is to replace our somewhat lossy "homemade" corrugated waveguide with lower loss commercial product. Again, this needs the next round of

funding, as the low loss corrugated waveguide costs $20K. But we can already achieve good results with spin ½ species as well, this gets discussed further below.

3. Due to the line narrowing at higher fields, high-spin Kramers ions also exhibit higher sensitivity advantage than other paramagnetic species. This must be mentioned. I expect that the authors did already compare sensitivity for the trityl sample (used in the SM for determining microwave field amplitude) between the commercial X-band spectrometer and the instrument described here. The result should be reported. If this has not yet been done, this experiment needs to be performed. Even if the title explicitly mentions high spin metal centres, the readers should be informed that the instrument is (probably) less attractive for organic radicals.

Yes, we are targeting high spin metal centers for a specific reason, and a lot of our own bioinorganic chemistry targets are in this domain, so it is important for us to do so. Organic radical signals, such as those we are very interested in our enzyme studies, will indeed broaden, but in a "good fashion" that affords higher g-resolution, which will be excellent for orientation selection ENDOR etc. even if the signal intensity is not increased by the same degree as the transition metal ions. And for S=1/2 signals. we will continue to have the advantage of near unity spin populations and the resultant large magnetization.

We will have to explore how low in temperature we can go before the spin lattice relaxation time for radicals becomes problematically long. We also have the large sample volume in the resonator free design, and the ability to carry out new experiments in the near future, such as spectroelectrochemistry, offers high promise for S=1/2 radical species. Less obvious is focusing on S=1/2 metal centers such as Cu(II) which have well resolved g-values at lower field (lines 292-304). These spectra will be very broad at 263 GHz, but we will also do a careful comparison with these more g-anisotropic ions going forward. But the bigger picture point is we, and the community in general, have ideal lower field pulse instruments for studying those classes of S=1/2 samples, and the 263 GHz will provide a big advantage for some targets and less so for some others. In general, we have no real difference of opinion here with the reviewer, and the manuscript is rewritten to make this clearer. A final comment about the trityl radical. We obtained some trityl for doing the specific Rabi oscillation measurement, but as a test sample it really offers no further interest to us as it's so very different from the biological radicals, such as tyrosine and tryptophan and substrate intermediates that we plan to focus on. Comparisons of low field and 263 GHz tyrosine and tryptophan radicals, for which we have much experience, are much higher on our priority list.

4. The different population of the different states for a high spin metal ion (discussed in the SM) allows for determination of the sign of the zero-field splitting from the asymmetry (more intensity low field than high field or vice versa). This should be mentioned. Apparently, the authors even have examples with different sign (EDTA and the PsaA complex). The signs should be given for these cases.

Yes, we have added a sentence in the main manuscript mentioning the ability to discern the signs of the zero-field splitting (Caption Table 1). There is also additional discussion added to the Supplementary Materials.

5. The authors mention EDNMR as a potential application. How likely is it that this will work out? Transition moments of formally forbidden transitions are lower at higher field and the transition Rabi frequency for the allowed transitions is substantially lower than for W-band spectrometers capable of EDNMR. At least this caveat should be mentioned.

The theory of EDNMR does recommend that we still want a good ratio between the nuclear Zeeman and the hyperfine to get substantial forbidden transitions [4]). We suggest that the 263 GHz would then be perfect for low γ *nuclei detections where we can still get a good Zeeman/hyperfine ratio. Additionally, the Takahashi group has successfully performed, [5] though they used an optical readout since they could not form echoes with their long microwave pulses. Their work provides some precedent that EDNMR at 263 GHz could work, and our instrument has the advantage of short pulses for an echo-readout EDNMR. Moreover, we are partnered with Steve Hill (for example on my NSF proposal under review) to make exactly these important comparisons with the NHMFL HiPER instrument. In fact, he has specifically asked us to collaborate on a new project where our much higher bandwidth will allow certain experiments he can't do on the high power W band with its lower bandwidth amplifier. A point to "echo" again here is this instrument provides a somewhat different new tool that can complement other advanced instruments in the community, and comparisons with shared samples will indeed be useful.*

6. Please point out in the main text that the Cr(III) signal is also visible at X-band. If the Mn(II):MgO sample is similar to the one in my lab, I believe that the putative V(II) signal can also be seen with CW EPR at X-band frequencies. The authors should check that and if so, mention this**.**

Yes, we have added a sentence describing the visible Cr(III) signal at X-band in the caption of Figure 4. Additionally, in the SM, we have added additional discussion and figures describing the visibility of both Cr(III) and V(II) at X-band, Q-band, and 263 GHz. See Supp. Fig. 25 in Supplementary Materials.

7. In the main text, the spin state for V(II) and Co(II) should be mentioned.

We addressed this omission (line 161-163).

8. In Fig. 4, please mention that the temperature is 100 K as well for the X-band experiment.

We addressed this omission.

9. In the SM, the probehead is called a cryoprobe. This is misleading. The term

cryoprobe is used in NMR for probes with a cryogenic low-noise amplifier. Please avoid using this established term for something else.

We addressed this and changed the terminology for the probehead.

10. In the SM in the equation below line 145 a spin operator is missing.

We addressed this omission.

11. In the SM, all equations on p. 18 mix a Hamilton operator with energy eigenvalues.

We fixed this issue.

Typos:

l. 255: "or or" delete one "or"

We fixed this typo.

Reviewer #2 (Remarks to the Author):

This paper reports the application to Mn(II) of the 263 GHz amplifier that was described in the IEEE Transactions in 2023. Significant advantages for the study of high-spin metal systems are demonstrated at each increase in EPR microwave frequency and magnetic field. Direct comparison of the performance on the new spectrometer with the commercially available 263 GHz spectrometer is provided, with explanation of the differences.

The parts list and the photographs of the spectrometer in the supplementary materials are crucial to understanding the paper. *We appreciate that the detained SM information is considered crucial by the reviewer. Design engineer Dr. Paul Stucky felt that really added to the paper and allows others to duplicate our work, which is a major goal of its dissemination.*

There will be general interest in the paper and it should be published.
Minor revision in accordance with the following comments will enhance the paper.
We appreciate that this reviewer is supportive of publication in Nature Communications with minor revision.

The better signal-to-noise achieved with the new spectrometer relative to the Bruker spectrometer is well-demonstrated, but it is also necessary to discuss the capabilities that were given up in the new design. One key feature is that the temperature and atmospheric environment of the sample cannot be controlled while the sample is loaded into the holder (figure S14). This eliminates use with many biological systems for which the high frequency would be desired. However, the text in line 213 refers to an "Open 5

mm diameter sample." Please provide a corresponding picture of how the 5 mm sample can be used. Why "open"? Is this what is referred to separately as a teflon cup? Overall, clarification of limitations on sample geometry and handling is essential.

These comments are all addressed in the revision:

We added the following text on page 3, line 101 of SM related to handling bio samples and actual improvements to trapping enzyme intermediates and other transient kinetic samples: "The gold reflector plate allows for simple sample handling for loading at cold temperatures. A simple setup that uses cold baths, such as liquid N_2, enables conditioning of the reflector plate to maintain the temperature of the sample that has already been flash-frozen prior to loading, depicted in Supplementary Figure 16. Hence, samples can be flash frozen under different conditions appropriate for the sample/protein of interest and carried over to the probehead while maintaining its frozen status." Some discussion of the flash freezing directly into gold-plated wells is including in the main text (line 224-230).

Additionally, we added the following Supplementary Figure 15 related to some Teflon and glass sample containers we have used, both fitting in our 5mm sample space geometry and Figure 16 shows our loading and freezing geometry.

In figure 6 very little of the Mn(II) intensity is in the 6 sharp lines. The dotted lines show how the overall pattern is a sum, but have the field-swept spectra ben acquired at different pulse powers to optimize for each different transition to substantiate the assignments? Since the focus of the paper is the information that one can acquire about Mn(II) at 263 GHz, more discussion of these spectra is expected.

We have added text in SM section 1.2, page 14, line 124, which is how we are accounting for the different transitions: "The simulations were done in EasySpin with the 'transitions' set in the 'separate' option, which allows for the spectrum to be separated by their individual transitions. Each transition was then weighted based on the effective flip angle of each transition [12]. Details of how each transition affects the Rabi nutation and flip angle are discussed in Supplementary Discussion 3.6."

[12] Clayton, J. A. et al. Quantitative analysis of zero-field splitting parameter distributions in gd(iii) complexes. Physical Chemistry Chemical Physics 20, 10470–10492 (2018). URL http://dx.doi.org/10.1039/c7cp08507a.

We plan a more detailed probing of these different transitions once we have improved thermometry with a new sensor adjacent to the sample in the probe. We only have the thermometer at the gas jet of the cryostat and have learned at low temperature (say below 10K) that the sample temperature differs from that reading (by comparing experimental field sweeps with simulations). Given that we can now achieve at our lowest temperature virtually 100% ground state populations, an excellent paper will result by studying both the pulse angle and the temperature dependence of complex signals, once we have better temperature measurements at the sample position (not a

problem at the higher temps, like the 100K used to populate all the Mn(II) S=5/2 magnetic states in much of this paper).
Additionally, we have also added more theoretical discussion on the asymmetry of the spectra in S.M. section 3.3.

Given the high Fe concentration in the MgO sample (Supplementary Table 1), why is no Fe EPR signal observed?

In previous publications, Fe in MgO is expected to have S=2 transitions in 5-6 T range, which is outside the magnetic field range we have studied so far We definitely should return to, but it I do note that what we want to do with the analytical MgO test sample is mostly reported in this paper, and we wish to focus more on frozen solution and biological samples, where of course Fe(II) would be an important target. We discuss more in the SM now with references to some key papers:

> *[20] Abragam, A. & Bleaney, B. Electron Paramagnetic Resonance of Transition Ions (Oxford University Press Academic UK, 2012), illustrated edn.*
> *[21] Krzystek, J., Ozarowski, A. & Telser, J. Multi-frequency, high-field EPR as a powerful tool to accurately determine zero-field splitting in high-spin transition metal coordination complexes. Coor-dination chemistry reviews 250, 2308–2324 (2006). URL https://linkinghub.elsevier.com/retrieve/pii/S0010854506000737.*
> *[22] Telser, J. et al. High-frequency/high-field EPR spectroscopy of the high-spin ferrous ion in hexaaqua complexes. Magnetic Resonance in Chemistry 43 Spec no., S130–9 (2005). URL http://dx.doi.org/10.1002/mrc.1689.*

It is appropriate that the paper state that the Mn in MgO sample that they used is no longer available from the commercial source, but it would be useful to state that almost any commercial CaO (and probably many other divalent oxides) has a Mn(II) EPR signal that could be used instead.

We added this information to the main text.

Figure 4 caption should explain the high-intensity signal.

Yes, we have added a sentence in the Figure 4 caption describing the high-intensity signal as originating from Cr(II). Furthermore, we have added discussion and simulations to reflect that assignment in Supp. Fig. 25.

Reviewer #3 (Remarks to the Author):

This manuscript presents a significant instrumental development in pulse high field EPR, 263 GHz, featuring high power (10 W), which is exceptional at these frequencies. This is coupled with a bandwidth of 5.6 GHz of the power amplifier and a resonator free design that allows for a 12 μl sample size. The construction and design of the power

amplifier (already published) and the spectrometer are impressive and novel and open many new possibilities. The properties of this new spectrometer are demonstrated on rather standard high-spin Mn(II) (S=5/2) complexes and two Mn(II) containing proteins. The motivation is the well-known fact that linewidth of the central transition is inversely proportional to the spectrometer frequency, which increases sensitivity and resolution. While the prospects of this new spectrometer are excellent, none of these are demonstrated in the work. Therefore, this work, which is mainly instrumental in character, should be published in journals like Rev. Sci. Instruments or JMR or MR.

In our view the novelty and impressive performance of this instrument, including on dilute metalloproteins, makes this an appropriate manuscript for Nature Communications, as suggested by the Nature editors when they suggested this after our initial submission to Nature. Moreover, the other reviewers support this view as well. The design and results to date nicely fit the length of the communications format.

Below we list concerns and comments that should be addressed before publication in any journal.

1. The authors demonstrate the performance of the spectrometer on trivial samples and the simplest pulse EPR experiment, Echo-detected EPR, that yields the EPR spectrum, which can be obtained with CW-spectrometers as well. To convince the reader that this new spectrometer indeed stands out to its promise the authors should show pulse EPR results that emphasize the prospect of the high power and band width that are hard to achieve with existing spectrometers (W-band and higher) in terms of resolution.

We respectively disagree with this perspective. As noted earlier, the whole point of the paper is to introduce high power pulse EPR to a frequency approximately X3 higher than done before, using an innovative resonator free approach that is made possible by the high 263 GHz pulse power afforded by the new TWT amplifier. In our view, the trace paramagnetic elements in the MgO solid sample actually provide a superb analytical standard, which allows us to gauge performance, both sensitivity and resolution, on a robust standard and stable sample and provides an excellent launch point to the study of to the frozen solution Mn(II) complexes and proteins we also present here. Yes, this is a field swept electron spin echo study but that serves as the basis for ALL the advanced pulse EPR experiments we proposed to do, such as ENDOR and EDNMR, all beyond the scope of this first communication but all which we will implement in the coming months once, particularly after we secure further grant support.

2. To show the “breakthrough” nature of the new instrument the authors use a comparison with X-band spectrometers. This is an obsolete comparison. The comparison should be done with state of the art commercial Q-band spectrometers that are widely available and more importantly with 95 GHz spectrometers (homebuilt and commercial) , which have been around for a long time and reported a plethora of advanced pulse EPR applications such as ENDOR, DEER, HYSCORE, ELDOR-detected NMR etc, applied to both S=1/2 and high-spin systems (none of this is referenced). The information derived from the echo-detected EPR examples shown in this work is easily obtained at W-band or with the 263 GHz Bruker spectrometer.

*The fact is that the Bruker X band pulse instrument is the only **widely** available pulse EPR standard, which is why we focused on it. There are all sorts of pulse Q band instruments, all pretty different, with no one real standard used in the pulse EPR community. For example, we have carried out many experiments over the years with a modified low power Bruker Q band with a solid state amplifier of a few watts power and an historic Feher lab CW ENDOR probe that he gave us years ago. There are all sorts of Q band systems. Look at Brian Hoffman's lab as another example. We picked the Bruker X band for comparison because it's the only real standard available widely (including the fact that we have one ourselves). Nevertheless, we have followed up on the suggestion to include parallel results from a recent Q band pulse instrument, in the lab of Professor Glenn Millhauser of UC Santa Cruz, and include an analysis now in the SM section 3.5. See further new discussion in main text (lines 282-291).*

We would have liked to include much more about the history of pulse EPR at higher frequency, but this historical text was not included due to the short format of Nature Communications. We do talk explicitly about the high power W band instruments, particularly the HiPER design (lines 83-85) We have active plans in our recent NSF proposal to compare performance of this instrument with this pulse W band at Florida State, so these experiments will be performed in the future. We will reach out at some point in the near future to one of the labs with the 263 GHz Bruker, but the difference between the instruments is already clear. If you are limited to approximately 30 nl sample by the tiny 263 GHz resonator the Bruker system performs well. But typically we are not limited to such small sample volumes, and we can run approximately 1000X as many spins in a sample in our design. Moreover, the open resonator-less design gives us access to a number of experiments one cannot do in such tiny EPR tubes, like building robust electrospectrochemistry cells, sample holders designed to flash advance photochemical systems in a thin 2D disk geometry, and a very easy robust way to do rapid freeze quenching in gold plated cups in a fashion similar to the well-known Simon de Vries method of RFQ on cryocooled metal, with the further advantage of not having to then transfer the RFQ "powder" to tiny EPR tubes. We have a broader view of this instrument than the reviewer is coming from in these comments, so we need to explain this better, so in the revision we have rewritten much of the discussion section with this in mind for additional clarity. Again, we are not trying to build a spectrometer than makes all other spectrometers obsolete, but to show an alternative mode for bringing higher power pulse EPR to higher frequencies with good bandwidth. Indeed, we plan to use this new instrument for many years to come, and the fact that we haven't done everything the instrument will be able to do yet should not be held against our publishing this new novel work in Nature Communications.

3. The new spectrometer indeed allows relatively short $\pi/2$ pulses for Mn(II), 30 ns, because of its high spin nature. The pulse length for S=1/2 is longer, 100 ns, which is not that attractive. A Bruker 263 GHz spectrometer is commercially available and uses a resonator which limits the sample size and band width. Nevertheless, it affords a high B1 that yields a $\pi/2$ pulse of 30 ns for S=1/2 . Very nice pulse ENDOR measurements were reported with this spectrometer (Bennati's lab).

Firstly, the paper is indeed focused on high spin ions which are important targets for high field pulse EPR. We have no issue of course with the low power Bruker instrument, which no doubt does a fine job for limited volume samples in tiny EPR tubes. We are demonstrating a different approach here, also valid. We achieve excellent S/N in test samples, including dilute proteins, without a resonator that allows for more sample volume than can be obtained with a high frequency resonator. So it's a bit apples vs oranges, and we think it is important to show that another approach, enabled by a big advance in vacuum electronics amplifiers and high frequency works well. This reviewer is specifically concerned about pulse length but our prime concern is very good signal to noise on dilute samples which is in fact nicely demonstrated here. And it is worth noting that longer pulses have some advantage in terms of resolution as long as the S/N remains high.

4. There is no discussion about sensitivity limits. Please add.

We added more discussion about sensitivity limits for OX071 page 19, line 152 of SM and for Mn(II) in page 24, section 3.4, line 232 of SM:

Other minor issues:
1. Is phase cycling implemented, how ?

We are currently working on adding phase cycling, this involved phase shifting at the lower frequency before mixing and multiplying to 263 GHz. All the hardware is in place for this so this will be implemented quite soon.

2. What is the deadtime of the spectrometer**?**

The current observed deadtime ranges between 100-150 ns.

3. The bandwidth of the amplifier is given, but not of the full bridge.

The 250mW VDI AMC that is the input driver to the TWT is the formal bandwidth limit at 5GHz (not 5.6GHz). We can improve with a future VDI purchase along with high pulse power tolerance, as in a current NSF proposal.

4. What is the cooling system and what is the lowest possible temperature?

It is a Spectrostat CF DY cryostat from Oxford, controlled by the Mercury system. The lowest possible temperature is 1.37 K based on testing of the cryostat before shipping.

References

[1] Pan, P.; Zheng, Y.; Li, Y.; Song, X.; Feng, Z.; Feng, J.; Britt, R. D.; Luhmann, N. C., Jr. Demonstration of a 263-GHz Traveling Wave Tube for Electron Paramagnetic Resonance Spectroscopy. *IEEE Trans. Electron Devices* **2023**, *70*(11), 5897–5902. https://doi.org/10.1109/ted.2023.3312230

[2] Takahashi, S.; Brunel, L.-C.; Edwards, D. T.; van Tol, J.; Ramian, G.; Han, S.; Sherwin, M. S. Pulsed Electron Paramagnetic Resonance Spectroscopy Powered by a Free-Electron Laser. *Nature* **2012**, *489*, 409–413.

[3] Cruickshank, P. A. S.; Bolton, D. R.; Robertson, D. A.; Hunter, R. I.; Wylde, R. J.; Smith, G. M. A Kilowatt Pulsed 94 GHz Electron Paramagnetic Resonance Spectrometer with High Concentration Sensitivity, High Instantaneous Bandwidth, and Low Dead Time. *Rev. Sci. Instrum.* **2009**, *80* (10), 103102. https://doi.org/10.1063/1.3239402

[4] Cox, N.; Nalepa, A.; Lubitz, W.; Savitsky, A. ELDOR-Detected NMR: A General and Robust Method for Electron–Nuclear Hyperfine Spectroscopy. *J. Magn. Reson.* **2017**, *280*, 63–78. https://doi.org/10.1016/j.jmr.2017.04.006

[5] Fortman, B.; Mugica-Sanchez, L.; Tischler, N.; Selco, C.; Hang, Y.; Holczer, K.; Takahashi, S. Electron–Electron Double Resonance Detected NMR Spectroscopy Using Ensemble NV Centers at 230 GHz and 8.3 T. *J. Appl. Phys.* **2021**,*130* (8), 083901. https://doi.org/10.1063/5.0055642

Response to reviews, Britt et al

REVIEWER COMMENTS

Reviewer #1 (Remarks to the Author):

The authors have revised the manuscript in a satisfying way, addressing my main concerns. I agree with the arguments that the achieved advancement is substantial and justifies publication in Nature Communications (as I already recommended for the first version). I now recommend publication of the manuscript in its present form.

We greatly appreciate the view of Reviewer #1 that the manuscript was publishable in its present form as of the last submission.

Reviewer #2 (Remarks to the Author):

the authors responded well to the comments of all reviewers.
the revised paper and supplementary materials are ready for publication.

Please fix the spelling of P. Hofer in reference 10. CORRECTED

We greatly appreciate the view of Reviewer #2 that the manuscript was publishable in its present from present form as of the last submission. I have corrected the spelling in ref 10.

Reviewer #3 (Remarks to the Author):

We appreciate the authors' efforts in revising the manuscript and responding to our comments. While several minor technical issues have been addressed, the authors have disagreed with our concerns and have chosen not to revise the manuscript accordingly. Although they have provided lengthy explanations of their position, we do not find them sufficiently compelling to alter our overall assessment of the manuscript. Consequently, our evaluation remains unchanged. We also note that discussions of plans and the status of related grant applications are relevant to the current manuscript.

1. We agree that the prospects of such a high-power spectrometer are promising and impactful. However, for a publication in a high-impact, broad-scope journal, we would expect at least one clear, convincing example (sample or experiment, not a standard sample) demonstrating the combined advantages of high frequency and high power over existing spectrometers.
2. When presenting a newly developed, state-of-the-art spectrometer, it is important to benchmark its performance against existing leading instruments. In this case, a comparison with high-frequency pulsed spectrometers (e.g., 95, 140, 180, 195, 263, or 390 GHz systems) would be more appropriate. Comparisons limited to low- and medium-frequency pulsed X- and Q-band spectrometers may not fully capture the advancement, even if those systems are widely available. While we understand that such comparisons may be planned for future work, their absence in the current manuscript weakens the conclusions.

Reviewer #4 (Remarks to the Author):

I co-reviewed this manuscript with one of the reviewers who provided the listed reports. This is part of the Nature Communications initiative to facilitate training in peer review and to provide appropriate recognition for Early Career Researchers who co-review manuscripts.

We respectively disagree with the view of reviewer 3 (and 4) that we need to benchmark our novel instruments performance vs some wide set of existing high frequency instruments.

In this revision I revised the introduction extensively, given a succinct but thorough review of the history of high field *PULSE* EPR, explaining the unique importance of the W band (95 GHz) instruments as we discussed, since that's the highest frequency with high power amplifiers available before our TWT development. I describe how beyond W band frequencies one has had to work with low power solid state power levels (including our 130 GHz instrument) which necessitates high Q cavities to get sufficient B1 field and excitation bandwidth. But this invariable scales to dramatically smaller sample volumes for higher frequency instruments ($V \sim 1/f^3$). Our new high bandwidth high power amplifier allows us to carry out high sensitivity pulse EPR without the use of the tiny low volume resonator. We illustrate the system performance on a series of S-5/2 Mn(II) complexes ranging from Mn(II) in a simple diamagnetic host solid to Mn(II) in frozen solution and Mn(II) in metal sequestering proteins, along with high quality simulations and interpretations as to how the spectra change across the series. We finish with a brief description of a number of advantages that this resonator-free design opens up for different classes of pulse EPR experiments.

In this revision we decided to deemphasize the direct S/N comparisons of the resonator-free 263 GHz Pulse instrument to our X-band pulse Bruker with resonator, given the very valid comments of R3. We still show the data to make the point that our high power/resonator-free approach works very well, but we deemphasize the direct S/N comparison to pulse X-band in the writing.

Major rewriting to achieve these goals include most of the introduction (lines ~62-123) and a rephrasing of the 263 GHz vs X band performance in discussion (327-331). I do feel the manuscript is greatly improved in this version. No figures or data are changed, just the focus of some of the writing as denoted here.
